# Regiodivergent condensation of 5-alkoxycarbonyl-1*H*-pyrrol-2,3-diones with cyclic ketazinones en route to spirocyclic scaffolds

**DOI:** 10.3762/bjoc.13.218

**Published:** 2017-10-19

**Authors:** Alexey Yu Dubovtsev, Maksim V Dmitriev, Аndrey N Maslivets, Michael Rubin

**Affiliations:** 1 Department of Chemistry, Perm State University, ul. Bukireva 15, Perm 614990, Russian Federation; 2 Department of Chemistry, North Caucasus Federal University, 1a Pushkin St., Stavropol 355009, Russian Federation; 3 Department of Chemistry, University of Kansas, 1251 Wescoe Hall Dr., Lawrence, KS 66045-7582, USA

**Keywords:** nitrogen heterocycles, oxygen heterocycles, pyrrolediones, spiro compounds, synthetic methods

## Abstract

The condensation of 5-alkoxycarbonyl-1*H*-pyrrolediones with cyclic ketazinones was systematically investigated. It was discovered that the regioselectivity of this reaction can be easily swapped between two alternative directions affording derivatives of partially hydrogenated indole or benzofurane. The control of this regioselectivity is efficiently governed by steric effects at the hydrazone moiety of the ketazinone reagent.

## Introduction

Molecular structures based on partially or exhaustively hydrogenated indole and benzofuran cores are omnipresent in nature. Both types of ring systems are found in a variety of important biologically active natural products [1–20], which continue to remain in the focus of attention for many research groups worldwide as targets for total synthesis and to serve as inspiration for exercises in drug design. Although many preparative methods of assembly of these structural units have been demonstrated, the development of new efficient and highly selective synthetic tools is always desired. From this prospective, we have become greatly interested in the chemistry of 1*H*-pyrrole-2,3-diones, polyfunctional building blocks that have great potential for the synthesis of heterocyclic structures. Indeed, these highly electrophilic cyclic vinylogous amides are known to undergo facile nucleophilic additions [21–25], sometimes accompanied with subsequent pericyclic rearrangements [26–33]. Not surprisingly, these versatile synthons have been successfully employed in the target-oriented synthesis of pyrrole-based natural alkaloids [34–38]. Herein we wish to report a new synthetic route towards spirocyclic scaffolds possessing partially hydrogenated indole or benzofuran cores. The featured approach is based on the highly efficient regiodivergent spirocondensation of 5-alkoxycarbonyl-1*H*-pyrrole-2,3-diones (serving as 1,2-bis-electrophiles) with cyclic ketazinones (serving as either 1,3-*C,N*- or 1,3-*C,O*-bis-nucleophiles).

## Results and Discussion

**Scheme 1:**
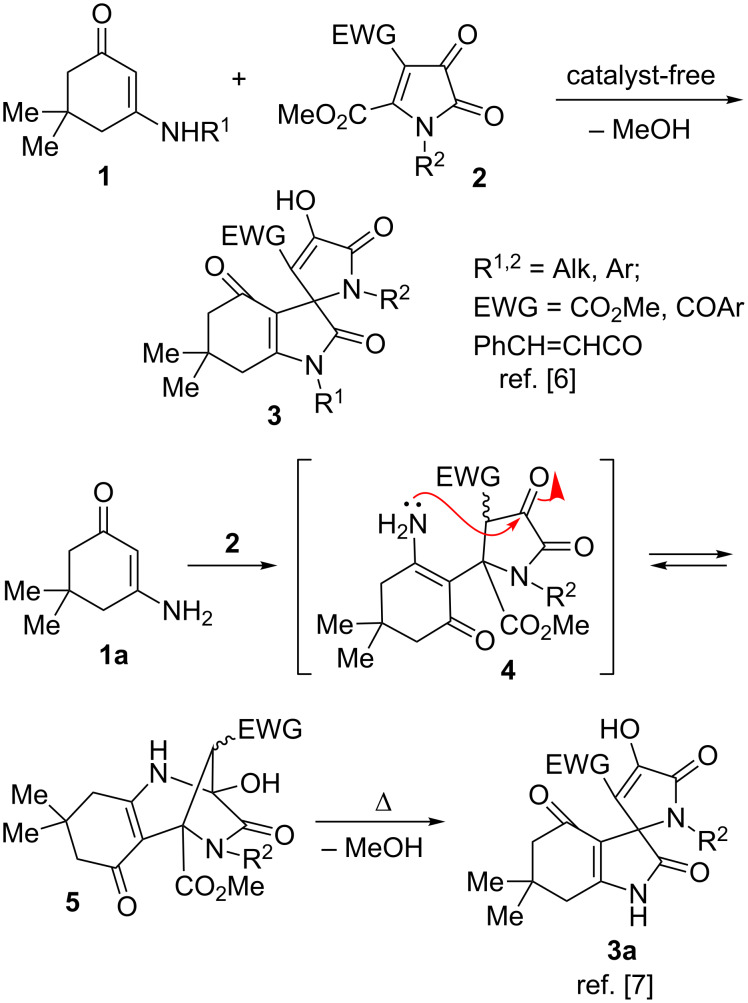
Spirocyclization of enamines with 5-methoxycarbonyl-1*H*-pyrrolediones.

Previously, we demonstrated a convenient approach towards spiro[indole-3,2’-pyrroles] **3** based on catalyst-free cyclocondensation of six-membered cyclic enamines **1** (vinylogous secondary amides) with 5-methoxycarbonyl-1*H*-pyrrolediones **2** [39–41]. This transformation involved the Michael addition of an enamine to the α,β-unsaturated carbonyl fragment of a pyrroledione and subsequent 5-*exo-trig* intramolecular nucleophilic attack of the amine moiety on the ester substituent (Scheme 1). Interestingly, it seems that the substitution at the nitrogen atom in structure **1** is very crucial in governing the desired reactivity. Indeed, our previous attempts to expand the substrate scope to include “vinylogous primary amides” **1a** resulted in the discovery of an alternative mechanistic pathway. Apparently, in this case the primary amine moiety in intermediate **4** preferred a nucleophilic attack on the keto function, affording bridged hemiaminal structures **5** as kinetic products (Scheme 1) [42]. Upon extended heating, however, recyclization into the thermodynamically more favorable “normal” spirocyclic products **3a** occurs.

**Scheme 2:**
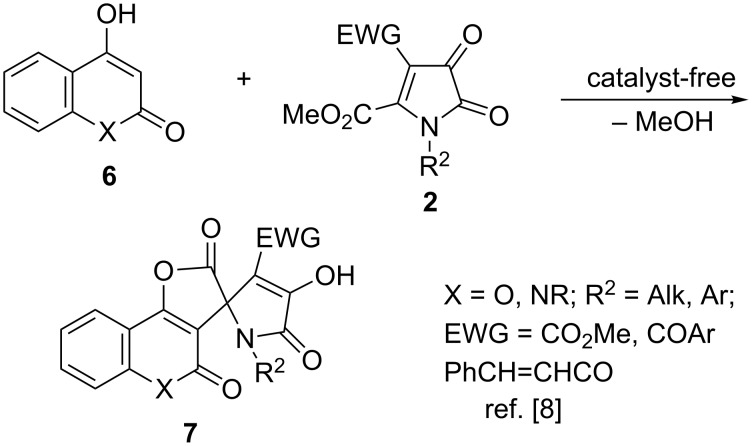
Non-catalyzed spirocyclization of enoles (vinylogous carbonates and carbamates) with 5-methoxycarbonyl-1*H*-pyrrolediones.

It should be further noted that in contrast to reactions of enamines, which readily provide the corresponding adducts with pyrrolediones in the absence of catalysts, the similar transformation involving enols **6** (vinylogous carbonates and carbamates) normally requires more forcing conditions, but usually can be facilitated by addition of catalytic amounts of organic base (Scheme 2) [43].

**Scheme 3:**
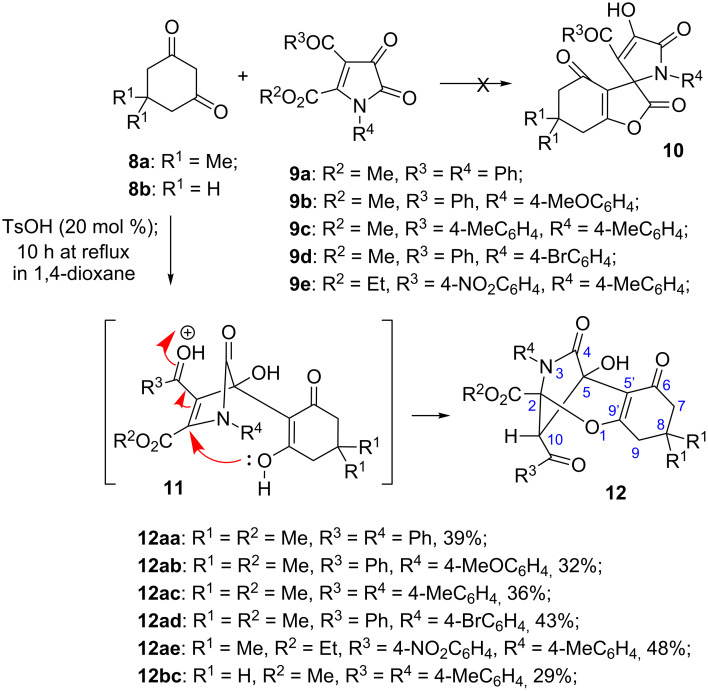
Acid-catalyzed spirocyclization of enoles (vinylogous carboxylates) with 5-alkoxycarbonyl-1*H*-pyrrolediones.

Interestingly, we figured out that the presence of heteroatom X in the structure of enol **6** is important for the normal course of the spirocyclization reaction. Our multiple attempts to carry out this transformation with the participation of enolates generated from cyclohexane-1,3-diones **8** (vinylogous carboxylates) in the presence of bases were unsuccessful. This reaction did not proceed in the presence of weak bases (such as tertiary amines), while the use of stronger bases (hydroxides or alkoxides) caused decomposition of the base-sensitive 1*H*-pyrrole-2,3-dione moiety **9**. An attempt to perform the reaction in the presence of catalytic amounts of Brønsted acid (TsOH) also did not provide the spirocyclic products. Instead, bridged 1,3-oxazepine products **12** were formed in marginal yields, resulting from an initial aldol reaction involving the carbonyl group at C-3 of pyrroledione **9** and a subsequent intramolecular 6-*endo*-*trig O*-nucleophilic attack of the enol species at a conjugate unsaturated ketone moiety in the five-membered ring of intermediate **11** (Scheme 3).

**Figure 1:**
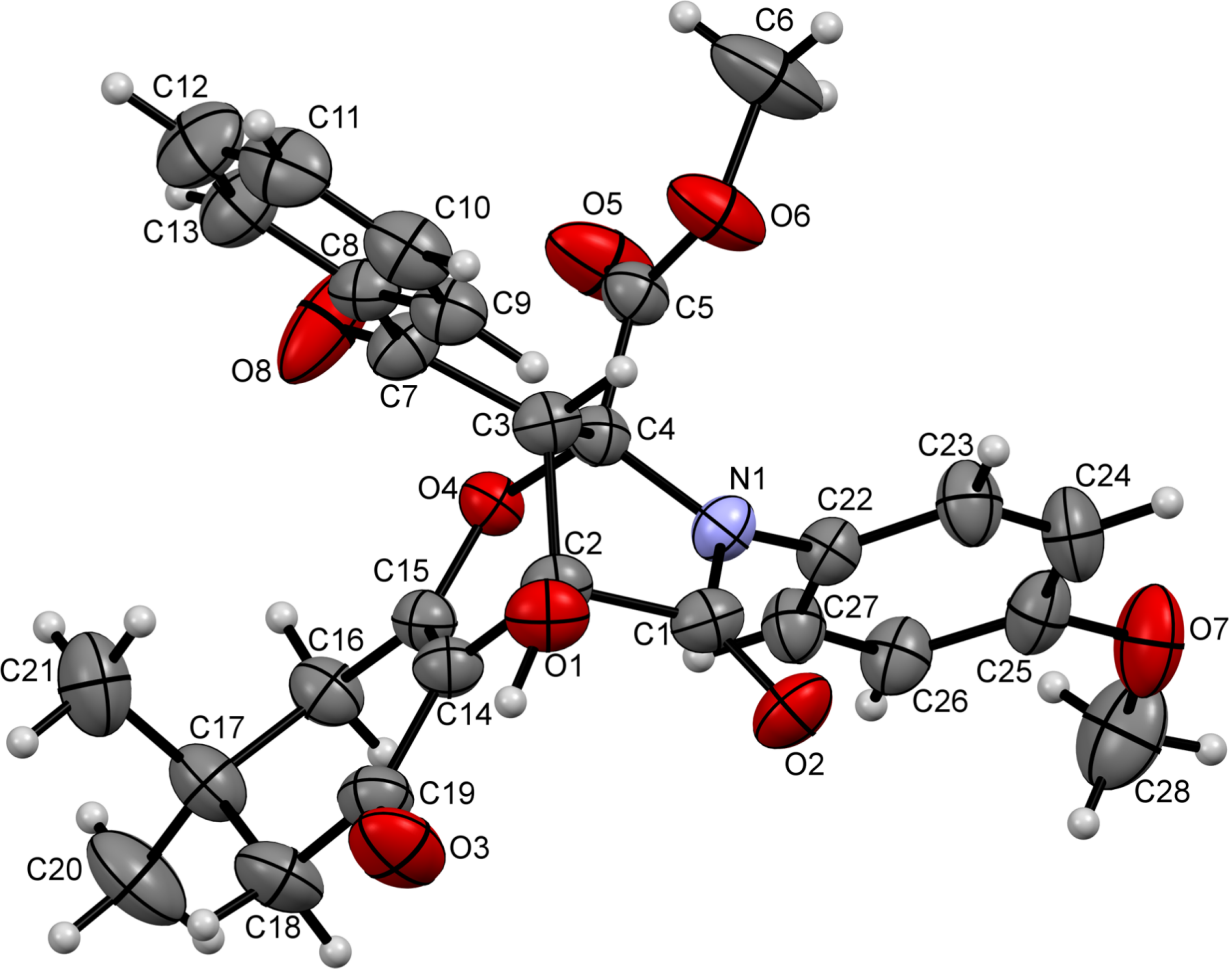
ORTEP drawing of compound **12ab** (CCDC 1546062) showing 50% probability amplitude displacement ellipsoids.

Although the reasons for such divergence in the reactivity are not completely understood, one could argue that the lower nucleophilicity of enolates derived from **8** as compared to that of enamine **1a** could be responsible for this effect. Indeed in this case, the product of 1,2-addition of the *C*-nucleophile to the most reactive keto function and subsequent nucleophilic attack by the *O*-enolate on the conjugate C=C bond activated by two electron acceptors could become more preferable as compared to the alternative “normal” pathway, leading to adducts **10** and involving Michael addition followed by intramolecular transesterification. Remarkably, the resulting bridged products **12** have reversed regiochemistry as compared to the earlier-described cycloadducts **5** (Scheme 1). It should also be pointed out that all of these products were formed as single 10-*endo* diastereomers (see Scheme 3 for atom numbering). This configuration was unambiguously confirmed by a single crystal X-ray crystallography of compound **12ab** (CCDC 1546062) shown in Figure 1.

**Scheme 4:**
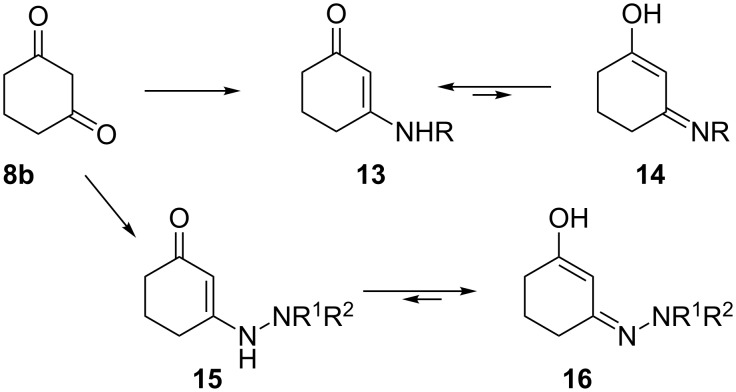
Formation of mono-imines and mono-hydrazones of 1,3-cyclohexanediones and tautomeric equilibrium between enol-imine and keto-enamine forms.

Puzzled by this unexpected reactivity, we reasoned that the enolate moiety can be activated towards the desired spirocyclization via conversion of 1,3-diones **8** into mono-hydrazones. Indeed, while mono-imines of these ketones strongly prefer keto-enamine tautomeric form **13** over enol-imine form **14** (Scheme 4) [44–45], the corresponding hydrazones have been reported to favor enol-hydrazone tautomer **16** (Scheme 4) [46–48].

**Scheme 5:**
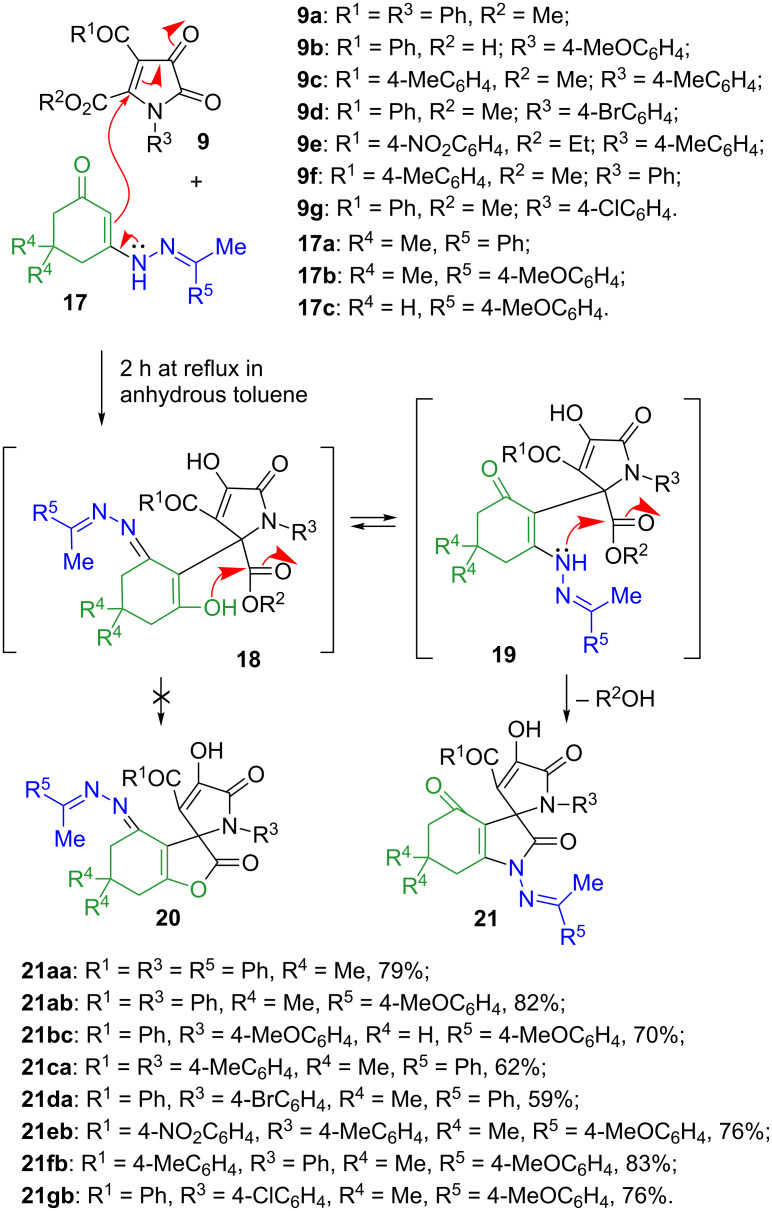
Spirocyclizations involving non-bulky ketazinones **17** and 5-alkoxycarbonyl-1*H*-pyrrolediones **9**.

Keeping this in mind we decided to test the reactivity of ketazinones **17** that were obtained via condensation of cyclohexanediones **8** with hydrazone of acetophenone. We anticipated the formation of spirolactones **20** in this process, resulting from intramolecular transesterification involving the enol moiety in tautomeric form **18** (Scheme 5). Surprisingly, an alternative direction of spirocyclization involving the reaction of tautomeric form **19** and affording lactam rings proceeded exclusively. The corresponding spiro[indole-3,2’-pyrroles] **21** were obtained exclusively in good yields (Scheme 5).

**Figure 2:**
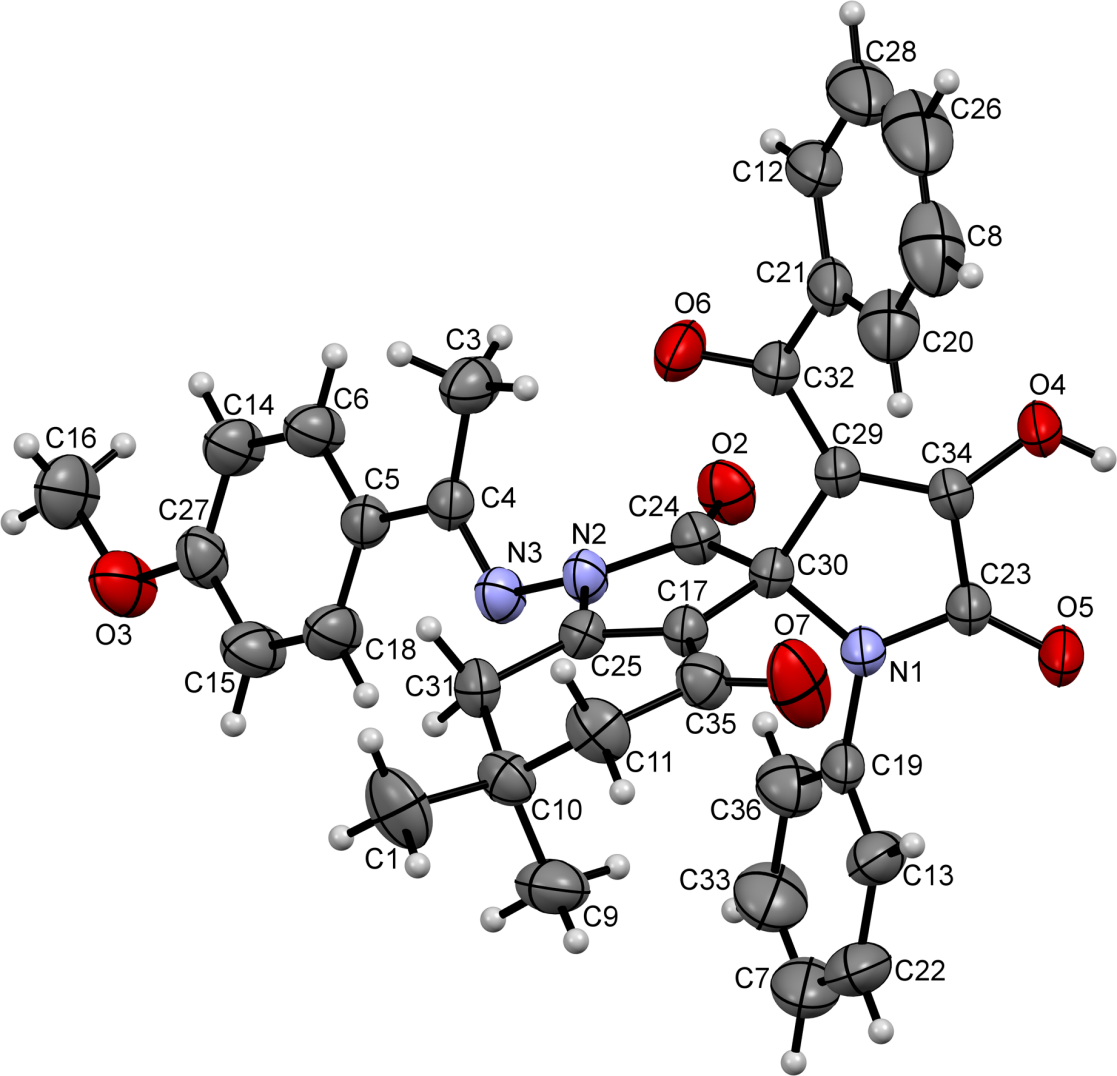
ORTEP drawing of compound **21ab** (CCDC 1546063) showing 50% probability amplitude displacement ellipsoids.

The formation of the indoline ring was unambiguously confirmed by the crystal structure of compound **21ab** (CCDC 1546063, Figure 2). It seems that the nucleophilicity of the hydrazine moiety prevailed, and the formation of the thermodynamically more favorable amide bond governed the overall direction of this transformation. It should be also taken into account that, unlike the aforementioned hydrazone structures **15** and **16**, ketazinones appear to be more stable in keto-enamine form **17**.

**Figure 3:**
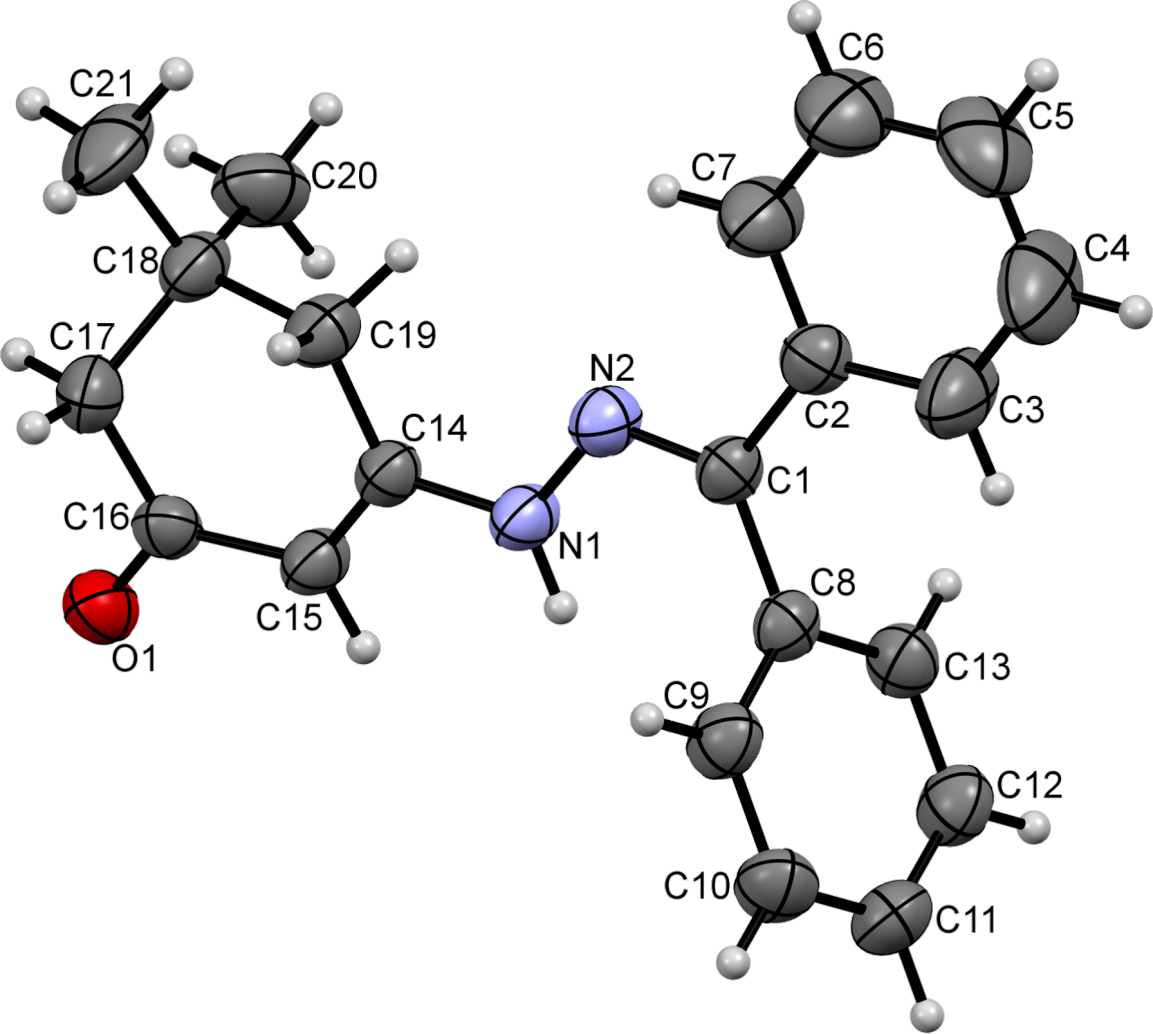
ORTEP drawing of compound **22a** (CCDC 1546065) showing 50% probability amplitude displacement ellipsoids.

**Scheme 6:**
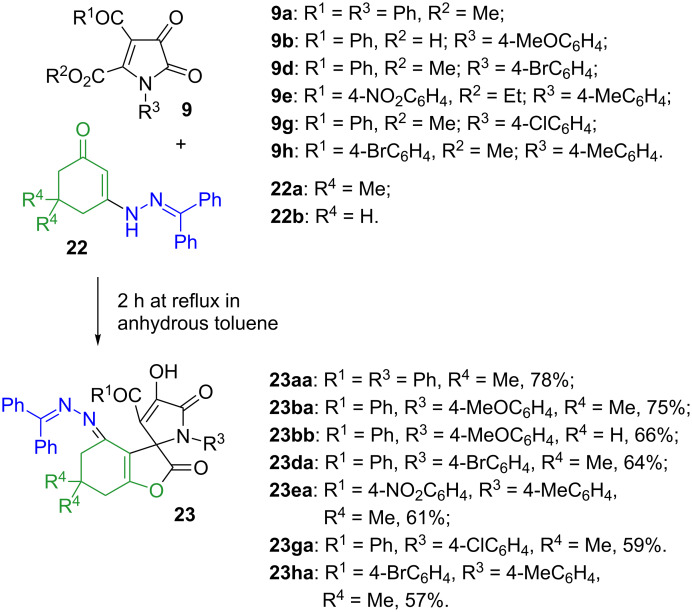
Spirocyclizations involving bulky ketazinones **22** and 5-alkoxycarbonyl-1*H*-pyrrolediones **9**.

We reasoned that the nucleophilicity of the ketazinone moiety can be substantially reduced via incorporation of excessive steric bulk, which ultimately could help us to redirect the course of the reaction towards formation of spirolactones of type **20**. To evaluate this idea, we prepared ketazinones **22** (crystal structure of ketazinone **22a** was confirmed by X-ray crystallography (CCDC 1546065, Figure 3)), derived from benzophenone and tested their reactivity with pyrrolediones **9** (Scheme 6). Gratifyingly, this reasoning was correct, as we obtained the corresponding lactones **23** as the sole products in reasonable yields (Scheme 6).

**Figure 4:**
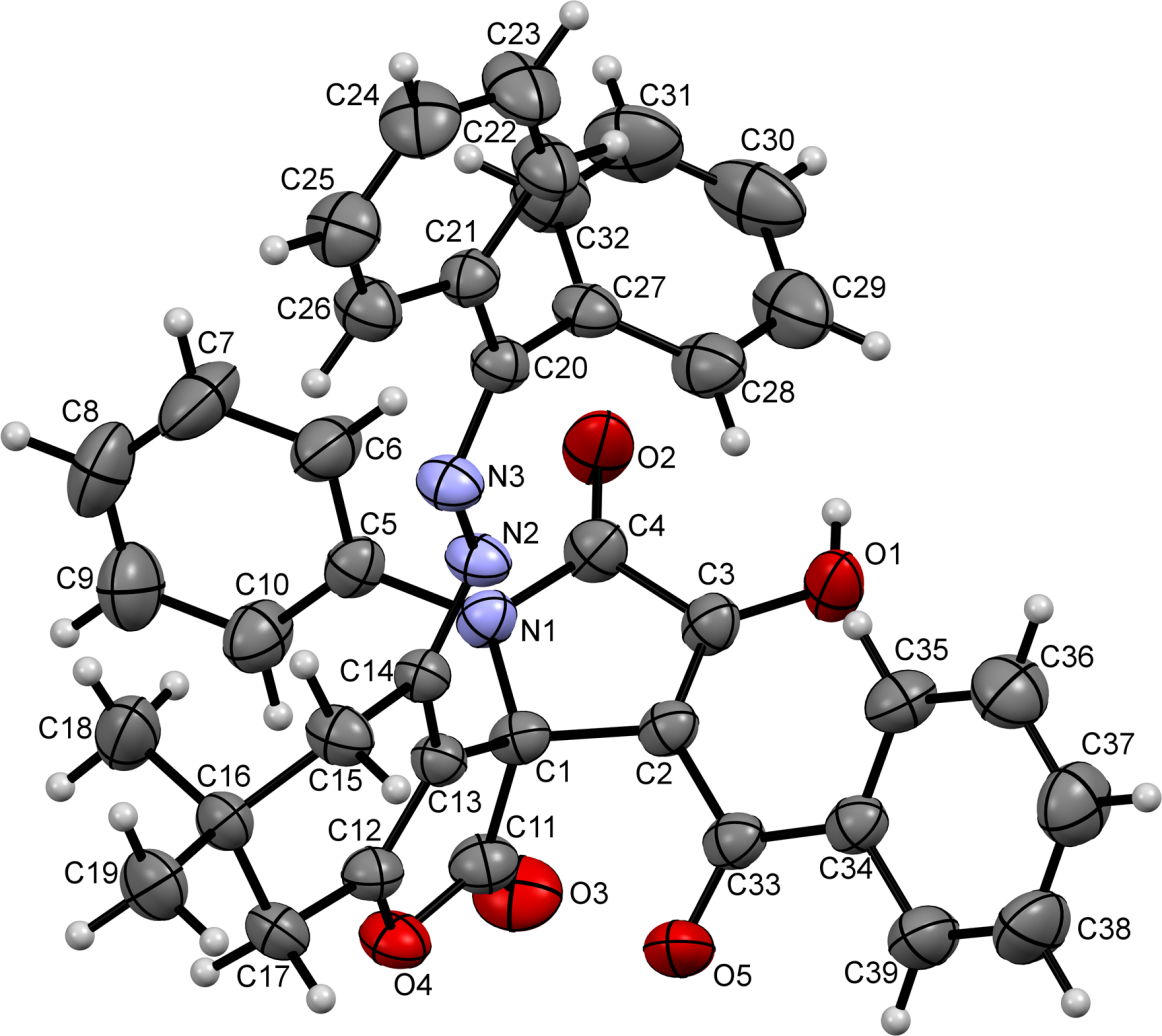
ORTEP drawing of compound **23aa** (CCDC 1546064) showing 50% probability amplitude displacement ellipsoids.

The crystal structure of compound **23aa** (CCDC 1546064) depicted in Figure 4 confirmed the formation of this elusive scaffold.

## Conclusion

In conclusion, we discovered a new mode of cyclocondensations with “inverted” regiochemistry of addition, which involved the acid-catalyzed reaction of 5-alkoxycarbonyl-4-aroyl-1*H*-pyrrole-2,3-diones with cyclohexane-1,3-diones and lead to the formation of bridged 2,5-methanobenzo[*f*][1,3]oxazepines. We also found efficient regiodivergent spirocondensation of the same pyrrolediones with cyclic ketazinones affording the formation of spirocyclic scaffolds with either hydroindoles or hydrobenzofuran moieties. Remarkably, the direction of this condensation can be efficiently switched towards the formation of either of the products by tweaking steric parameters of the employed ketazinones.

## Supporting Information

File 1 ^1^H and ^13^C NMR spectral charts and experimental procedures.

File 2 X-ray CIF files.
