# Supplementary material for: Regiodivergent condensation of 5-alkoxycarbonyl-1H-pyrrol-2,3-diones with cyclic ketazinones en route to spirocyclic scaffolds
Source: Beilstein J Org Chem. 2017 Oct 19;13:2179–85. doi: 10.3762/bjoc.13.218 (PMC5669231; doi:10.3762/bjoc.13.218)
Supplement: File 1 — 1H and 13C NMR spectral charts and experimental procedures. [file Beilstein_J_Org_Chem-13-2179-s001.pdf]

Supporting Information

for

Regiodivergent condensation of 5-alkoxycarbonyl-1*H*-pyrrol-2,3-diones with cyclic ketazinones en route to spirocyclic scaffolds

Alexey Yu. Dubovtsev[1], Maksim V. Dmitriev[1], Andrey N. Maslivets*[1] and Michael Rubin*[2,3]

Address: [1]Department of Chemistry, Perm State University, ul. Bukireva 15, Perm 614990, Russian Federation, [2]Department of Chemistry, North Caucasus Federal University, 1a Pushkin St., Stavropol 355009, Russian Federation and [3]Department of Chemistry, University of Kansas, 1251 Wescoe Hall Dr., Lawrence, KS 66045-7582, USA

Email: Andrey N. Maslivets - koh2@psu.ru; Michael Rubin - mrubin@ku.edu

* Corresponding author

^{1}H and ^{13}C NMR spectral charts and experimental procedures

Experimental

Materials and instrumentation. ^{1}H and ^{13}C NMR spectra were recorded on a Bruker Avance-III spectrometer (400 or 100 MHz, respectively) equipped with a BBO probe in $CDCl_3$ or DMSO-d_6 using TMS and HMDSO as internal standards. IR spectra were recorded with a Perkin–Elmer Spectrum Two spectrometer from mulls in mineral oil. Melting points were measured with a Stuart smp30 apparatus. X-ray crystallography was performed on an Xcalibur Ruby diffractometer. Elemental analyses were carried out on a Vario MICRO Cube analyzer. Starting 5-alkoxycarbonyl-1*H*-pyrrole-2,3-diones **9a–h** were obtained according to published procedures [S1,S2]. Anhydrous toluene and 1,4-dioxane were obtained by refluxing over molten sodium metal and subsequent distillation under dry nitrogen atmosphere. Other reagents and solvents were purchased from commercial vendors and were used as received.

Typical procedure for preparation of bridged polycyclic products 12:

Methyl 10-benzoyl-5-hydroxy-8,8-dimethyl-4,6-dioxo-3-phenyl-4,5,6,7,8,9-hexahydro-2,5-methanobenzo[*f*][1,3]oxazepine-2(3*H*)-carboxylate (**12aa**): A reaction vessel was charged with dimedone **8a** (140 mg, 1.00 mmol), 4-benzoyl-5-methoxycarbonyl-1-phenyl-1*H*-pyrrole-2,3-dione (**9a**, 335 mg, 1.00 mmol), *p*-TsOH (34 mg, 0.20 mmol), and anhydrous 1,4-dioxane (7 mL). The mixture was refluxed for 10 h, when the reaction was completed

according to TLC analysis. Then, the solvent was removed under reduced pressure and the product was obtained via column chromatography of the residual solid on Silica gel eluting with toluene/EtOAc (10:1). The titled compound was isolated as colourless crystals, m.p. 162–163 °C, R_f 0.70 (toluene/ethyl acetate 10:1) in a yield of 185 mg (0.39 mmol, 39%). ^{1}H NMR (400 MHz, $CDCl_3$) δ, ppm: 8.06 (d, *J* = 8.2 Hz, 2H, Ar), 7.77 (s, 1H, OH), 7.59 (t, *J* = 7.4 Hz, 1H, Ar), 7.46 (t, *J* = 7.9 Hz, 2H, Ar), 7.38 (t, *J* = 7.4 Hz, 2H, Ar), 7.32-7.28 (m, 3H, Ar), 4.70 (s, 1H, CH), 3.60 (s, 3H, OMe), 2.67 (d, *J* = 17.8 Hz, 1H, CH_2), 2.50 (d, *J* = 17.8 Hz, 1H, CH_2), 2.28 (d, *J* = 16.6 Hz, 1H, CH_2), 2.22 (d, *J* = 16.6 Hz, 1H, CH_2), 1.08 (s, 6H, 2Me); ^{13}C NMR (100 MHz, $CDCl_3$) δ, ppm: 199.8, 193.2, 172.0, 170.5, 163.8, 136.6, 134.6, 134.2, 129.7 (2C), 129.4 (2C), 128.5 (2C), 128.1 (2C), 125.3, 107.2, 92.4, 75.9, 53.9, 51.1, 50.8, 41.6, 32.4, 29.1, 27.3; IR (NaCl, cm^{-1}): 3260, 1748, 1669, 1629, 1596; EA: Found C 68.02, H 5.19, N 3.04; calcd. for $C_{27}H_{25}NO_7$ (MW 475.50): C 68.20, H 5.30, N 2.95.

Methyl 10-benzoyl-5-hydroxy-3-(4-methoxyphenyl)-8,8-dimethyl-4,6-dioxo-4,5,6,7, 8,9-hexahydro-2,5-methanobenzo[*f*][1,3]oxazepine-2(3*H*)-carboxylate (12ab): Colourless crystals, m.p. 180-181 °C, R_f 0.65 (toluene/ethyl acetate 10:1). Yield 162 mg (0.32 mmol, 32%), ^{1}H NMR (400 MHz, $CDCl_3$) δ, ppm: 7.99 (d, *J* = 7.3 Hz, 2H, Ar), 7.63 (s, 1H, OH), 7.51 (t, *J* = 7.4 Hz, 1H, Ar), 7.38 (t, *J* = 7.7 Hz, 2H, Ar), 7.08 (d, *J* = 9.0 Hz, 2H, Ar), 6.81 (d, *J* = 9.0 Hz, 2H, Ar), 4.62 (s, 1H, CH), 3.72 (s, 3H, OMe), 3.54 (s, 3H, OMe), 2.58 (d, *J* = 17.7 Hz, 1H, CH_2), 2.42 (d, *J* = 17.7 Hz, 1H, CH_2), 2.21 (d, *J* = 16.6 Hz, 1H, CH_2), 2.14 (d, *J* = 16.7 Hz, 1H, CH_2), 1.01 (s, 3H, Me), 1.00 (s, 3H, Me); ^{13}C NMR (100 MHz, $CDCl_3$) δ, ppm: 199.6, 193.4, 172.4, 170.4, 163.8, 159.5, 136.7, 134.1, 129.7 (2C), 128.5 (2C), 127.5 (2C), 126.9, 114.7

(2C), 107.4, 92.6, 75.9, 55.6, 53.8, 51.2, 50.9, 41.6, 32.4, 29.0, 27.4; IR (NaCl, cm^{-1}): 3275, 1767, 1687, 1662, 1597; EA: Found C 66.32, H 5.49, N 2.64; Calcd. for $C_{28}H_{27}NO_8$ (MW 505.52): C 66.53, H 5.38, N 2.77.

Methyl 5-hydroxy-8,8-dimethyl-10-(4-methylbenzoyl)-4,6-dioxo-3-(4-tolyl)-4,5,6,7,8, 9-hexahydro-2,5-methanobenzo[*f*][1,3]oxazepine-2(3*H*)-carboxylate (**12ac**): Colourless crystals, m.p. 172-173 °C, R_f 0.65 (toluene/ethyl acetate 10:1). Yield 182 mg (0.36 mmol, 36%), ^{1}H NMR (400 MHz, $CDCl_3$) δ, ppm: .97 (d, *J* = 7.3 Hz, 2H, Ar), 7.75 (s, 1H, OH), 7.25 (d, *J* = 8.4 Hz, 2H, Ar), 7.19-7.13 (m, 4H, Ar), 4.67 (s, 1H, CH), 3.62 (s, 3H, OMe), 2.66 (d, *J* = 17.8 Hz, 1H, CH_2), 2.49 (d, *J* = 17.7 Hz, 1H, CH_2), 2.41 (s, 3H, C_6H_4**Me**), 2.34 (s, 3H, C_6H_4**Me**), 2.28 (d, *J* = 16.8 Hz, 1H, CH_2), 2.21 (d, *J* = 16.6 Hz, 1H, CH_2), 1.07 (s, 6H, 2Me); ^{13}C NMR (100 MHz, $CDCl_3$) δ, ppm: 199.7, 192.7, 172.2, 170.5, 163.9, 145.2, 138.2, 134.2, 131.8, 130.0 (2C), 129.9 (2C), 129.2 (2C), 125.4 (2C), 107.2, 92.5, 75.9, 53.9, 51.1, 50.8, 41.6, 32.4, 29.1, 27.4, 21.9, 21.2; IR (NaCl, cm^{-1}): 3190, 1748, 1673, 1631, 1599; EA: Found C 69.02, H 5.69, N 2.70; Calcd. for $C_{29}H_{29}NO_7$ (MW 503.55): C 69.17, H 5.81, N 2.78.

Methyl 10-benzoyl-3-(4-bromophenyl)-5-hydroxy-8,8-dimethyl-4,6-dioxo-4,5,6,7,8,9-hexahydro-2,5-methanobenzo[*f*][1,3]oxazepine-2(3*H*)-carboxylate (**12ad**): Colourless crystals, m.p. 185-186 °C, R_f 0.70 (toluene/ethyl acetate 10:1). Yield 239 mg (0.43 mmol, 43%). ^{1}H NMR (400 MHz, $CDCl_3$) δ, ppm: 8.04 (d, *J* = 7.2 Hz, 2H, Ar), 7.73 (s, 1H, OH), 7.59 (t, *J* = 7.4 Hz, 1H, Ar), 7.52-7.49 (m, 2H, Ar), 7.45 (t, *J* = 7.7 Hz, 2H, Ar), 7.21-7.18 (m, 2H, Ar), 4.66 (s, 1H, CH), 3.64 (s, 3H, OMe), 2.66 (d, *J* = 17.8 Hz, 1H, CH_2), 2.47 (d, *J* = 17.8 Hz, 1H, CH_2), 2.27 (d, *J* = 16.7 Hz, 1H, CH_2), 2.21 (d, *J* = 17.4 Hz, 1H, CH_2), 1.08 (s, 3H, Me), 1.07 (s, 3H, Me); ^{13}C NMR (100 MHz, $CDCl_3$) δ, ppm: 199.7, 193.0,

171.7, 170.4, 163.7, 136.6, 134.2, 133.7, 132.6 (2C), 129.7 (2C), 128.6 (2C), 126.6 (2C), 121.7, 107.2, 92.6, 75.9, 54.0, 51.0, 50.8, 41.6, 32.4, 29.1, 27.3; IR (NaCl, cm^{-1}): 3295, 1757, 1668, 1629, 1596; EA: Found C 58.36, H 4.29, N 2.48; Calcd. for $C_{27}H_{24}NO_7$ (MW 554.39): C 58.50, H 4.36, N 2.53.

Ethyl 5-hydroxy-8,8-dimethyl-10-(4-nitrobenzoyl)-4,6-dioxo-3-(4-tolyl)-4,5,6,7,8,9-hexahydro-2,5-methanobenzo[*f*][1,3]oxazepine-2(3*H*)-carboxylate (**12ae**): Colourless crystals, m.p. 196-198 °C, R_f 0.75 (toluene/ethyl acetate 10:1). Yield 261 mg (0.48 mmol, 48%). ^{1}H NMR (400 MHz, $CDCl_3$) δ, ppm: 8.28 (d, *J* = 8.9 Hz, 2H, Ar), 8.23 (d, *J* = 8.9 Hz, 2H, Ar), 7.78 (s, 1H, OH), 7.26-7.13 (m, 4H, Ar), 4.69 (s, 1H, CH), 4.10 (q, *J* = 7.1 Hz, 2H, OCH_2), 2.67 (d, *J* = 17.8 Hz, 1H, CH_2), 2.53 (d, *J* = 17.8 Hz, 1H, CH_2), 2.34 (s, 3H, C_6H_4**Me**), 2.29 (d, *J* = 16.7 Hz, 1H, CH_2), 2.22 (d, *J* = 16.7 Hz, 1H, CH_2), 1.09 (s, 3H, Me), 1.07 (s, 3H, Me), 0.98 (t, *J* = 7.1 Hz, 3H, CH_2**Me**); ^{13}C NMR (100 MHz, $CDCl_3$) δ, ppm: 199.7, 192.5, 171.7, 170.7, 162.9, 150.9, 141.0, 138.5, 131.6, 130.8 (2C), 130.0 (2C), 125.8 (2C), 123.7 (2C), 107.1, 92.1, 75.9, 63.6, 51.7, 50.8, 41.6, 32.4, 29.0, 27.4, 21.2, 13.7; IR (NaCl, cm^{-1}): 3239, 1749, 1676, 1630, 1600; EA: Found C 63.37, H 4.99, N 5.27; Calcd. for $C_{29}H_{28}N_2O_9$ (MW 548.55): C 63.50, H 5.15, N 5.11.

Methyl 5-hydroxy-10-(4-methylbenzoyl)-4,6-dioxo-3-(4-tolyl)-4,5,6,7,8,9-hexahydro-2,5-methanobenzo[*f*][1,3]oxazepine-2(3*H*)-carboxylate (**12bc**): Colourless crystals, m.p. 180-181 °C, R_f 0.60 (toluene/ethyl acetate 10:1). Yield 138 mg (29%), ^{1}H NMR (400 MHz, $CDCl_3$) *δ* 7.97 (d, *J* = 8.3 Hz, 2H, Ar), 7.71 (s, 1H, OH), 7.26-7.24 (m, 2H, Ar), 7.18-7.14 (m, 4H, Ar), 4.67 (s, 1H, CH), 3.61 (s, 3H, OMe), 2.81 (dt, *J* = 17.9, 5.8 Hz, 1H, CH_2), 2.58 (dt, *J* = 17.9, 6.7 Hz, 1H, CH_2), 2.40 (s, 3H, C_6H_4**Me**), 2.37 (td, *J* = 6.6, 3.8 Hz, 2H, CH_2), 2.33 (s, 3H, C_6H_4**Me**), 2.05-1.98 (m, 2H,

CH_2); ^{13}C NMR (100 MHz, $CDCl_3$) δ 199.8, 192.6, 172.2, 172.1, 163.9, 145.1, 138.1, 134.4, 131.9, 130.0 (2C), 129.8 (2C), 129.2 (2C), 125.3 (2C), 108.4, 92.5, 75.9, 53.8, 50.9, 36.9, 28.0, 21.8, 21.2, 20.2; IR (NaCl, cm^{-1}): 3275, 1753, 1667, 1630, 1595; EA: Found C 68.11, H 5.16, N 2.84; Calcd. for $C_{27}H_{25}NO_7$ (475.50): C 68.20, H 5.30, N 2.95.

Typical procedure for preparation of ketazinones 17 and 22:

5,5-Dimethyl-3-(2-(1-phenylethylidene)hydrazinyl)cyclohex-2-en-1-one (**17a**): A reaction vessel was charged with dimedone **8a** (701 mg, 5.00 mmol), acetophenone hydrazone (671 mg, 5.00 mmol), glacial acetic acid (0.25 mL), and anhydrous 1,4-dioxane (10 mL). The mixture was stirred at reflux for 30 min when yellow solid precipitate formed. The reaction mixture was cooled to room temperature and the precipitate was separated from the mother liquor by suction filtration, washed with several small portions of hexane, and recrystallized from isopropanol. The titled compound was afforded as pale-yellow crystals, m.p. 194–195 °C. Yield 730 mg (2.85 mmol, 57%). ^{1}H NMR (400 MHz, $CDCl_3$) δ, ppm: 8.19 (br. s, 1H, NH), 7.77-7.74 (m, 2H, Ph), 7.40-7.35 (m, 3H, Ph), 5.96 (s, 1H, CH), 2.51 (br. s, 2H, CH_2), 2.28 (s, 3H, Me), 2.26 (s, 2H, CH_2), 1.13 (s, 6H, 2Me); ^{13}C NMR (100 MHz, $CDCl_3$) δ, ppm: 197.3, 148.2, 138.1, 129.4, 128.6 (2C), 126.2 (2C), 99.9, 50.7, 40.4, 33.0, 28.6 (2C), 12.8; IR (NaCl, cm^{-1}): 3216, 1599; EA: Found C 74.81, H 7.97, N 10.76; Calcd. for $C_{16}H_{20}N_2O$ (MW 256.35): C 74.97, H 7.86, N 10.93.

3-(2-(1-(4-Methoxyphenyl)ethylidene)hydrazinyl)-5,5-dimethylcyclohex-2-en-1-one (**17b**): Yellow crystals, m.p. 209-210 °C. Yield 1.045 g (3.65 mmol, 73%). ^{1}H NMR (400 MHz, $CDCl_3$) δ, ppm: 7.72 (d, J = 9.0 Hz, 2H, Ar), 6.90 (d, J = 9.0

Hz, 2H, Ar), 5.92 (br. s, 1H, CH), 3.84 (s, 3H, OMe), 2.46 (br. s, 2H, CH_2), 2.26 (s, 2H, CH_2), 2.22 (s, 3H, Me), 1.13 (s, 6H, 2Me); ^{13}C NMR (100 MHz, $CDCl_3$) δ, ppm: 197.7, 160.8, 130.6, 127.6 (2C), 114.0 (2C), 99.8, 55.5, 51.0, 33.1, 28.6 (2C), 12.3; IR (NaCl, cm^{-1}): 3210, 1615, 1597; EA: Found C 71.16, H 7.63, N 9.87; Calcd. for $C_{17}H_{22}N_2O_2$ (MW 286.38): C 71.30, H 7.74, N 9.78.

3-(2-(1-(4-Methoxyphenyl)ethylidene)hydrazinyl)cyclohex-2-en-1-one (**17c**): Yellow crystals, m.p. 183-184 °C. Yield 865 mg (3.35 mmol, 67%). ^1H NMR (400 MHz, $CDCl_3$) δ, ppm: 7.71 (d, J = 8.9 Hz, 2H, Ar), 6.88 (d, J = 9.0 Hz, 2H, Ar), 6.01 (br. s, 1H, CH), 3.83 (s, 3H, OMe), 2.69 (br. s, 2H, CH_2), 2.40 (t, J = 6.5 Hz, 2H, CH_2), 2.28 (s, 3H, Me), 2.05 (dt, J = 12.6, 6.3 Hz, 2H, CH_2); ^{13}C NMR (100 MHz, DMSO-d_6) δ, ppm: 195.3, 160.0, 130.6, 127.7, 127.2 (2C), 113.7 (2C), 98.9, 55.1, 36.7, 21.5, 13.3; IR (NaCl, cm^{-1}): 3277, 1611, 1599; EA: Found C 69.58, H 6.93, N 10.97; Calcd. for $C_{15}H_{18}N_2O_2$ (MW 258.32): C 69.74, H 7.02, N 10.84.

3-(2-(Diphenylmethylene)hydrazinyl)-5,5-dimethylcyclohex-2-en-1-one (**22a**): Pale yellow crystals, m.p. 236-238 °C. Yield 1.280 g (4.05 mmol, 81%). ^1H NMR (400 MHz, $CDCl_3$) δ, ppm: 7.54-7.45 (m, 5H, Ph), 7.30-7.19 (m, 5H, Ph), 5.81 (br. s, 1H, CH), 2.44-2.11 (m, 4H, $2CH_2$), 1.02 (s, 6H, 2Me); ^{13}C NMR (100 MHz, $CDCl_3$) δ, ppm: 197.3, 137.1, 131.7, 130.1, 130.0 (2C), 129.7, 128.8 (2C), 128.5 (2C), 127.5 (2C), 100.1, 50.7, 32.9, 28.6 (2C); IR (NaCl, cm^{-1}): 3103, 1628, 1574; EA: Found C 79.04, H 6.83, N 8.95; Calcd. for $C_{21}H_{22}N_2O$ (MW 318.42): C 79.21, H 6.96, N 8.80.

3-(2-(Diphenylmethylene)hydrazinyl)cyclohex-2-en-1-one (**22b**): Pale yellow crystals, m.p. 211-212 ^oC. Yield 1.128 g (3.9 mmol, 78%). ^1H NMR (400 MHz, $CDCl_3$) δ, ppm: 7.61-7.53 (m, 5H, Ph), 7.35-7.26 (m, 5H, Ph), 5.83 (br. s, 1H, CH),

2.50 (br. s, 2H, CH_2), 2.38-2.34 (m, 2H, CH_2), 2.05-1.98 (m, 2H, CH_2); ^{13}C NMR (100 MHz, $CDCl_3$) δ, ppm: 197.8, 137.2, 131.7, 130.1, 130.0 (2C), 129.6, 128.8 (2C), 128.5 (2C), 127.4 (2C), 101.5, 37.0, 21.9; IR (NaCl, cm^{-1}): 3103, 1628, 1574; EA: Found C 78.51, H 6.18, N 9.77; Calcd. for $C_{19}H_{18}N_2O$ (MW 290.37): C 78.59, H 6.25, N 9.65.

Typical procedure for preparation of spirocyclic scaffolds 21 and 23:

3′-Benzoyl-4′-hydroxy-6,6-dimethyl-1′-phenyl-1-((1-phenylethylidene)amino)-6,7-dihydrospiro[indole-3,2′-pyrrole]-2,4,5′(1*H*,1′*H*,5*H*)-trione (**21aa**): A solution of 4-benzoyl-5-methoxycarbonyl-1-phenyl-1*H*-pyrrole-2,3-diones **9a** (168 mg, 0.50 mmol) and 5,5-dimethyl-3-(2-(1-phenylethylidene)hydrazinyl)cyclohex-2-en-1-one **17a** (128 mg, 0.50 mmol) in anhydrous toluene (4 mL) was stirred at reflux for 2 h until red colour of pyrroledione faded away, and the solid product precipitated. Then the reaction mixture was cooled to −10 °C, and the precipitate was separated by suction filtration, washed with several small portions of hexane and recrystallized from toluene/chloroform mixture (2:1). The titled compound was isolated as colourless crystals, m.p. 234-236 °C (dec.). Yield 221 mg (0.40 mmol, 79%). ^{1}H NMR (400 MHz, $CDCl_3$) δ, ppm: 7.89 (d, *J* = 7.0 Hz, 2H, Ar), 7.83 (d, *J* = 7.1 Hz, 2H, Ar), 7.54-7.47 (m, 2H, Ar), 7.45-7.38 (m, 4H, Ar), 7.35-7.28 (m, 3H, Ar), 7.18-7.14 (m, 2H, Ar), 2.48 (s, 3H, Me), 2.43 (d, *J* = 2.1 Hz, 2H, CH_2), 2.17 (d, *J* = 16.3 Hz, 1H, CH_2), 2.07 (d, *J* = 16.2 Hz, 1H, CH_2), 0.97 (s, 3H, Me), 0.69 (s, 3H, Me); ^{13}C NMR (100 MHz, $CDCl_3$) δ, ppm: 190.9, 189.8, 174.2, 169.3, 166.8, 166.1, 150.6, 137.6, 136.6, 134.6, 133.1, 131.6, 129.6 (2C), 129.4 (2C), 128.9, 128.7 (2C), 128.5 (2C), 127.7 (2C), 127.5 (2C), 117.0, 108.9, 70.1, 51.2, 36.1, 33.7, 29.4, 27.4, 18.7; IR (NaCl, cm^{-1}): 3177, 1754, 1721, 1621, 1599; EA: Found C 72.80, H 5.15, N 7.34; Calcd. for $C_{34}H_{29}N_3O_5$ (MW 559.62): C 72.97, H 5.22, N 7.51.

4′-Hydroxy-6,6-dimethyl-3′-(4-methylbenzoyl)-1-((1-phenylethylidene)amino)-1′-(4-tolyl)-6,7-dihydrospiro[indole-3,2′-pyrrole]-2,4,5′(1H,1′*H*,5*H*)-trione (**21ca**): Colourless crystals, m.p. 251-252 °C (dec.). Yield 182 mg (0.31 mmol, 62%). ^{1}H NMR (400 MHz, DMSO-d_6) δ, ppm: 7.96 (d, *J* = 7.1 Hz, 2H, Ar), 7.64 (d, *J* = 8.1 Hz, 2H, Ar), 7.59 (t, *J* = 7.3 Hz, 1H, Ar), 7.52 (t, *J* = 7.4 Hz, 2H, Ar), 7.32 (d, *J* = 8.0 Hz, 2H, Ar), 7.26 (d, *J* = 7.9 Hz, 2H, Ar), 6.95 (d, *J* = 8.3 Hz, 2H, Ar), 2.55-2.48 (m, 2H, CH_2), 2.39 (s, 3H, Me), 2.36 (s, 3H, Me), 2.32 (s, 3H, Me), 2.12 (s, 2H, CH_2), 0.90 (s, 3H, Me), 0.75 (s, 3H, Me); ^{13}C NMR (100 MHz, DMSO-d_6) δ, ppm: 190.3, 188.8, 174.7, 169.1, 165.8, 165.7, 151.9, 143.2, 138.1, 135.8, 134.7, 131.9, 131.5, 129.8 (2C), 128.9 (2C), 128.7 (2C), 128.5 (2C), 127.4 (2C), 126.9 (2C), 116.7, 108.2, 69.0, 50.5, 34.7, 33.4, 27.7, 27.1, 21.1, 20.5, 17.8; IR (NaCl, cm^{-1}): 3191, 1750, 1723, 1663, 1625, 1607; EA: Found C 73.39, H 5.64, N 6.98; Calcd. for $C_{36}H_{33}N_3O_5$ (MW 587.68): C 73.58, H 5.66, N 7.15.

3′-Benzoyl-1′-(4-bromophenyl)-4′-hydroxy-6,6-dimethyl-1-((1-phenylethylidene)amino)-6,7-dihydrospiro[indole-3,2′-pyrrole]-2,4,5′(1*H*,1′*H*,5*H*)-trione (**21da**): Colourless crystals, m.p. 245-247 °C (dec.). Yield 188 mg (0.30 mmol, 59%). ^{1}H NMR (400 MHz, DMSO-d_6) δ, ppm: 7.97 (d, *J* = 7.1 Hz, 2H, Ar), 7.71-7.69 (m, 4H, Ar), 7.64-7.57 (m, 2H, Ar), 7.54-7.49 (m, 4H, Ar), 7.00 (d, *J* = 8.7 Hz, 2H, Ar), 2.53 (s, 2H, CH_2), 2.37 (s, 3H, Me), 2.13 (s, 2H, CH_2), 0.89 (s, 3H, Me), 0.74 (s, 3H, Me); ^{13}C NMR (100 MHz, DMSO-d_6) δ, ppm: 190.6, 189.2, 175.1, 169.1, 166.3, 165.7, 152.4, 137.3, 135.7, 133.8, 132.9, 132.7 (2C), 131.7, 129.0 (2C), 128.7 (2C), 128.6 (2C), 128.3 (2C), 127.5 (2C), 121.7, 116.6, 107.9, 68.8, 50.4, 34.7, 33.5, 27.7, 27.1, 17.9; IR (NaCl, cm^{-1}): 3165, 1754, 1727, 1662, 1620, 1598; EA: Found C 64.08, H 4.31, N 6.70; Calcd. for $C_{34}H_{28}BrN_3O_5$ (MW 638.52): C 63.96, H 4.42, N 6.58.

3′-Benzoyl-4′-hydroxy-1-((1-(4-methoxyphenyl)ethylidene)amino)-6,6-dimethyl-1′-phenyl-6,7-dihydrospiro[indole-3,2′-pyrrole]-2,4,5′(1*H*,1′*H*,5*H*)-trione (**21ab**): Colourless crystals, m.p. 210-212 °C (dec.). Yield 243 mg (0.41 mmol, 82%). ^{1}H NMR (400 MHz, $CDCl_3$) δ, ppm: 7.88 (d, *J* = 9.0 Hz, 2H, Ar), 7.84 (d, *J* = 7.0 Hz, 2H, Ar), 7.53 (t, *J* = 7.4 Hz, 1H, Ar), 7.41 (t, *J* = 7.7 Hz, 2H, Ar), 7.37-7.29 (m, 3H, Ar), 7.18 (d, *J* = 8.2 Hz, 2H, Ar), 6.94 (d, *J* = 9.0 Hz, 2H, Ar), 3.86 (s, 3H, OMe), 2.43 (s, 3H, Me), 2.42 (s, 2H, CH_2), 2.17 (d, *J* = 16.3 Hz, 1H, CH_2), 2.08 (d, *J* = 16.3 Hz, 1H, CH_2), 0.97 (s, 3H, Me), 0.70 (s, 3H, Me); ^{13}C NMR (100 MHz, $CDCl_3$) δ, ppm: 190.8, 189.8, 173.6, 169.5, 167.0, 166.1, 162.6, 150.7, 137.7, 134.6, 133.1, 129.6 (2C), 129.4 (2C), 129.3 (2C), 129.0, 128.9, 128.5 (2C), 127.6 (2C), 117.0 (2C), 114.0, 108.7, 70.1, 55.6, 51.3, 36.1, 33.7, 29.4, 27.4, 18.2; IR (NaCl, cm^{-1}): 3173, 1748, 1722, 1628, 1596; EA: Found C 71.13, H 5.39, N 7.04; Calcd. for $C_{35}H_{31}N_3O_6$ (MW 589.65): C 71.29, H 5.30, N 7.13.

4′-Hydroxy-1-((1-(4-methoxyphenyl)ethylidene)amino)-6,6-dimethyl-3′-(4-nitrobenzoyl)-1′-(4-tolyl)-6,7-dihydrospiro[indole-3,2'-pyrrole]-2,4,5′(1*H*,1′*H*,5*H*)-trione (**21eb**): Colourless crystals, m.p. 261-263 °C (dec.). Yield 245 mg (0.38 mmol, 76%). ^{1}H NMR (400 MHz, DMSO-d_6) δ, ppm: 8.34 (d, *J* = 8.9 Hz, 2H, Ar), 7.92 (d, *J* = 9.0 Hz, 2H, Ar), 7.87 (d, *J* = 8.9 Hz, 2H, Ar), 7.26 (d, *J* = 8.1 Hz, 2H, Ar), 7.04 (d, *J* = 9.0 Hz, 2H, Ar), 6.94 (d, *J* = 8.3 Hz, 2H, Ar), 3.84 (s, 3H, OMe), 2.53-2.41 (m, 2H, CH_2), 2.32 (s, 3H, Me), 2.24 (s, 3H, C_6H_4**Me**), 2.14 (s, 2H, CH_2), 0.92 (s, 3H, Me), 0.77 (s, 3H, Me); ^{13}C NMR (100 MHz, DMSO-d_6) δ, ppm: 190.4, 187.5, 174.4, 169.1, 166.1, 165.3, 162.1, 154.9, 149.5, 142.8, 138.4, 131.7, 129.9 (2C), 129.7 (2C), 129.3 (2C), 127.9, 126.9 (2C), 123.5 (2C), 115.3, 113.9 (2C), 108.0, 68.6, 55.4,

50.5, 34.7, 33.4, 27.7, 27.2, 20.6, 17.2; IR (NaCl, cm^{-1}): 3424, 1753, 1724, 1626, 1602; EA: Found C 66.47, H 5.05, N 8.48; Calcd. for $C_{36}H_{32}N_4O_8$ (MW 648.67): C 66.66, H 4.97, N 8.64.

4′-Hydroxy-1-((1-(4-methoxyphenyl)ethylidene)amino)-6,6-dimethyl-3′-(4-methylbenzoyl)-1′-phenyl-6,7-dihydrospiro[indole-3,2′-pyrrole]-2,4,5′(1*H*,1′*H*,5*H*)-trione (**21fb**): colourless crystals, m.p. 228-230 °C (dec.). Yield 250 mg (0.42 mmol, 83%). ^{1}H NMR (400 MHz, $CDCl_3$) δ, ppm: 7.89 (d, *J* = 9.0 Hz, 2H, Ar), 7.77 (d, *J* = 8.1 Hz, 2H, Ar), 7.37-7.29 (m, 3H, Ar), 7.21 (d, *J* = 7.9 Hz, 2H, Ar), 7.22-7.16 (m, 2H, Ar), 6.94 (d, *J* = 9.0 Hz, 2H, Ar), 3.86 (s, 3H, OMe), 2.45 (s, 3H, Me), 2.43 (s, 2H, CH_2), 2.39 (s, 3H, C_6H_4**Me**), 2.17 (d, *J* = 16.2 Hz, 1H, CH_2), 2.07 (d, *J* = 16.2 Hz, 1H, CH_2), 0.97 (s, 3H, Me), 0.69 (s, 3H, Me); ^{13}C NMR (100 MHz, $CDCl_3$) δ, ppm: 190.9, 189.4, 173.5, 169.5, 167.0, 166.2, 162.5, 150.1, 144.1, 134.9, 134.6, 129.6 (2C), 129.5 (2C), 129.4 (2C), 129.2 (2C), 129.0, 128.8, 127.5 (2C), 117.3, 113.9 (2C), 108.6, 70.1, 55.6, 51.2, 36.0, 33.7, 29.4, 27.3, 21.9, 18.2; IR (NaCl, cm^{-1}): 3158, 1739, 1717, 1650, 1630, 1604; EA: Found C 71.48, H 5.43, N 7.11; Calcd. for $C_{36}H_{33}N_3O_6$ (603.68): C 71.63, H 5.51, N 6.96.

3′-Benzoyl-1′-(4-chlorophenyl)-4′-hydroxy-1-((1-(4-methoxyphenyl)ethylidene)amino)-6,6-dimethyl-6,7-dihydrospiro[indole-3,2′-pyrrole]-2,4,5′(1*H*,1′*H*,5*H*)-trione (**21gb**): Colourless crystals, m.p. 214-216 °C (dec.). Yield 237 mg (0.38 mmol, 76%). ^{1}H NMR (400 MHz, $CDCl_3$) δ, ppm: 7.89 (d, *J* = 9.0 Hz, 2H, Ar), 7.82 (d, *J* = 7.0 Hz, 2H, Ar), 7.54 (t, *J* = 7.4 Hz, 1H, Ar), 7.41 (t, *J* = 7.7 Hz, 2H, Ar), 7.31 (d, *J* = 8.7 Hz, 2H, Ar), 7.11 (d, *J* = 8.6 Hz, 2H, Ar), 6.95 (d, *J* = 9.0 Hz, 2H, Ar), 3.87 (s, 3H, OMe), 2.51-2.47 (m, 4H, Me, CH_2), 2.41 (d, *J* = 18.8 Hz, 1H, CH_2), 2.18 (d, *J* = 16.3 Hz, 1H, CH_2), 2.09 (d, *J* = 16.3 Hz, 1H, CH_2), 0.98 (s, 3H, Me), 0.77 (s, 3H, Me); ^{13}C NMR (100 MHz, $CDCl_3$) δ, ppm: 191.0,

189.7, 173.9, 169.5, 167.2, 166.1, 162.7, 150.3, 137.6, 134.8, 133.2 (2C), 129.8 (2C), 129.5 (2C), 129.4 (2C), 129.0 (2C), 128.9, 128.5 (2C), 117.2, 114.1 (2C), 108.5, 70.0, 55.6, 51.3, 36.1, 33.9, 29.3, 27.6, 18.2; IR (NaCl, cm^{-1}): 3449, 1748, 1732, 1631, 1597; EA: Found C 67.28, H 4.78, N 6.81; Calcd. for $C_{35}H_{30}ClN_3O_6$ (MW 624.09): C 67.36, H 4.85, N 6.73.

3′-Benzoyl-4′-hydroxy-1′-(4-methoxyphenyl)-1-((1-(4-methoxyphenyl)ethylidene)amino)-6,7-dihydrospiro[indole-3,2′-pyrrole]-2,4,5′(1*H*,1′*H*,5*H*)-trione (**21bc**): Colourless crystals, m.p. 243-245 °C (dec.). Yield 207 mg (0.35 mmol, 70%). ^{1}H NMR (400 MHz, $CDCl_3$) δ, ppm: 7.87-7.82 (m, 4H, Ar), 7.54 (t, *J* = 7.4 Hz, 1H, Ar), 7.43 (t, *J* = 7.6 Hz, 2H, Ar), 7.08 (d, *J* = 9.0 Hz, 2H, Ar), 6.92 (d, *J* = 9.0 Hz, 2H, Ar), 6.86 (d, *J* = 9.0 Hz, 2H, Ar), 3.86 (s, 3H, OMe), 3.80 (s, 3H, OMe), 2.61-2.52 (m, 2H, CH_2), 2.38 (s, 3H, Me), 2.29 (t, *J* = 6.6 Hz, 2H, CH_2), 2.05-1.96 (m, 1H, CH_2), 1.93-1.84 (m, 1H, CH_2); ^{13}C NMR (100 MHz, $CDCl_3$) δ, ppm: 191.3, 189.6, 173.4, 169.1, 168.2, 166.2, 162.5, 160.0, 151.0, 137.6, 133.1, 129.4 (4C), 129.3 (2C), 129.0, 128.5 (2C), 126.8, 116.8, 114.8 (2C), 114.0 (2C), 109.9, 70.3, 55.6 (2C), 37.2, 22.6, 21.4, 18.2; IR (NaCl, cm^{-1}): 3374, 1754, 1721, 1663, 1623, 1594; EA: Found C 68.95, H 4.86, N 7.16; Calcd. for $C_{34}H_{29}N_3O_7$ (MW 591.62): C 69.03, H 4.94, N 7.10.

3′-Benzoyl-4-((diphenylmethylene)hydrazono)-4′-hydroxy-6,6-dimethyl-1′-phenyl-4,5,6,7-tetrahydro-2*H*-spiro[benzofuran-3,2′-pyrrole]-2,5′(1'*H*)-dione (**23aa**): Yellow crystals, m.p. 221-222 °C. Yield 242 mg (0.39 mmol, 78%). ^{1}H NMR (400 MHz, $CDCl_3$) δ, ppm: 7.62 (d, *J* = 7.0 Hz, 2H, Ar), 7.52-7.46 (m, 5H, Ar), 7.42-7.31 (m, 11H, Ar), 7.09-7.07 (m, 2H, Ar), 2.83 (d, *J* = 16.6 Hz, 1H, CH_2), 2.56 (d, *J* = 16.6 Hz, 1H, CH_2), 2.41 (d, *J* = 18.3 Hz, 1H, CH_2), 2.19 (d, *J* = 18.3 Hz, 1H, CH_2), 1.01 (s, 3H, Me), 0.72 (s, 3H, Me); ^{13}C NMR (100 MHz, $CDCl_3$) δ, ppm: 188.7, 172.6, 165.5, 165.3,

165.1, 159.7, 149.3, 138.8, 136.9, 136.5, 134.2, 133.2, 130.5, 129.7 (2C), 129.5 (2C), 129.3 (2C), 129.1 (3C), 128.8, 128.4 (2C), 128.3 (4C), 127.4 (2C), 117.3, 110.6, 70.2, 38.9, 37.0, 32.5, 29.4, 27.8; IR (NaCl, cm^{-1}): 3298, 1833, 1708, 1659, 1626, 1574; EA: Found C 75.23, H 4.99, N 6.84; Calcd. for $C_{39}H_{31}N_3O_5$ (MW 621.69): C 75.35, H 5.03, N 6.76.

3′-Benzoyl-4-((diphenylmethylene)hydrazono)-4′-hydroxy-1′-(4-methoxyphenyl)-6,6-dimethyl-4,5,6,7-tetrahydro-2*H*-spiro[benzofuran-3,2′-pyrrole]-2,5′(1′*H*)-dione (**23ba**): Yellow crystals, m.p. 230-231 °C. Yield 245 mg (0.38 mmol, 75%). ^{1}H NMR (400 MHz, $CDCl_3$) δ, ppm: 7.62 (d, *J* = 7.0 Hz, 2H, Ar), 7.52-7.45 (m, 5H, Ar), 7.42-7.30 (m, 8H, Ar), 6.99 (d, *J* = 8.9 Hz, 2H, Ar), 6.83 (d, *J* = 9.0 Hz, 2H, Ar), 3.78 (s, 3H, OMe), 2.81 (d, *J* = 16.6 Hz, 1H, CH_2), 2.61 (d, *J* = 16.5 Hz, 1H, CH_2), 2.40 (d, *J* = 18.3 Hz, 1H, CH_2), 2.21 (d, *J* = 18.3 Hz, 1H, CH_2), 1.01 (s, 3H, Me), 0.81 (s, 3H, Me); ^{13}C NMR (100 MHz, $CDCl_3$) δ, ppm: 188.7, 172.6, 165.7, 165.2, 165.1, 160.2, 159.7, 149.5, 138.8, 137.0, 136.4, 133.1, 130.5, 129.5 (2C), 129.3 (2C), 129.1 (4C), 128.8, 128.4 (2C), 128.3 (4C), 126.5, 117.2, 115.0 (2C), 110.6, 70.4, 55.6, 38.9, 37.0, 32.5, 29.3, 28.1; IR (NaCl, cm^{-1}): 3286, 1810, 1704, 1662, 1634, 1574; EA: Found C 73.58, H 5.02, N 6.56; Calcd. for $C_{40}H_{33}N_3O_6$ (MW 651.72): C 73.72, H 5.10, N 6.45.

3′-Benzoyl-1′-(4-bromophenyl)-4-((diphenylmethylene)hydrazono)-4′-hydroxy-6,6-dimethyl-4,5,6,7-tetrahydro-2*H*-spiro[benzofuran-3,2′-pyrrole]-2,5′(1′*H*)-dione (**23da**): Yellow crystals, m.p. 225-226 °C. Yield 224 mg (0.32 mmol, 64%). ^{1}H NMR (400 MHz, $CDCl_3$) δ, ppm: 7.61 (d, *J* = 7.0 Hz, 2H, Ar), 7.54-7.44 (m, 7H, Ar), 7.42-7.32 (m, 6H, Ar), 7.28 (d, *J* = 6.8 Hz, 2H, Ar), 6.91 (d, *J* = 8.7 Hz, 2H, Ar), 2.77 (d, *J* = 16.5 Hz, 1H, CH_2), 2.60 (d, *J* = 16.5 Hz, 1H, CH_2), 2.42 (d, *J* = 18.4 Hz, 1H, CH_2), 2.24 (d, *J* = 18.4 Hz, 1H, CH_2), 1.01 (s, 3H, Me), 0.80 (s, 3H, Me); ^{13}C NMR (100 MHz, $CDCl_3$) δ,

ppm: 188.6, 172.4, 165.4 (2C), 165.0, 159.2, 149.1, 138.6, 136.8, 136.5, 133.3, 133.2, 132.9 (2C), 130.6, 129.5 (2C), 129.1 (2C), 129.0 (4C), 128.8, 128.4 (2C), 128.3 (4C), 123.2, 117.5, 110.3, 70.0, 38.9, 37.0, 32.5, 29.2, 28.1; IR (NaCl, cm^{-1}): 3107, 1828, 1712, 1662, 1625, 1597; EA: Found C 66.78, H 4.24, N 5.91; Calcd. for $C_{39}H_{30}BrN_3O_5$ (MW 700.59): C 66.86, H 4.32, N 6.00.

4-((Diphenylmethylene)hydrazono)-4′-hydroxy-6,6-dimethyl-3′-(4-nitrobenzoyl)-1′-(4-tolyl)-4,5,6,7-tetrahydro-2*H*-spiro[benzofuran-3,2′-pyrrole]-2,5′(1′*H*)-dione (**23ea**): Yellow crystals, m.p. 260-262 °C (dec.). Yield 208 mg (0.31 mmol, 61%). ^{1}H NMR (400 MHz, DMSO-d_6) δ, ppm: 8.20 (d, *J* = 8.9 Hz, 2H, Ar), 7.58-7.51 (m, 6H, Ar), 7.46-7.39 (m, 4H, Ar), 7.32 (d, *J* = 6.9 Hz, 2H, Ar), 7.22 (d, *J* = 8.0 Hz, 2H, Ar), 6.79 (d, *J* = 8.3 Hz, 2H, Ar), 2.64 (d, *J* = 16.5 Hz, 1H, CH_2), 2.57 (d, *J* = 16.5 Hz, 1H, CH_2), 2.42 (d, *J* = 18.4 Hz, 1H, CH_2), 2.30 (d, *J* = 18.3 Hz, 1H, CH_2), 2.30 (s, 3H, C_6H_4**Me**), 0.93 (s, 3H, Me), 0.73 (s, 3H, Me); ^{13}C NMR (100 MHz, DMSO-d_6) δ, ppm: 186.6, 173.4, 164.7, 164.3, 162.7, 158.0, 154.7, 149.5, 141.8, 138.2, 138.0, 135.0, 131.5, 130.1, 129.9 (4C), 128.8, 128.6 (2C), 128.3 (2C), 128.2 (2C), 128.1 (2C), 126.6 (2C), 123.2 (2C), 115.0, 110.1, 68.6, 38.1, 35.7, 32.0, 27.9, 27.3, 20.5; IR (NaCl, cm^{-1}): 3343, 1828, 1702, 1661, 1635, 1592; EA: Found C 70.39, H 4.66, N 8.12; Calcd. for $C_{40}H_{32}N_4O_7$ (MW 680.72): C 70.58, H 4.74, N 8.23.

3′-Benzoyl-1′-(4-chlorophenyl)-4-((diphenylmethylene)hydrazono)-4′-hydroxy-6,6-dimethyl-4,5,6,7-tetrahydro-2*H*-spiro[benzofuran-3,2′-pyrrole]-2,5′(1′*H*)-dione (**23ga**): Yellow crystals, m.p. 226-227 °C. Yield 194 mg (0.30 mmol, 59%). ^{1}H NMR (400 MHz, $CDCl_3$) δ, ppm: 7.61 (d, *J* = 7.1 Hz, 2H, Ar), 7.54-7.45 (m, 5H, Ar), 7.42-7.27 (m, 10H, Ar), 6.97

(d, *J* = 8.7 Hz, 2H, Ar), 2.77 (d, *J* = 16.6 Hz, 1H, CH_2), 2.59 (d, *J* = 16.5 Hz, 1H, CH_2), 2.42 (d, *J* = 18.4 Hz, 1H, CH_2), 2.23 (d, *J* = 18.4 Hz, 1H, CH_2), 1.01 (s, 3H, Me), 0.80 (s, 3H, Me); ^{13}C NMR (100 MHz, $CDCl_3$) δ, ppm: 188.6, 172.4, 165.5, 165.4, 165.0, 159.2, 149.2, 138.6, 136.8, 136.5, 135.2, 133.3, 132.7, 130.6, 129.9 (2C), 129.5 (2C), 129.1 (2C), 129.0 (2C), 128.8 (3C), 128.4 (2C), 128.3 (4C), 117.6, 110.3, 70.1, 38.9, 37.0, 32.5, 29.2, 28.1; IR (NaCl, cm^{-1}): 3306, 1807, 1710, 1666, 1633, 1587; EA: Found C 71.26, H 4.53, N 6.51; Calcd. for $C_{39}H_{30}ClN_3O_5$ (MW 656.14): C 71.39, H 4.61, N 6.40.

3′-(4-Bromobenzoyl)-4-((diphenylmethylene)hydrazono)-4′-hydroxy-6,6-dimethyl-1′-(4-tolyl)-4,5,6,7-tetrahydro-2*H*-spiro[benzofuran-3,2′-pyrrole]-2,5′(1′*H*)-dione (**23ha**): Yellow crystals, m.p. 230-232 °C (dec.). Yield 203 mg (0.29 mmol, 57%). ^{1}H NMR (400 MHz, $CDCl_3$) δ, ppm: 7.61 (d, *J* = 7.1 Hz, 2H, Ar), 7.55-7.45 (m, 4H, Ar), 7.41-7.28 (m, 7H, Ar), 7.25-7.12 (m, 3H, Ar), 6.95 (d, *J* = 6.0 Hz, 2H, Ar), 2.77 (d, *J* = 16.6 Hz, 1H, CH_2), 2.65 (d, *J* = 16.6 Hz, 1H, CH_2), 2.41 (d, *J* = 18.3 Hz, 1H, CH_2), 2.33 (s, 3H, C_6H_4**Me**), 2.22 (d, *J* = 18.4 Hz, 1H, CH_2), 1.01 (s, 3H, Me), 0.80 (s, 3H, Me); ^{13}C NMR (100 MHz, DMSO-d_6) δ, ppm: 187.3, 173.3, 164.7, 164.4, 163.0, 158.4, 152.7, 138.2, 138.0, 135.5, 134.9, 131.5, 131.2 (2C), 130.8 (2C), 130.1, 129.9 (2C), 128.8, 128.6 (2C), 128.3 (2C), 128.2 (2C), 128.0 (2C), 126.9, 126.5 (2C), 115.8, 110.0, 68.7, 38.0, 35.7, 32.0, 27.9, 27.2, 20.5; IR (NaCl, cm^{-1}): 3084, 1831, 1705, 1667, 1636, 1587; EA: Found C 67.09, H 4.36, N 6.01; Calcd. for $C_{40}H_{32}BrN_3O_5$ (MW 714.62): C 67.23, H 4.51, N 5.88.

3′-Benzoyl-4-((diphenylmethylene)hydrazono)-4′-hydroxy-1′-(4-methoxyphenyl)-4,5,6,7-tetrahydro-2*H*-spiro[benzofuran-3,2′-pyrrole]-2,5′(1′*H*)-dione (**23bb**): Yellow crystals, m.p. 225-226 °C (dec.). Yield 206 mg (0.33

mmol, 66%). ^{1}H NMR (400 MHz, $CDCl_3$) δ, ppm: 7.62 (d, *J* = 7.1 Hz, 2H, Ar), 7.51-7.44 (m, 5H, Ar), 7.42-7.29 (m, 8H, Ar), 6.98 (d, *J* = 9.0 Hz, 2H, Ar), 6.84 (d, *J* = 9.0 Hz, 2H, Ar), 3.79 (s, 3H, OMe), 2.96-2.81 (m, 2H, CH_2), 2.53 (dt, *J* = 18.5, 6.3 Hz, 1H, CH_2), 2.38 (dt, *J* = 12.3, 5.8 Hz, 1H, CH_2), 1.97-1.87 (m, 1H, CH_2), 1.85-1.75 (m, 1H, CH_2); ^{13}C NMR (100 MHz, $CDCl_3$) δ, ppm: 188.6, 172.3, 166.2, 165.7, 165.1, 160.2, 160.1, 149.7, 138.8, 136.9, 136.3, 133.1, 130.5, 129.6 (2C), 129.3 (2C), 129.1 (4C), 128.8, 128.4 (2C), 128.3 (4C), 126.3, 117.2, 115.0 (2C), 111.6, 70.3, 55.6, 25.1, 23.3, 20.8; IR (NaCl, cm^{-1}): 3167, 1820, 1713, 1661, 1632, 1586; EA: Found C 73.04, H 4.61, N 6.86; Calcd. for $C_{38}H_{29}N_3O_6$ (MW 623.67): C 73.18, H 4.69, N 6.74.

References

[S1] Tsuda, Y.; Horiguchi, Y.; Sano, T. *Heterocycles* **1976**, *7*, 1237-1242.

[S2[Andreichikov, Yu. S.; Maslivets, A. N.; Smirnova, L. I.; Krasnikh, O. P.; Kozlov, A. P.; Perevozchikov, L. A. *Russ. J. Org. Chem*. **1987**, *23*, 1534-1543.

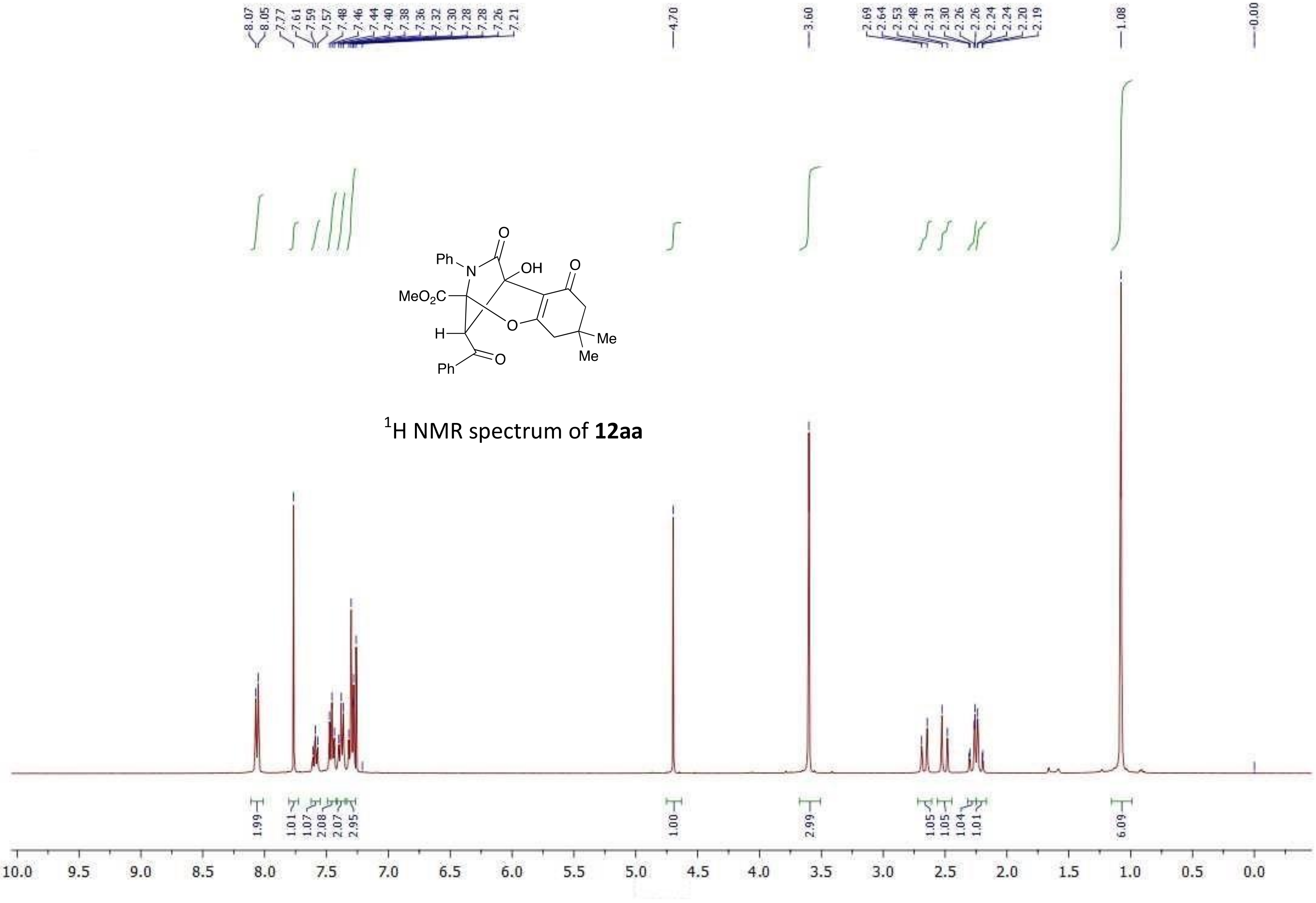

^{1}H NMR spectrum of **12aa**

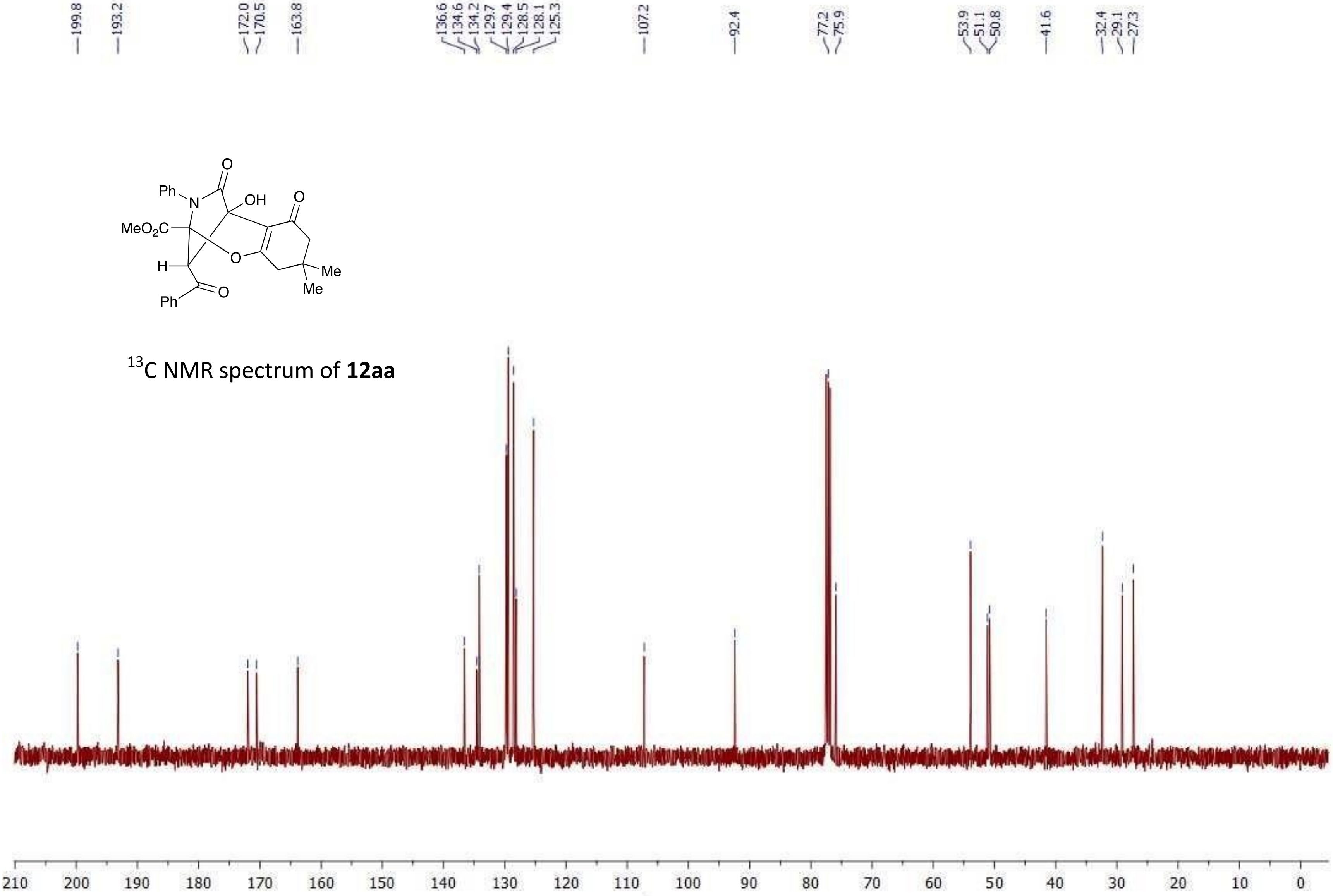

^{13}C NMR spectrum of **12aa**

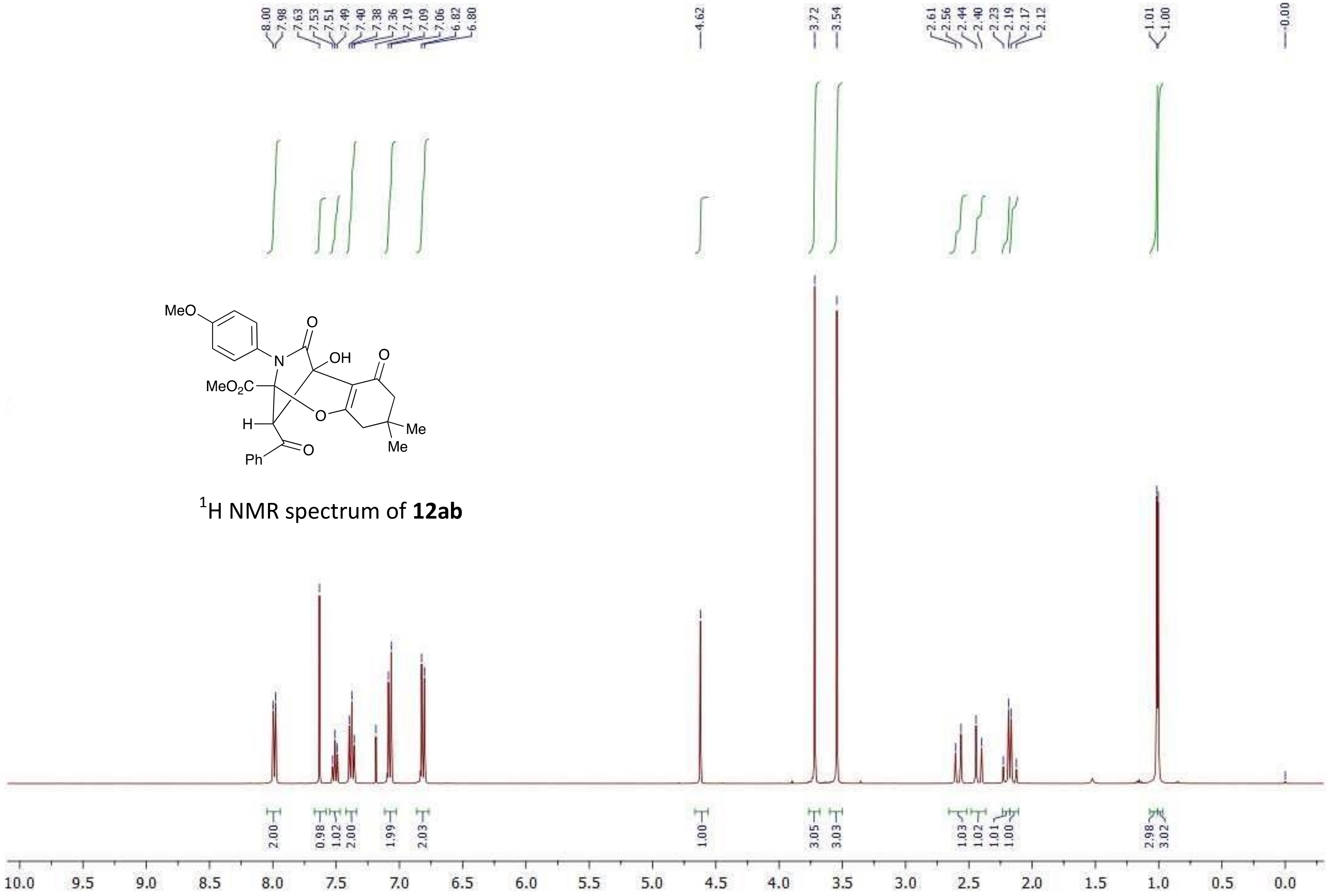

^{1}H NMR spectrum of **12ab**

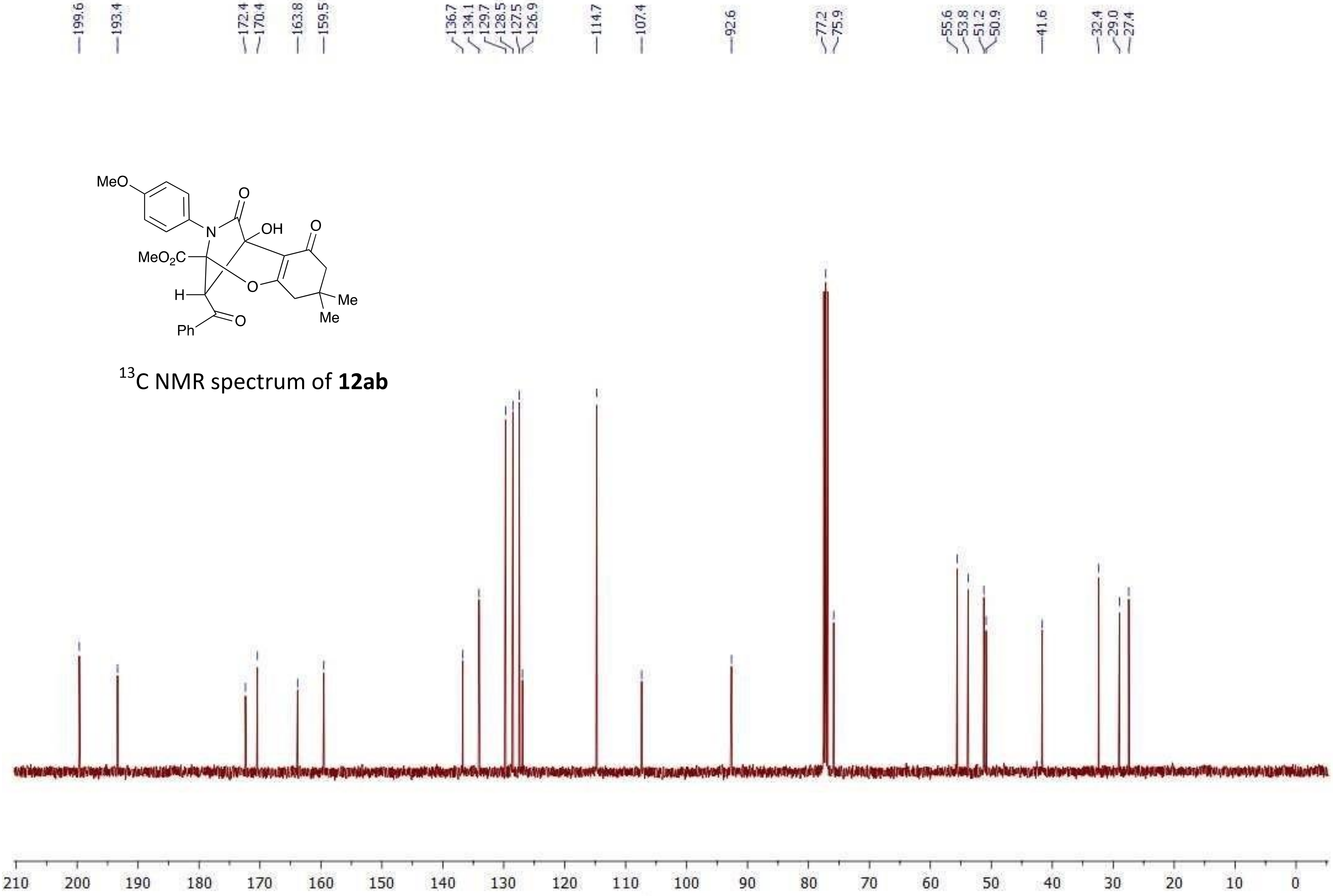

^{13}C NMR spectrum of **12ab**

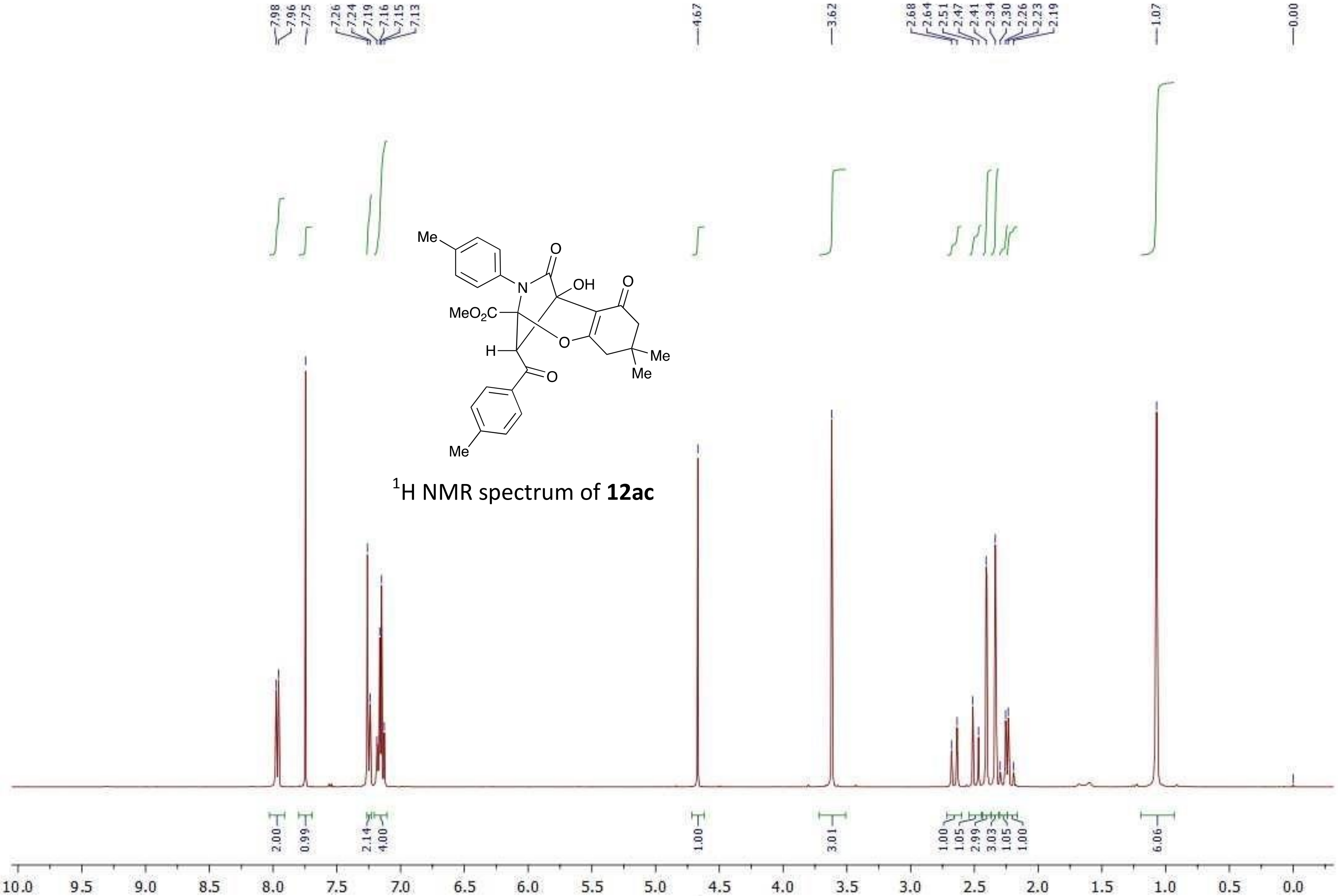

^{1}H NMR spectrum of **12ac**

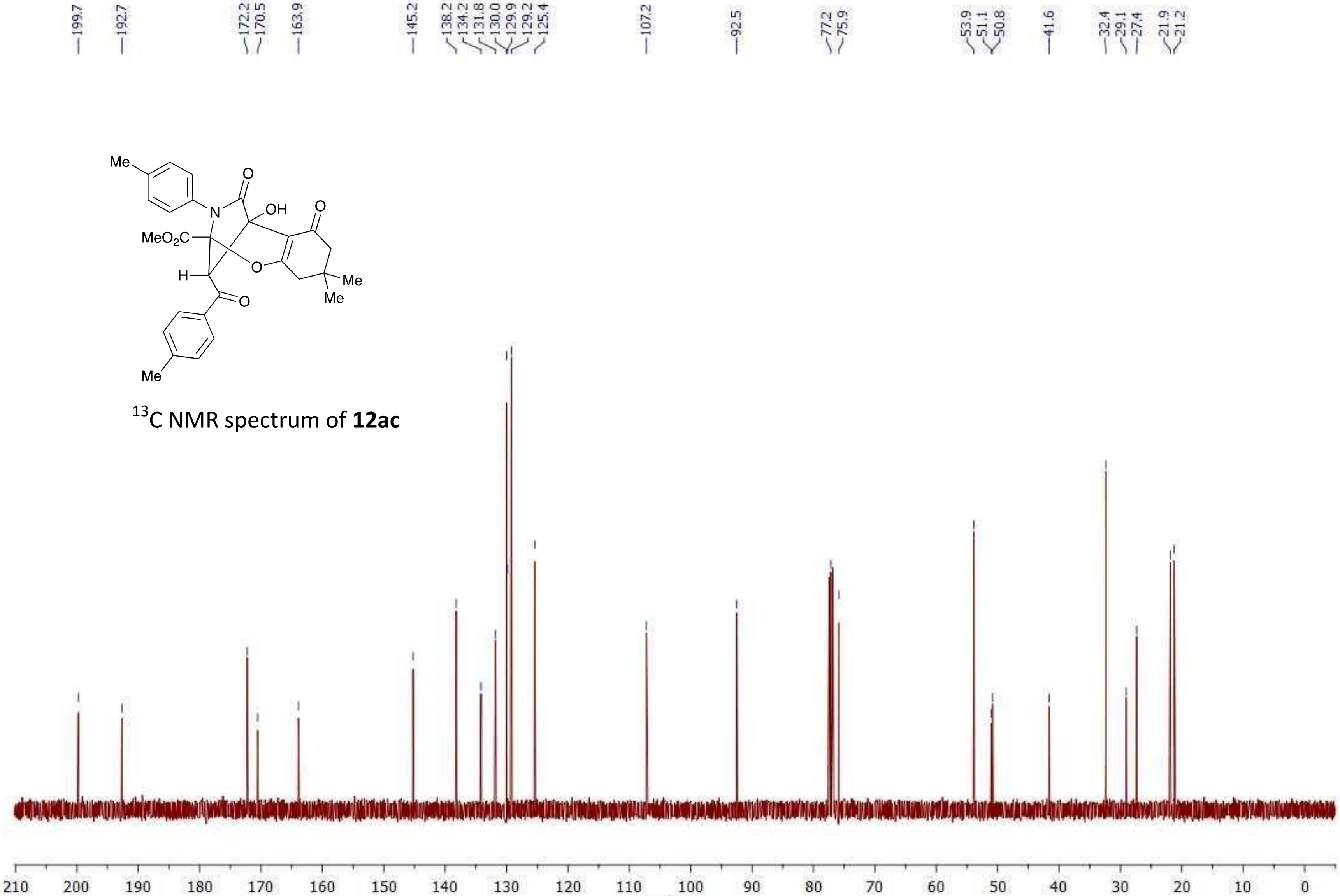

^{13}C NMR spectrum of **12ac**

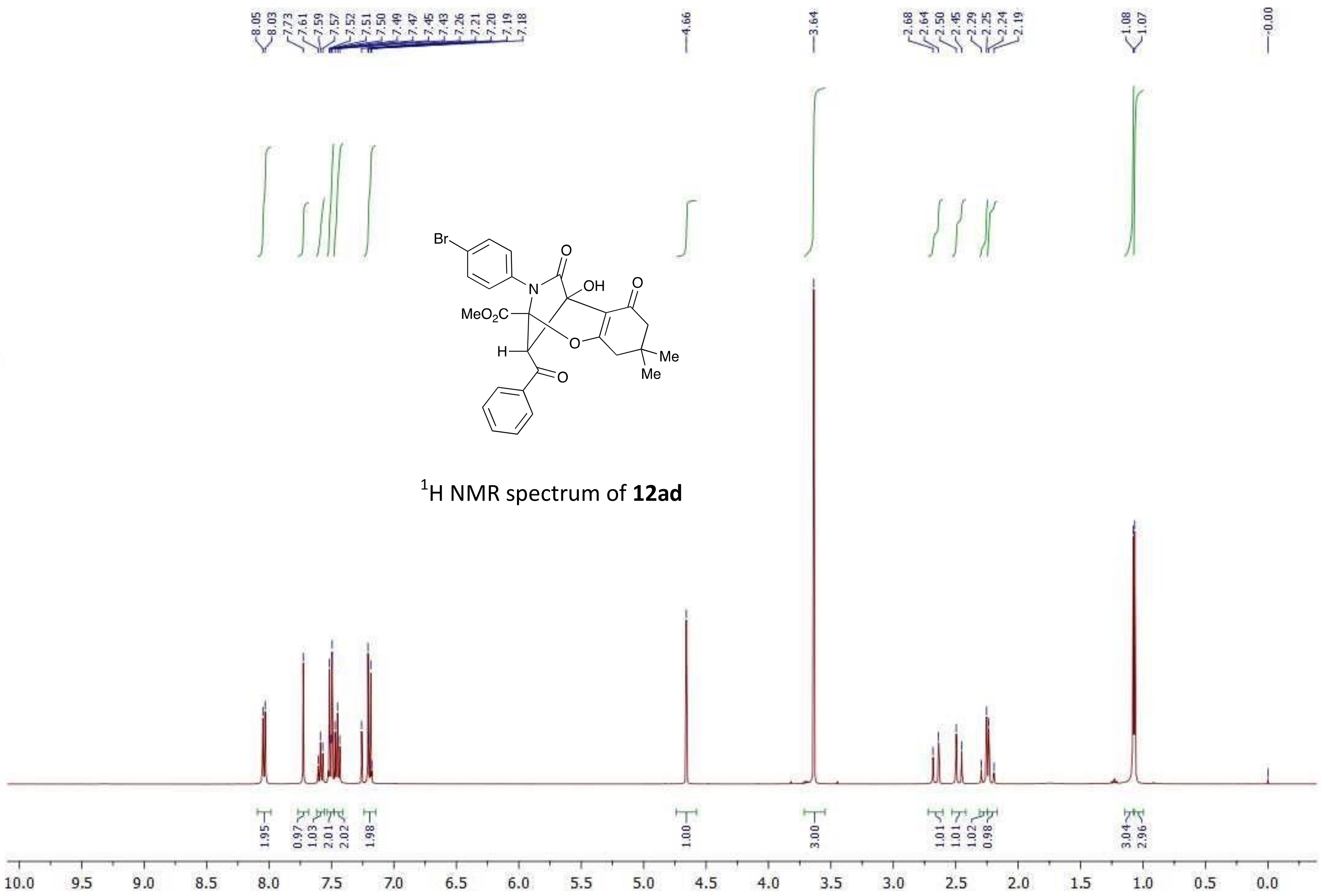

^{1}H NMR spectrum of **12ad**

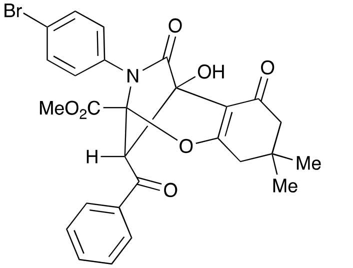

^{13}C NMR spectrum of **12ad**

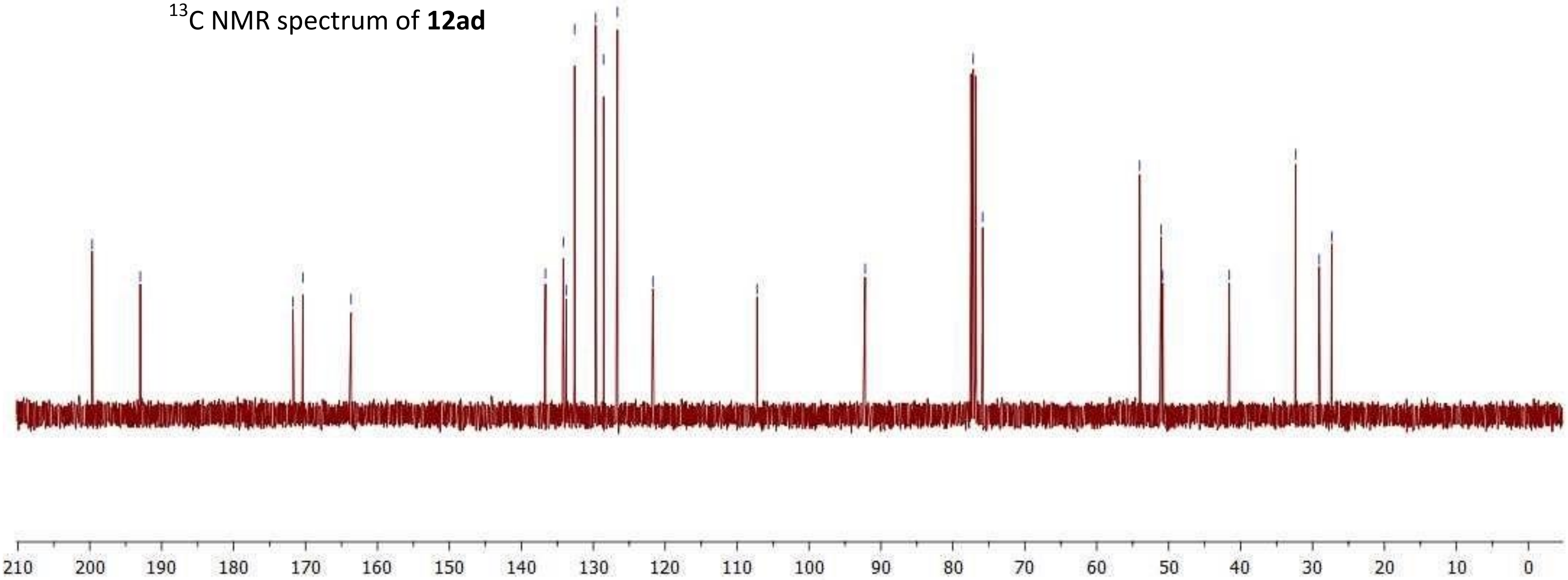

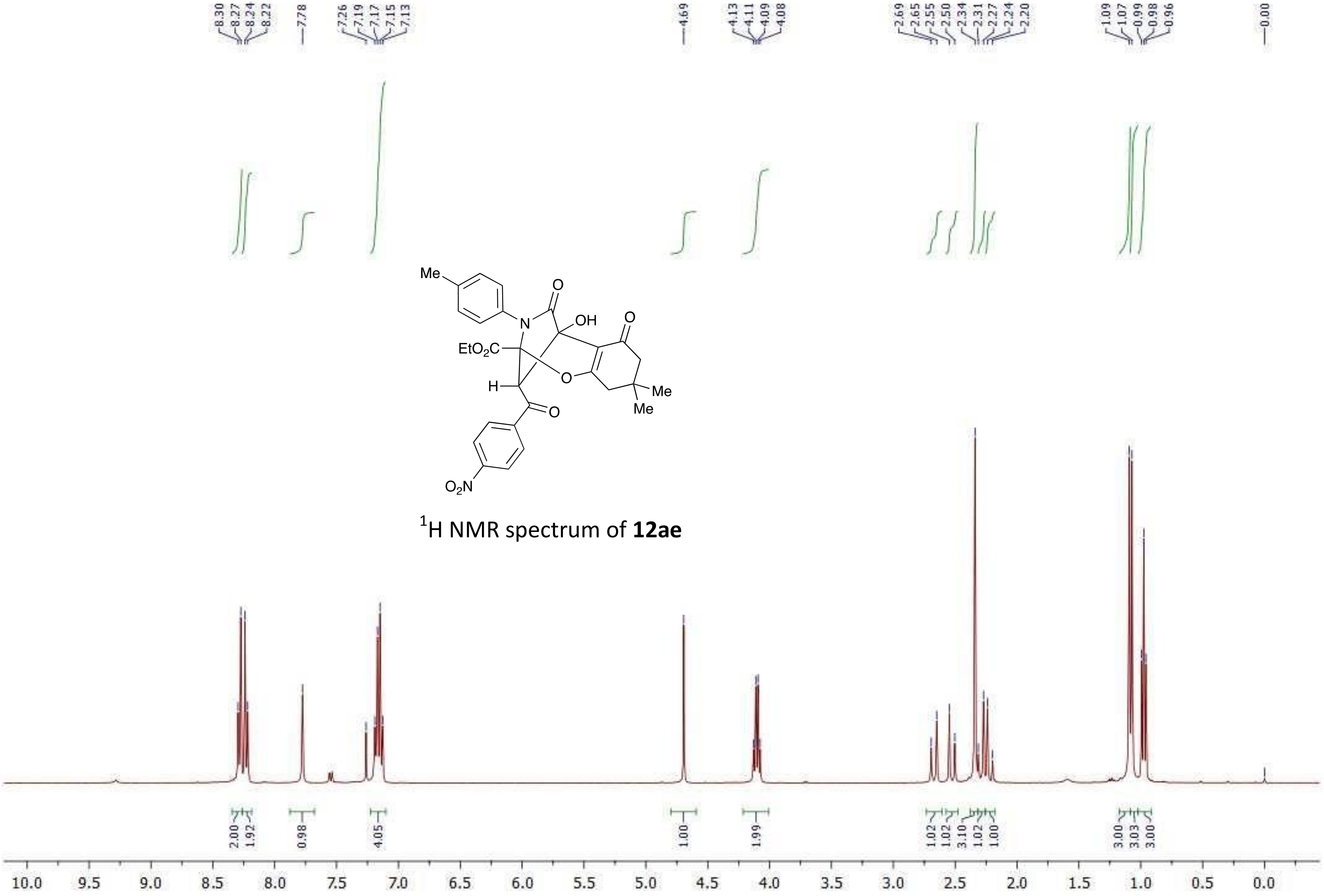

^{1}H NMR spectrum of **12ae**

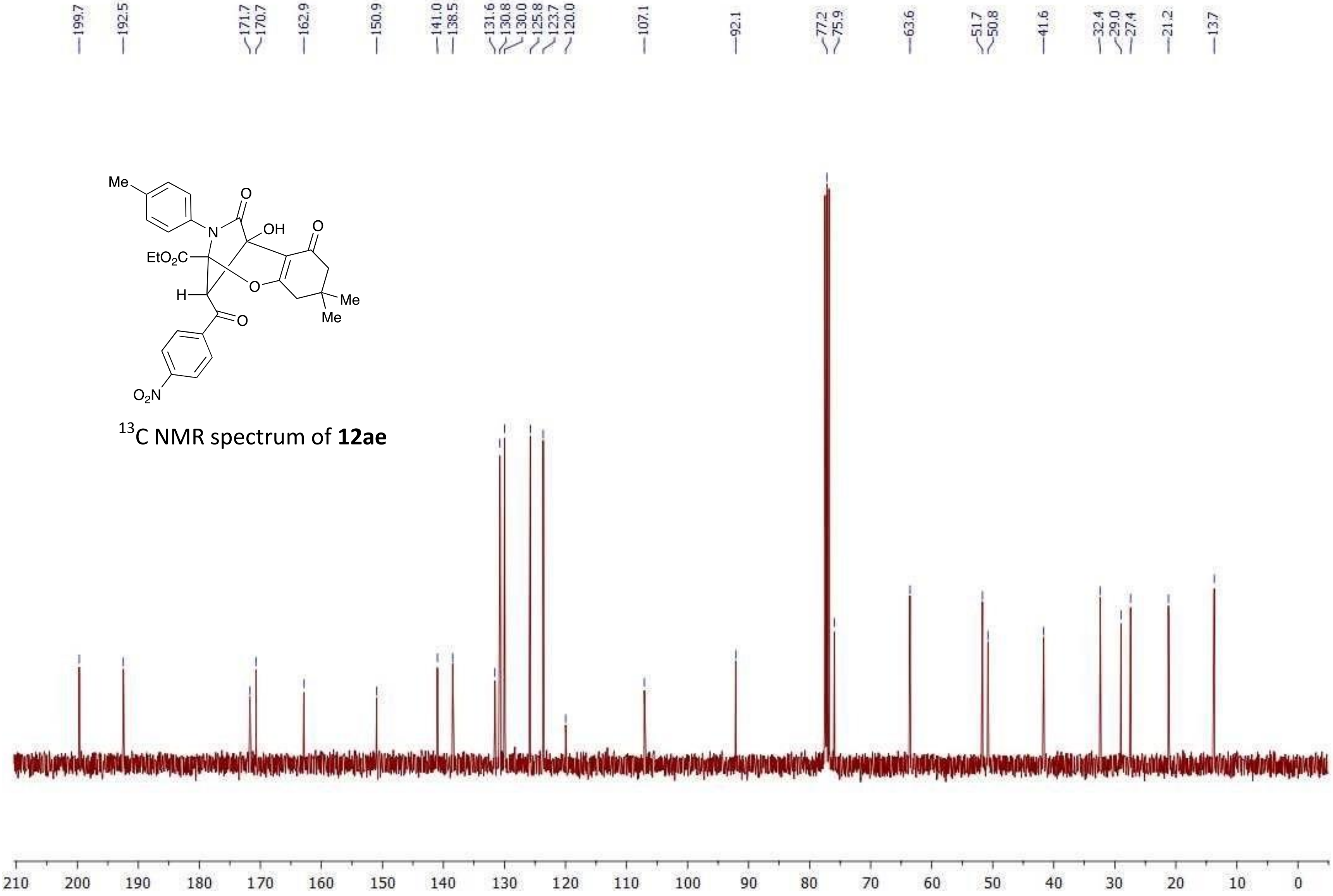

^{13}C NMR spectrum of **12ae**

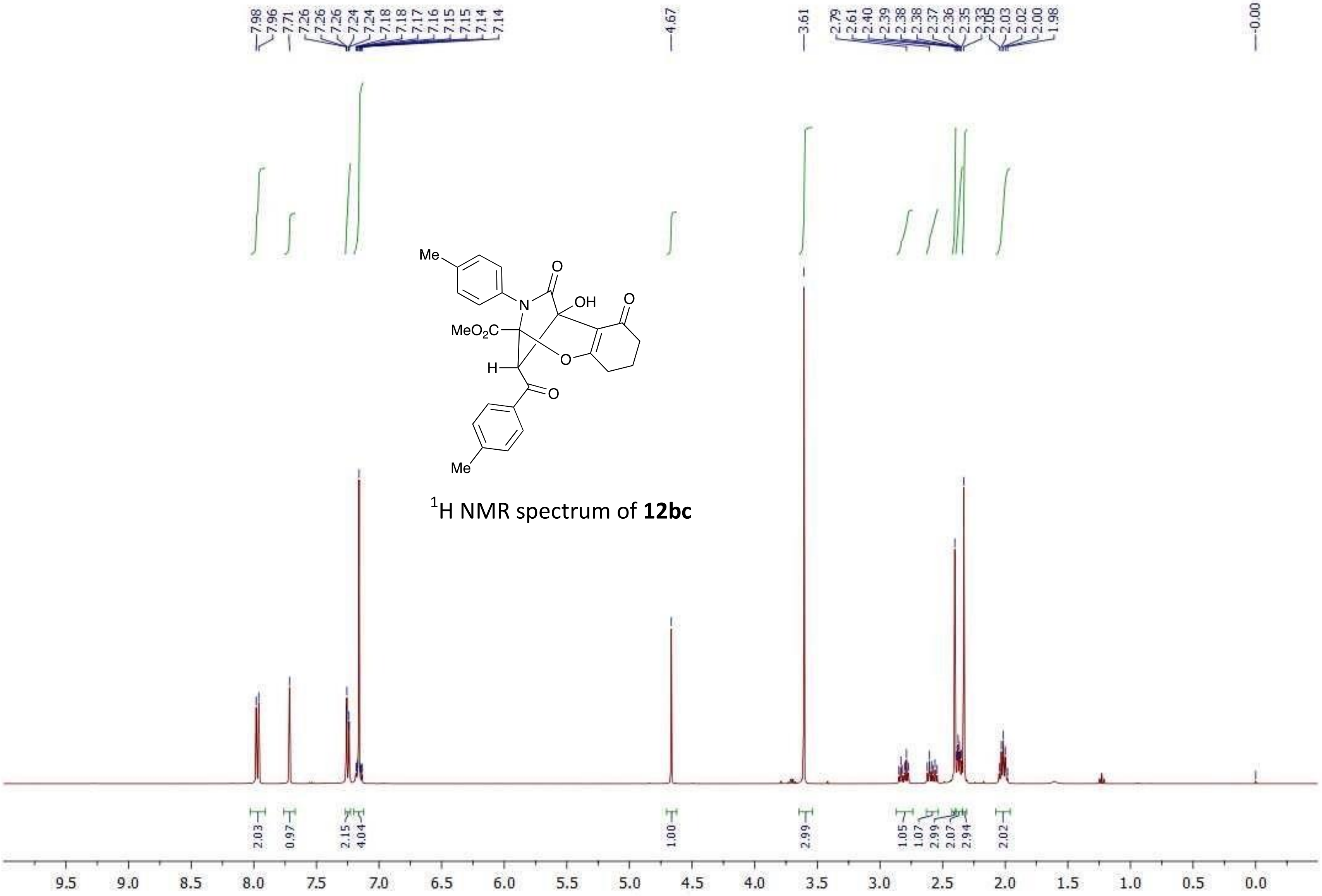

^{1}H NMR spectrum of **12bc**

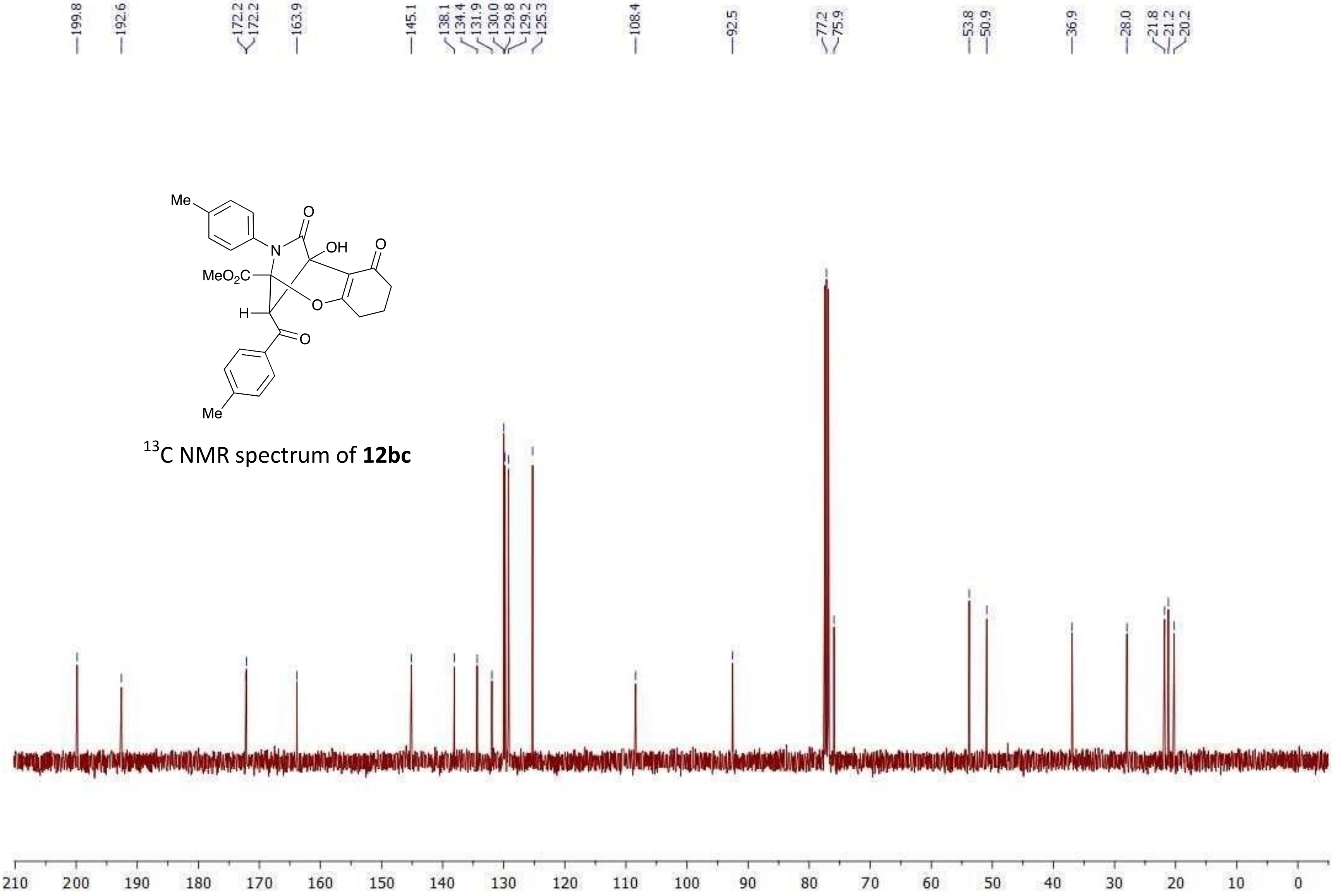

^{13}C NMR spectrum of **12bc**

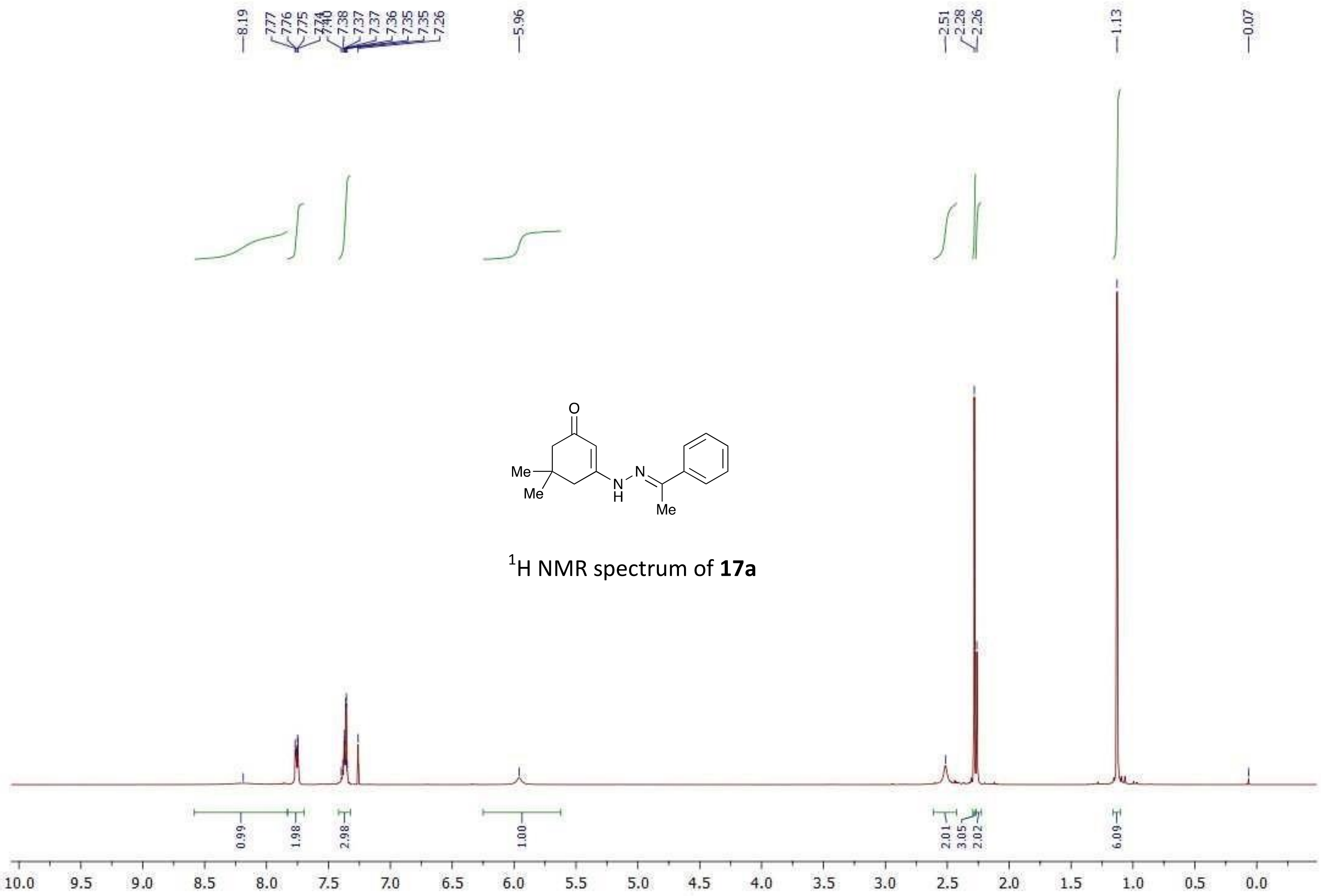

^{1}H NMR spectrum of **17a**

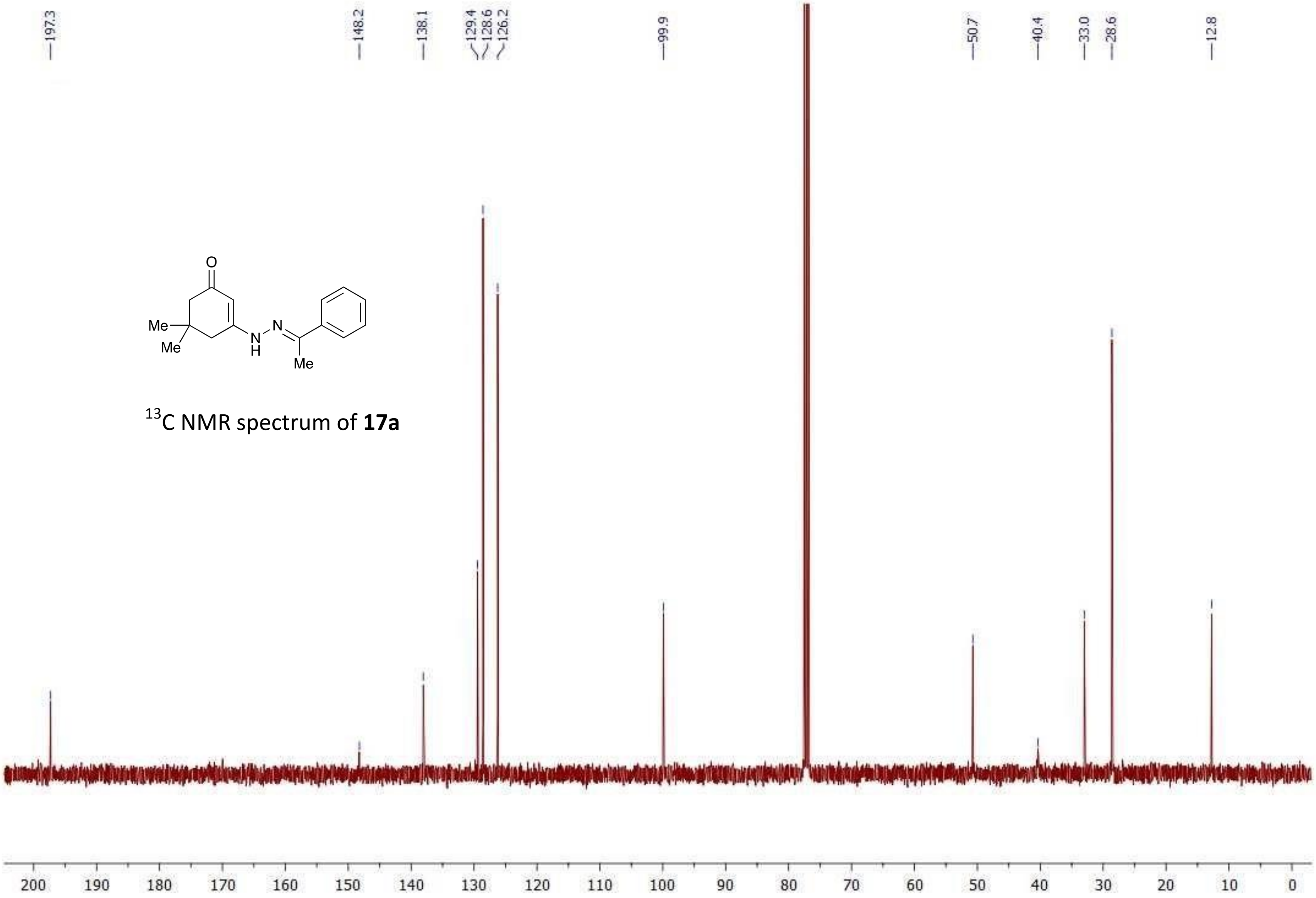

^{13}C NMR spectrum of **17a**

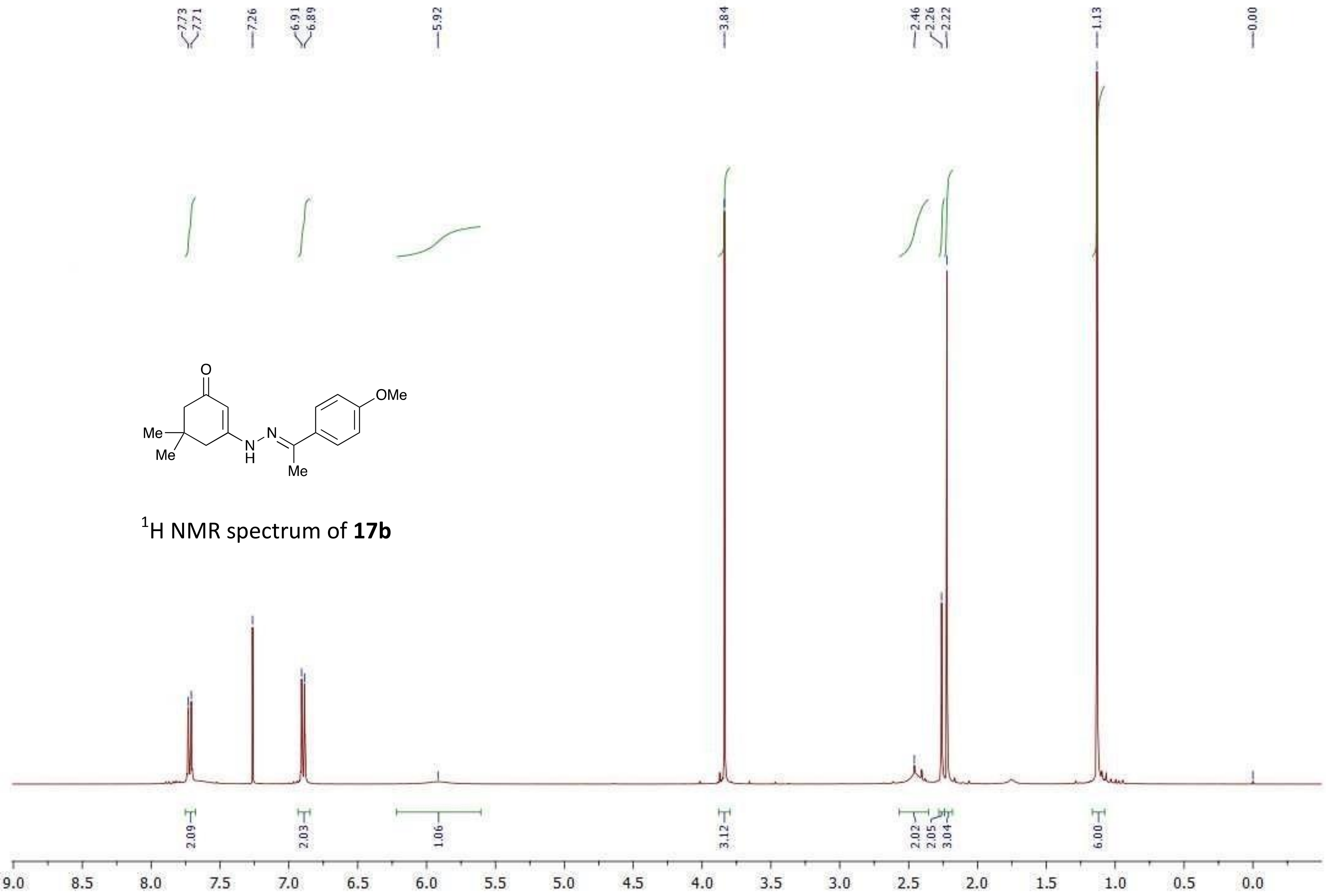

^{1}H NMR spectrum of **17b**

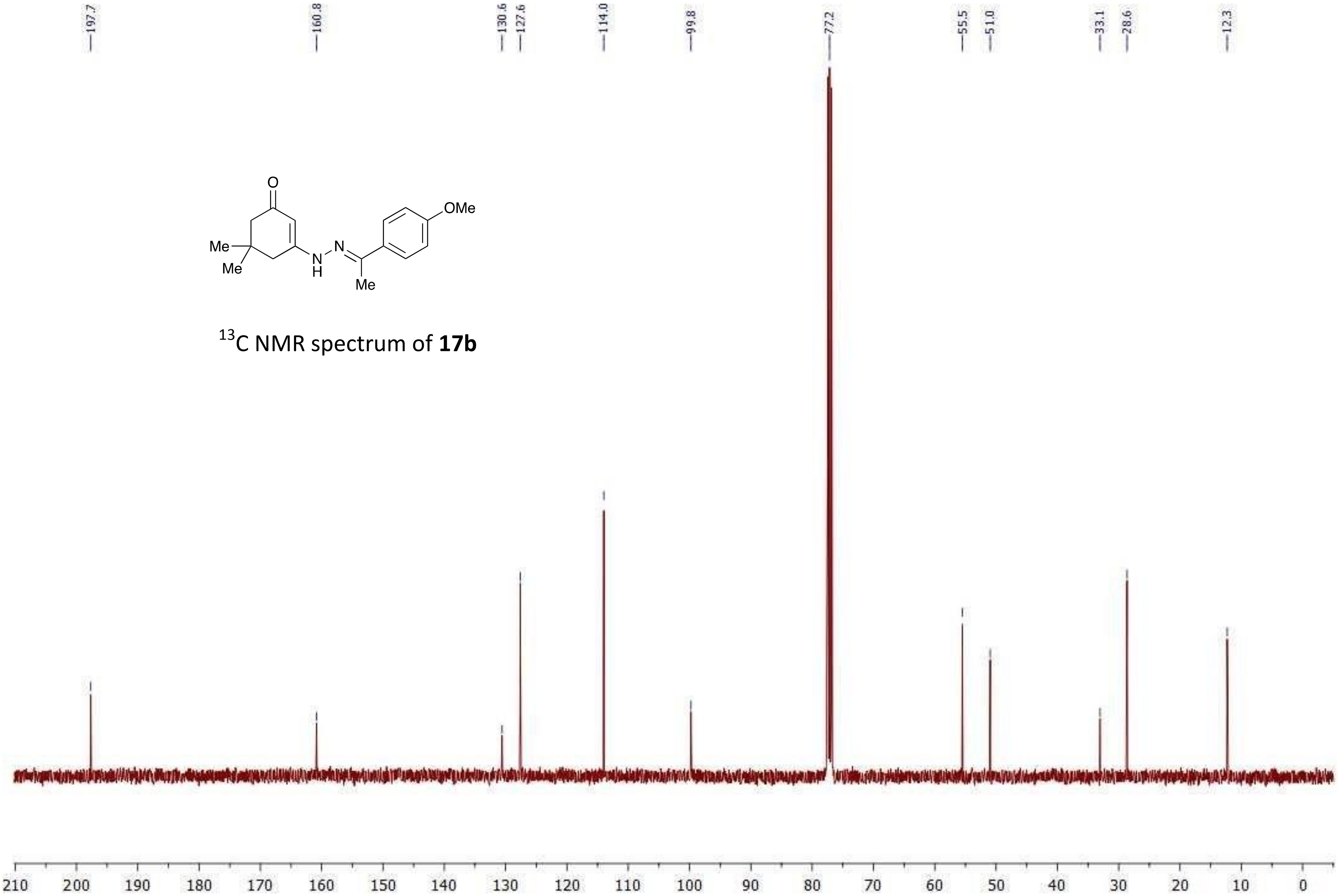

^{13}C NMR spectrum of **17b**

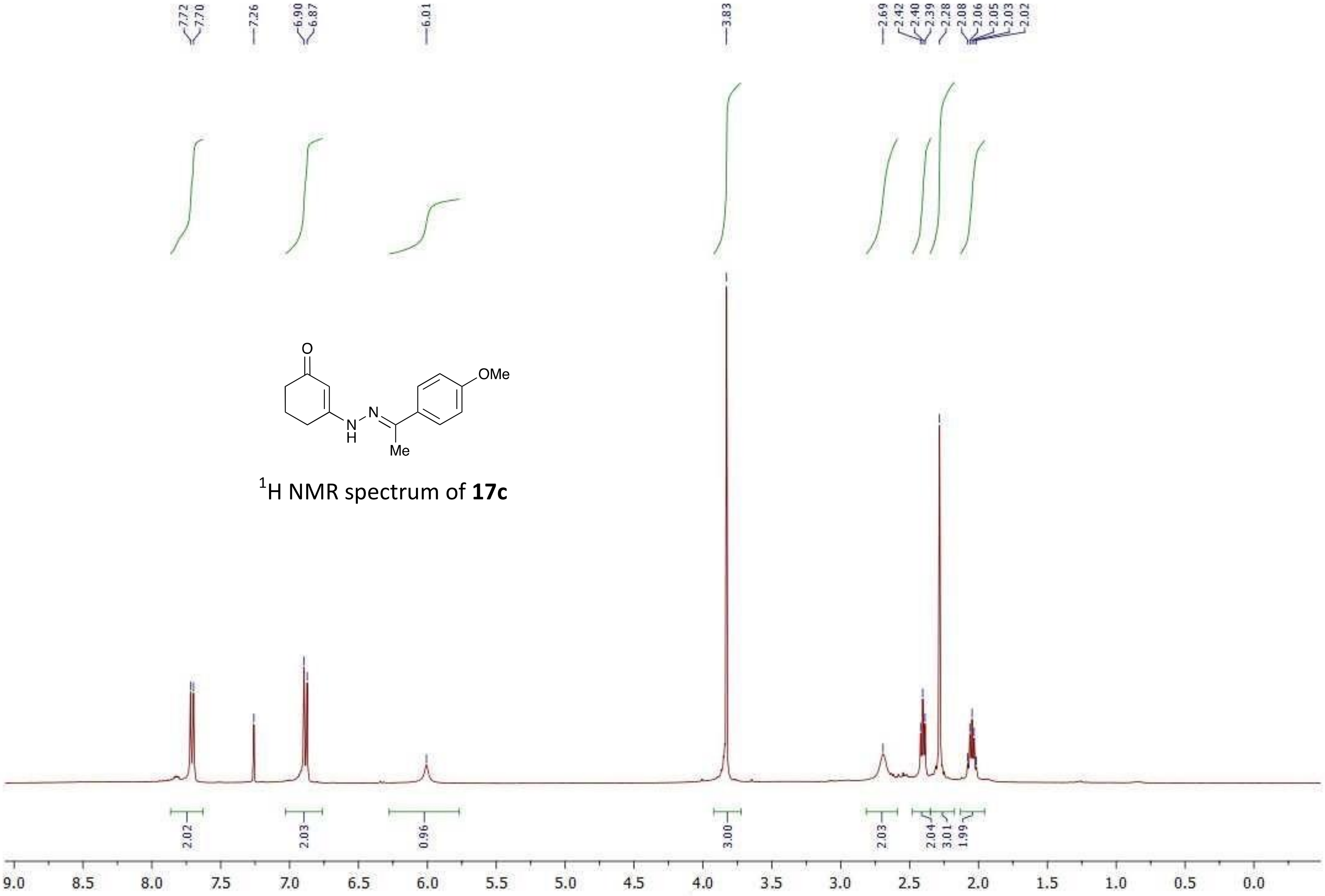

^{1}H NMR spectrum of **17c**

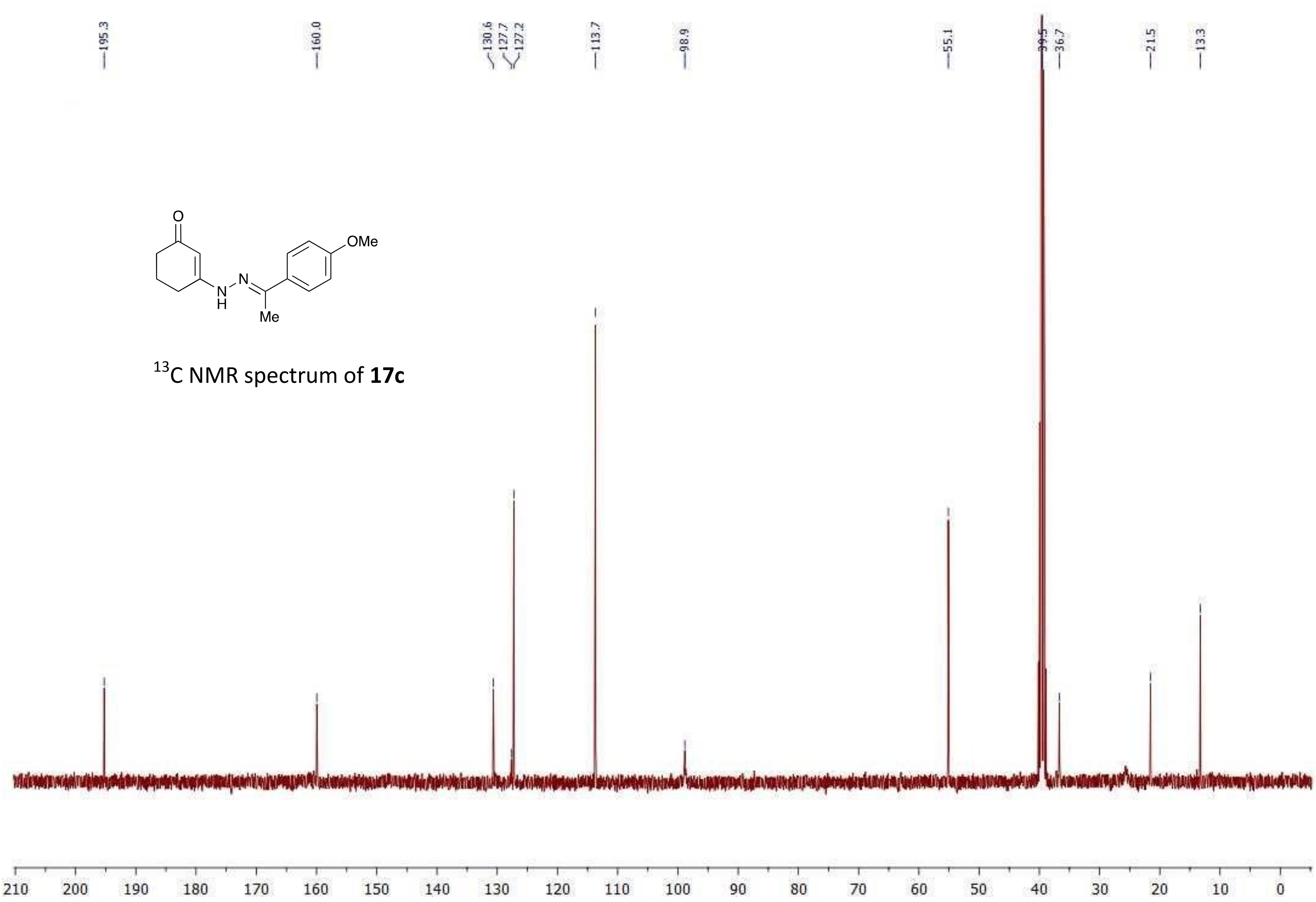

^{13}C NMR spectrum of **17c**

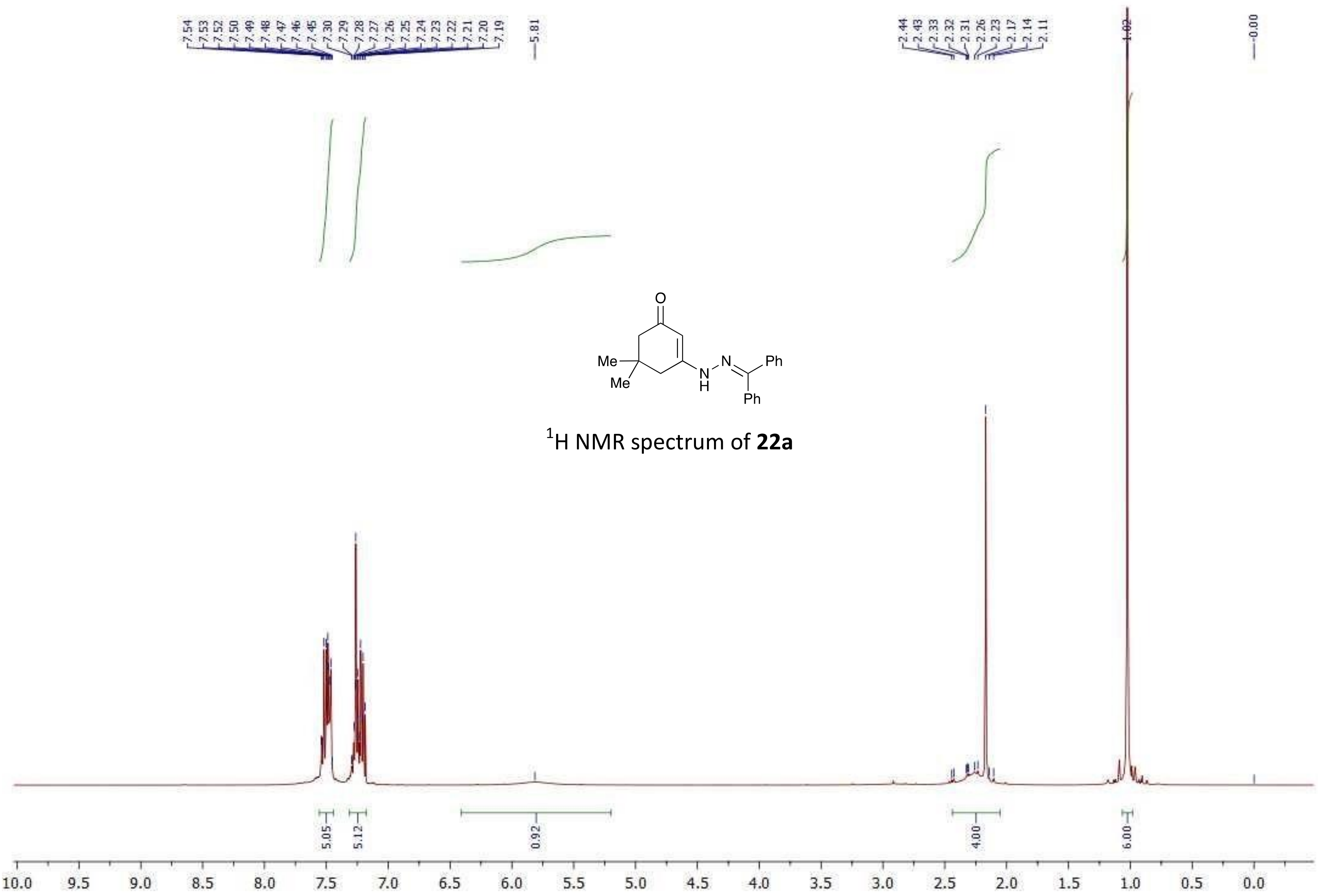

^{1}H NMR spectrum of **22a**

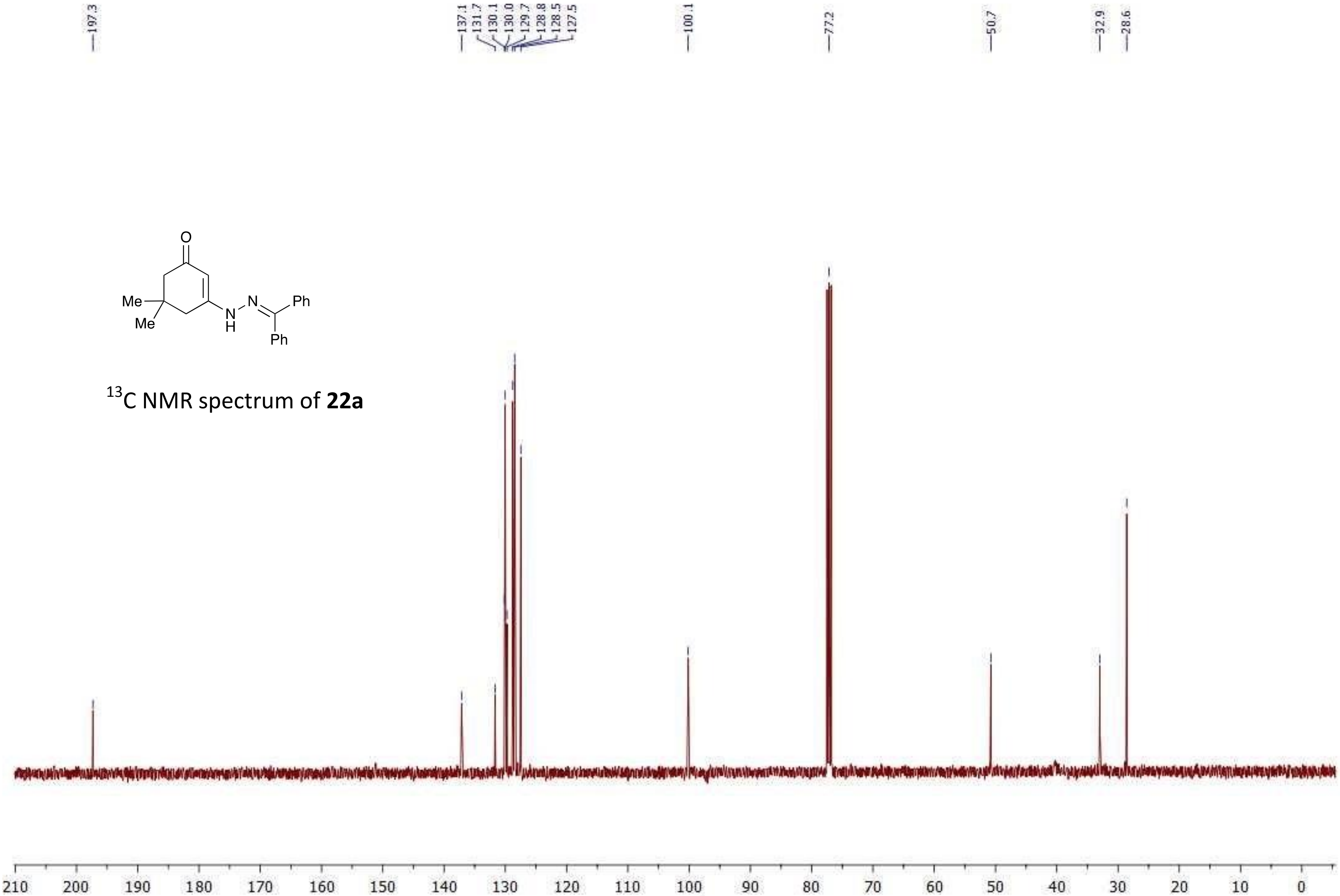

^{13}C NMR spectrum of **22a**

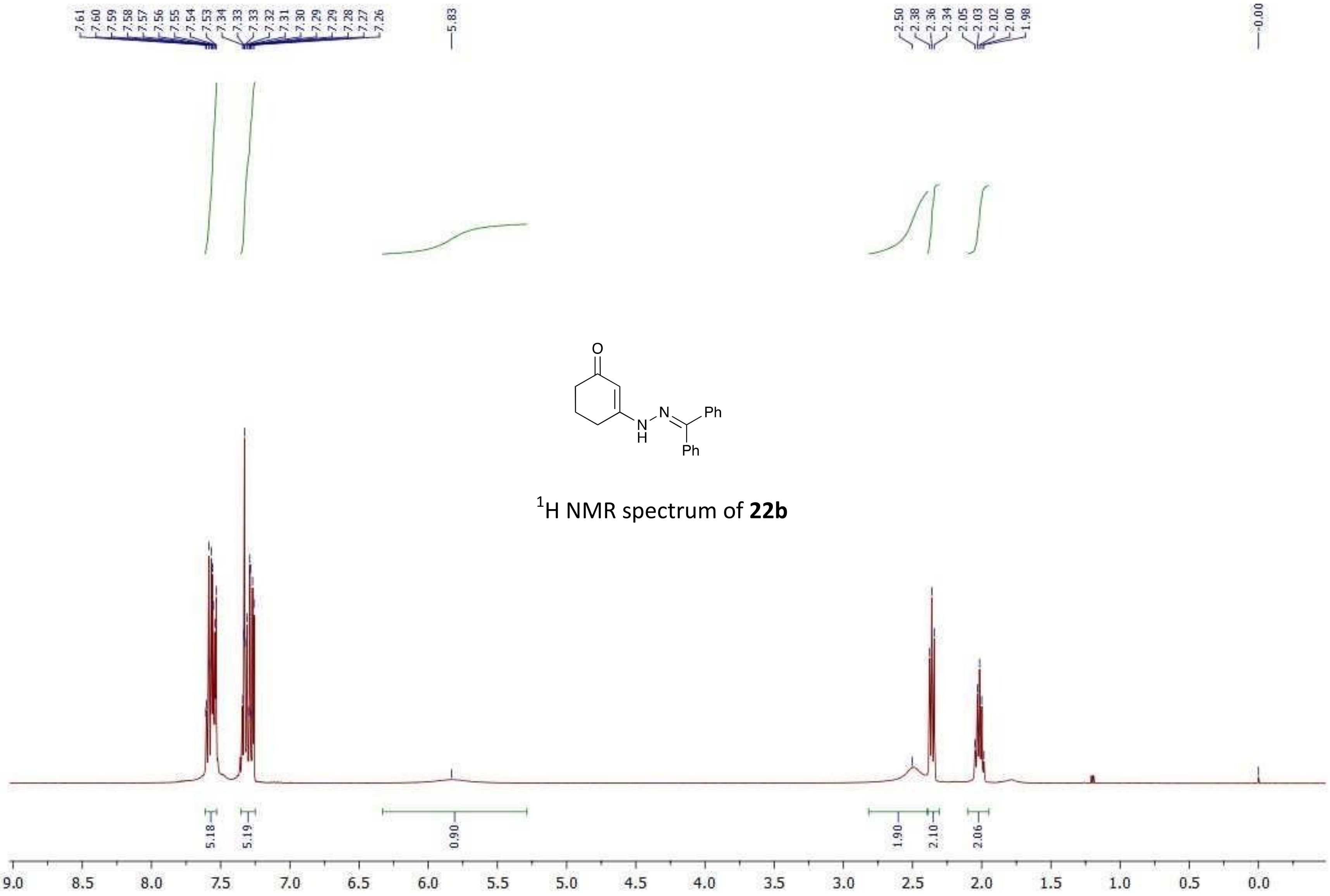

^{1}H NMR spectrum of **22b**

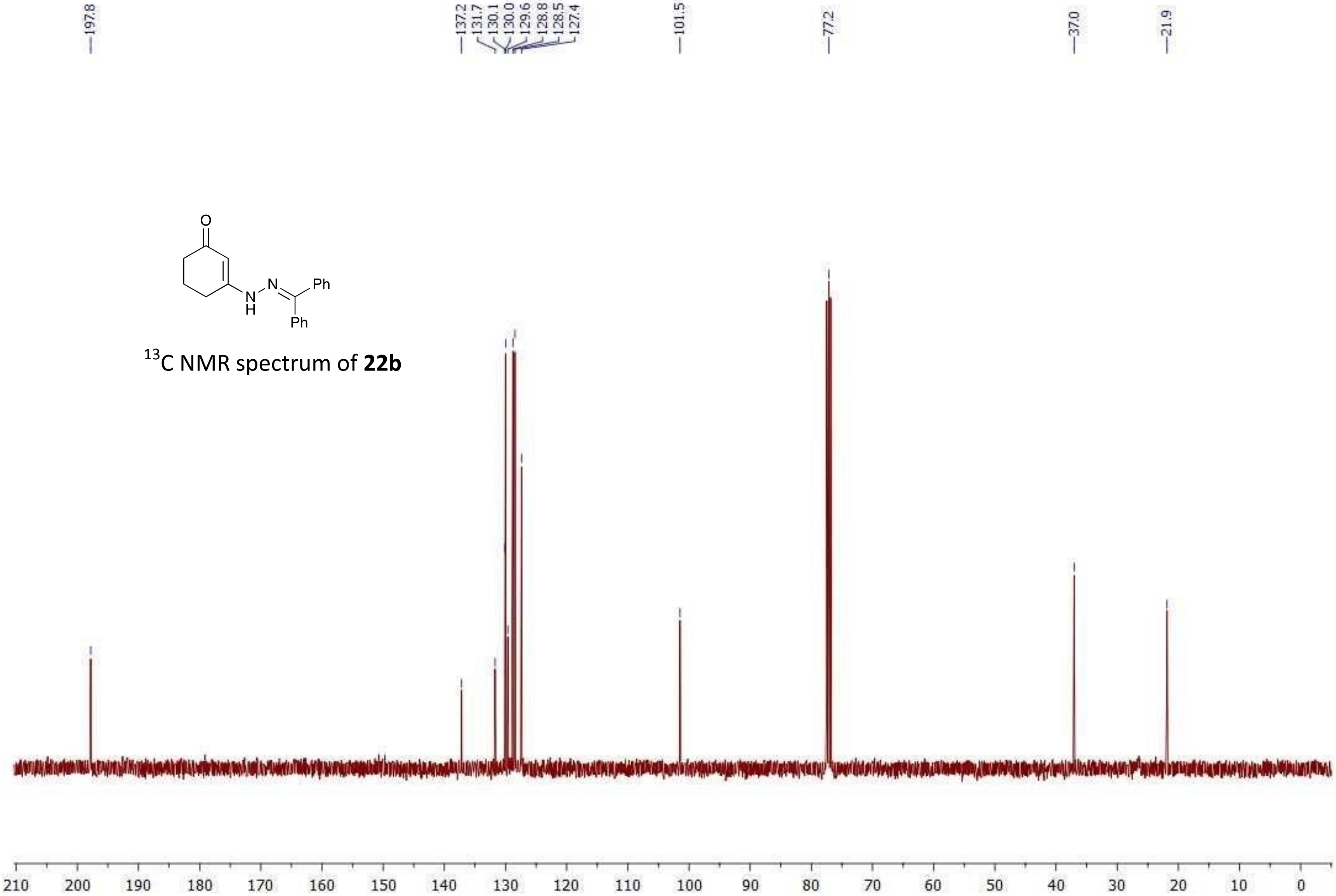

^{13}C NMR spectrum of **22b**

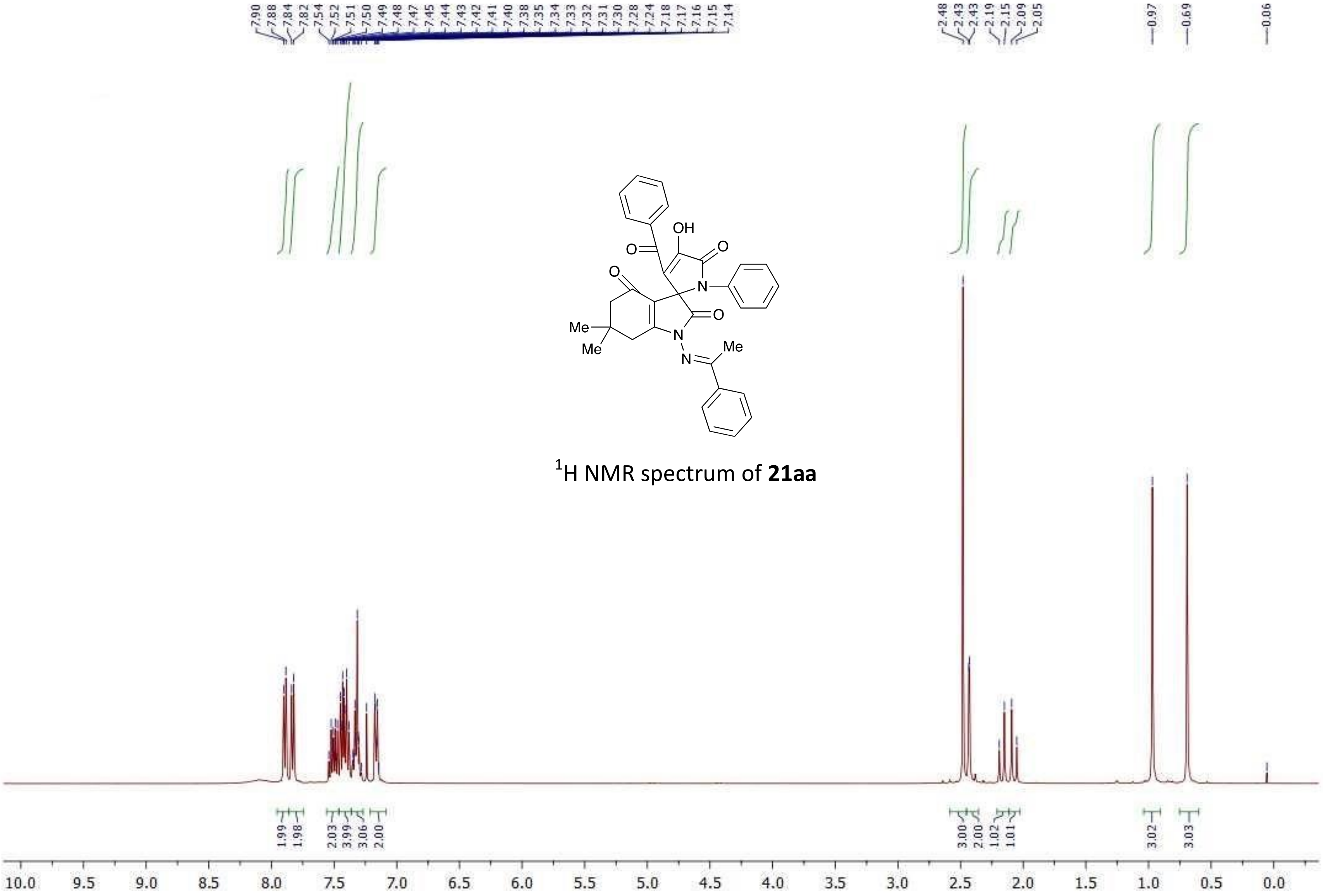

^{1}H NMR spectrum of **21aa**

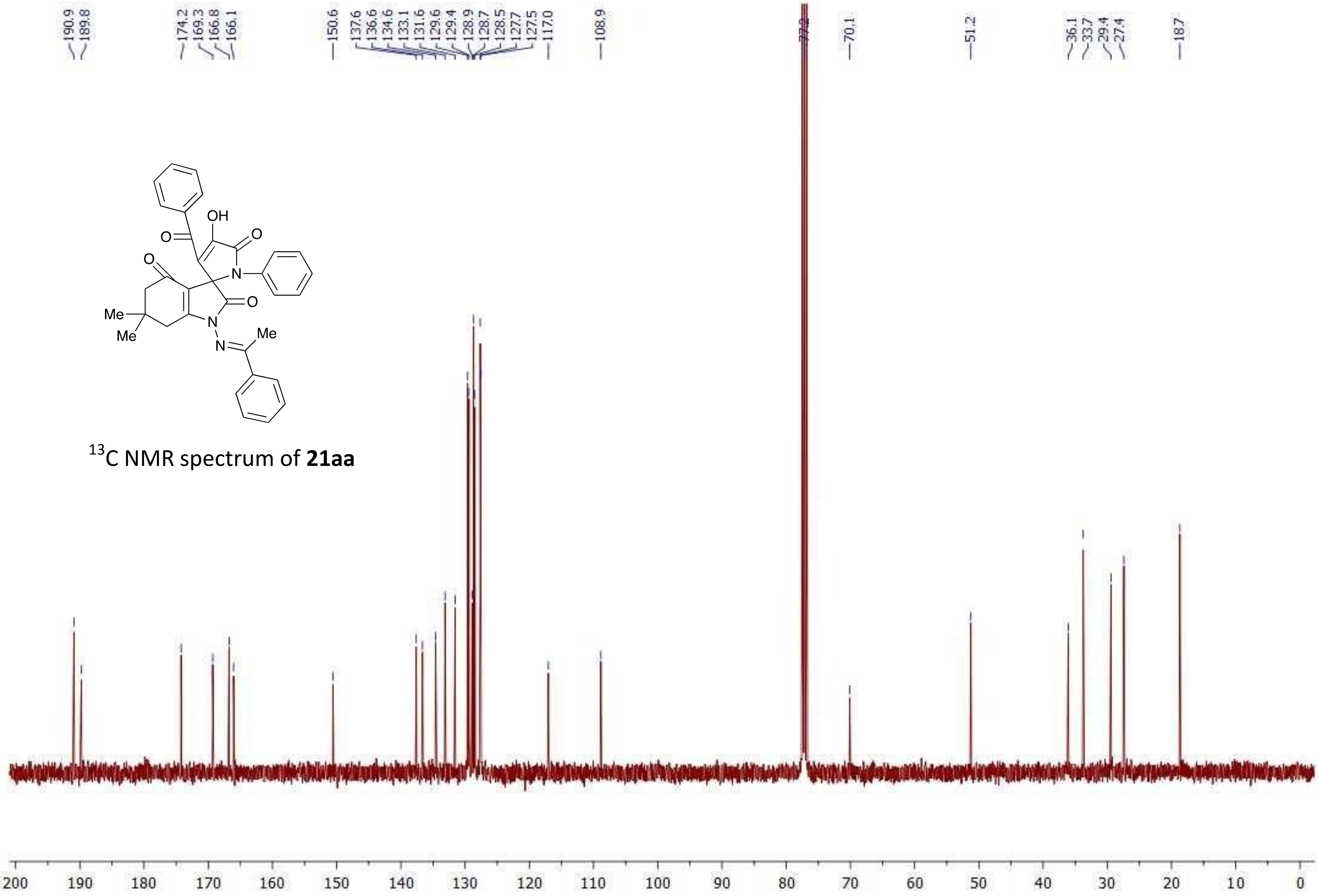

^{13}C NMR spectrum of **21aa**

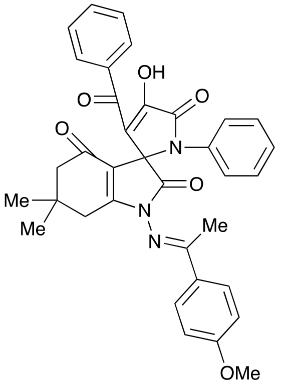

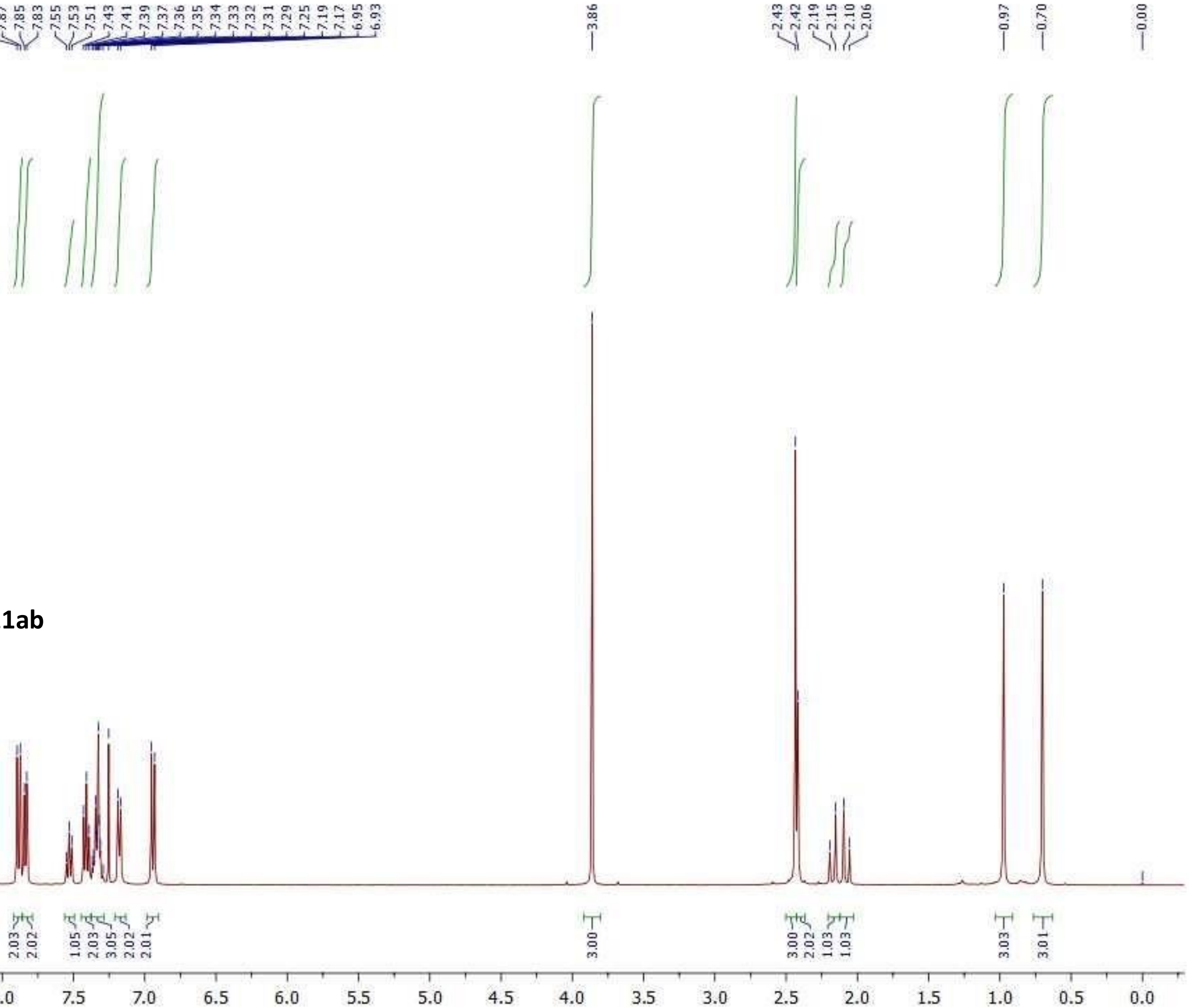

^{1}H NMR spectrum of **21ab**

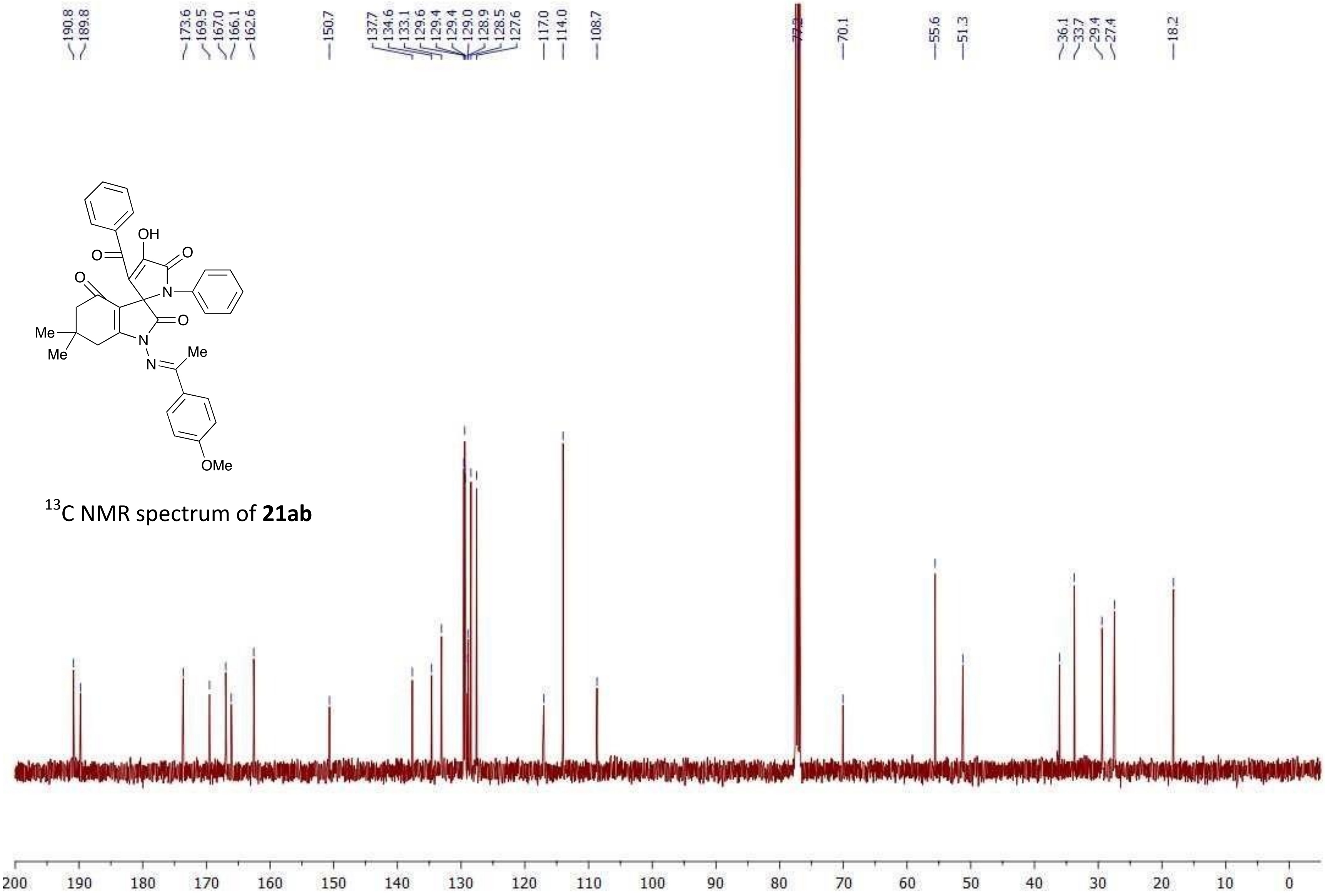

^{13}C NMR spectrum of **21ab**

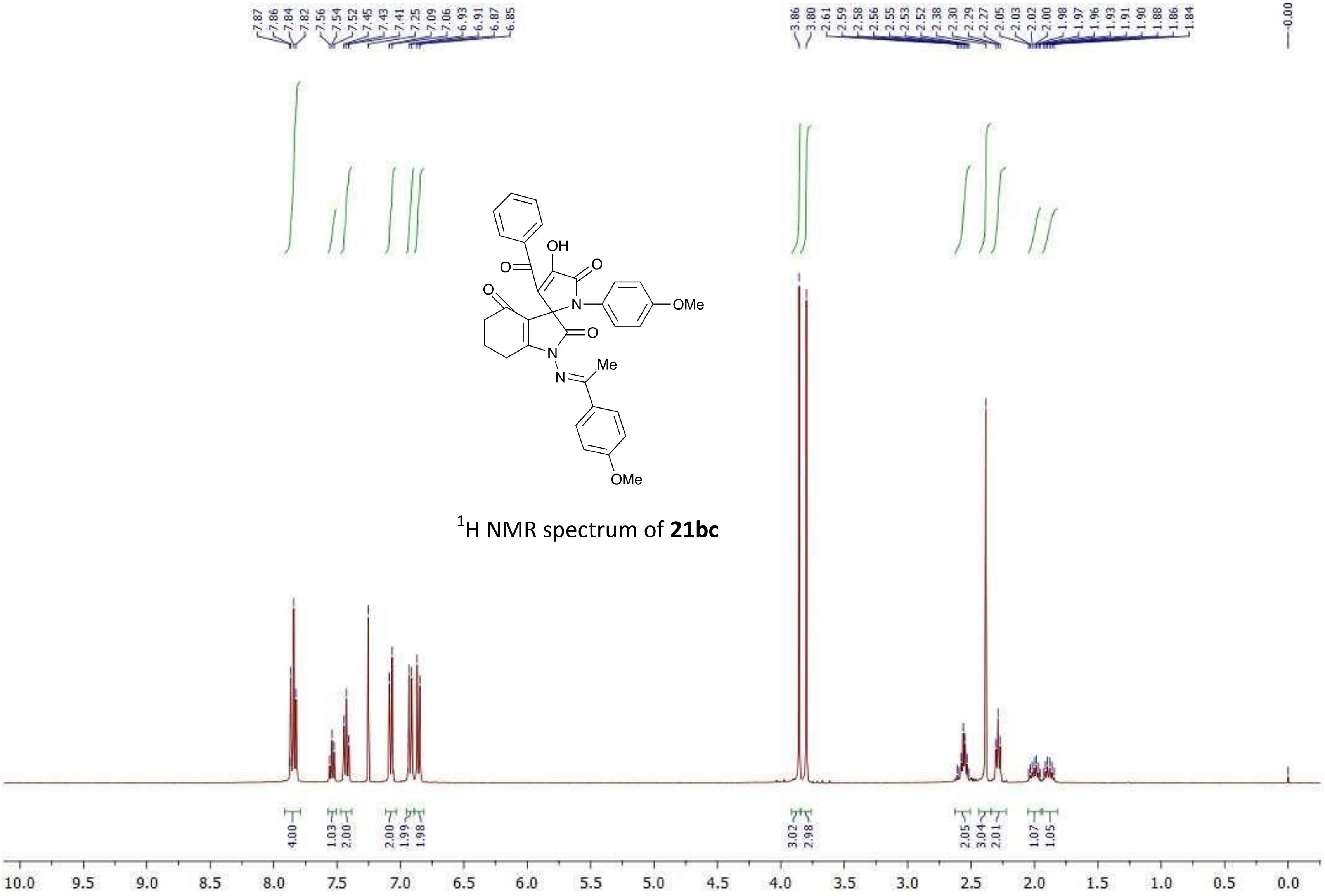

^{1}H NMR spectrum of **21bc**

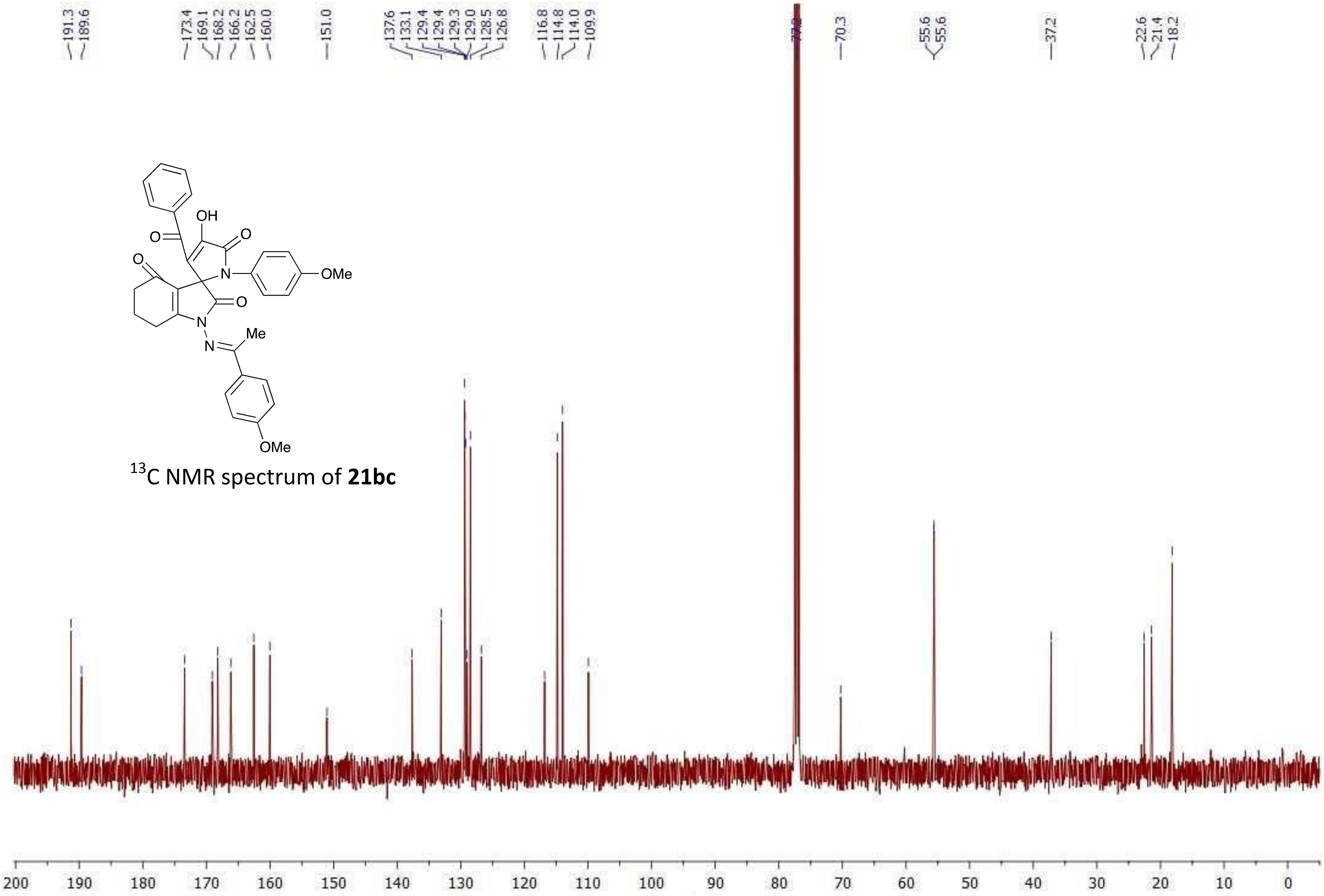

^{13}C NMR spectrum of **21bc**

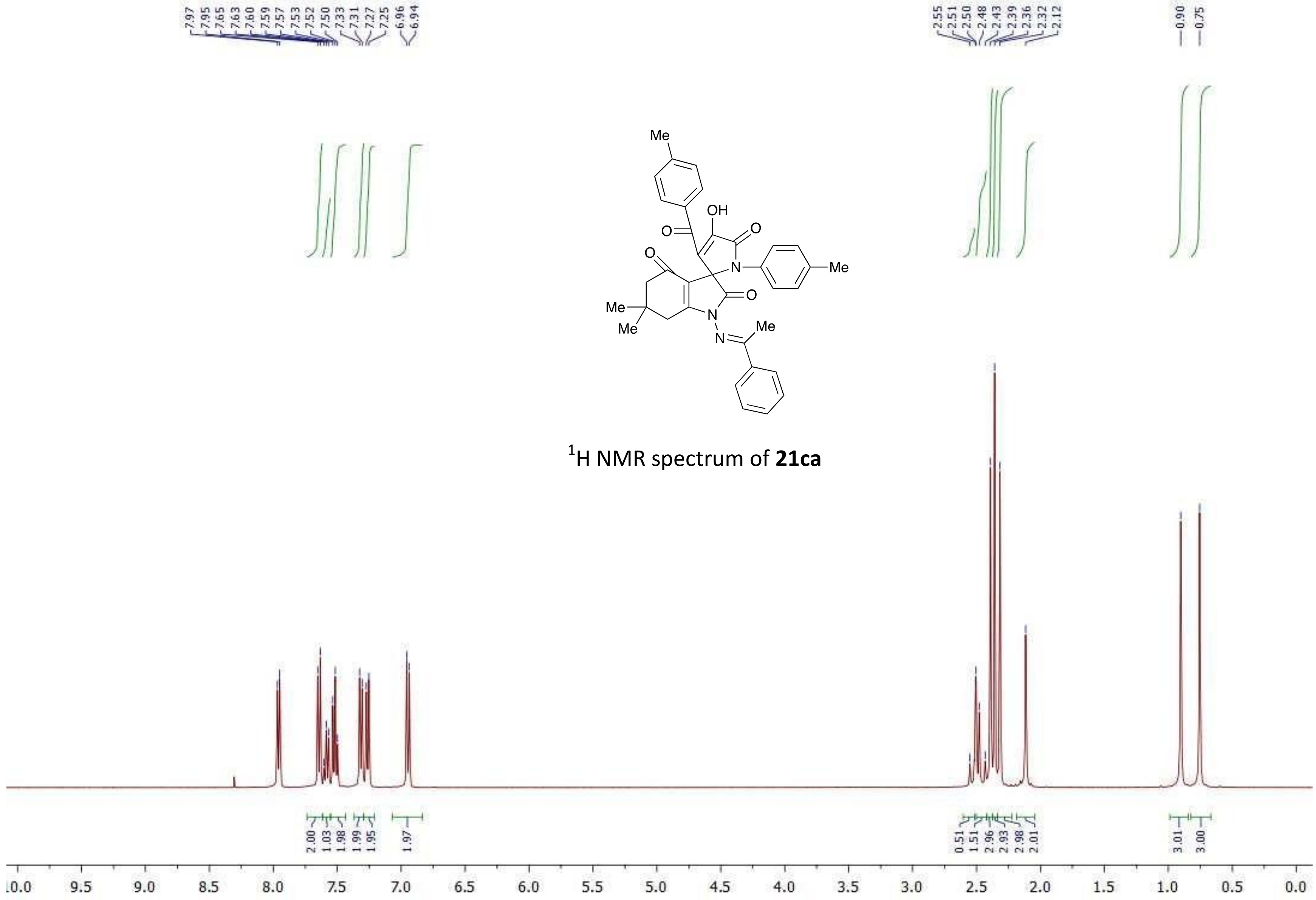

^{1}H NMR spectrum of **21ca**

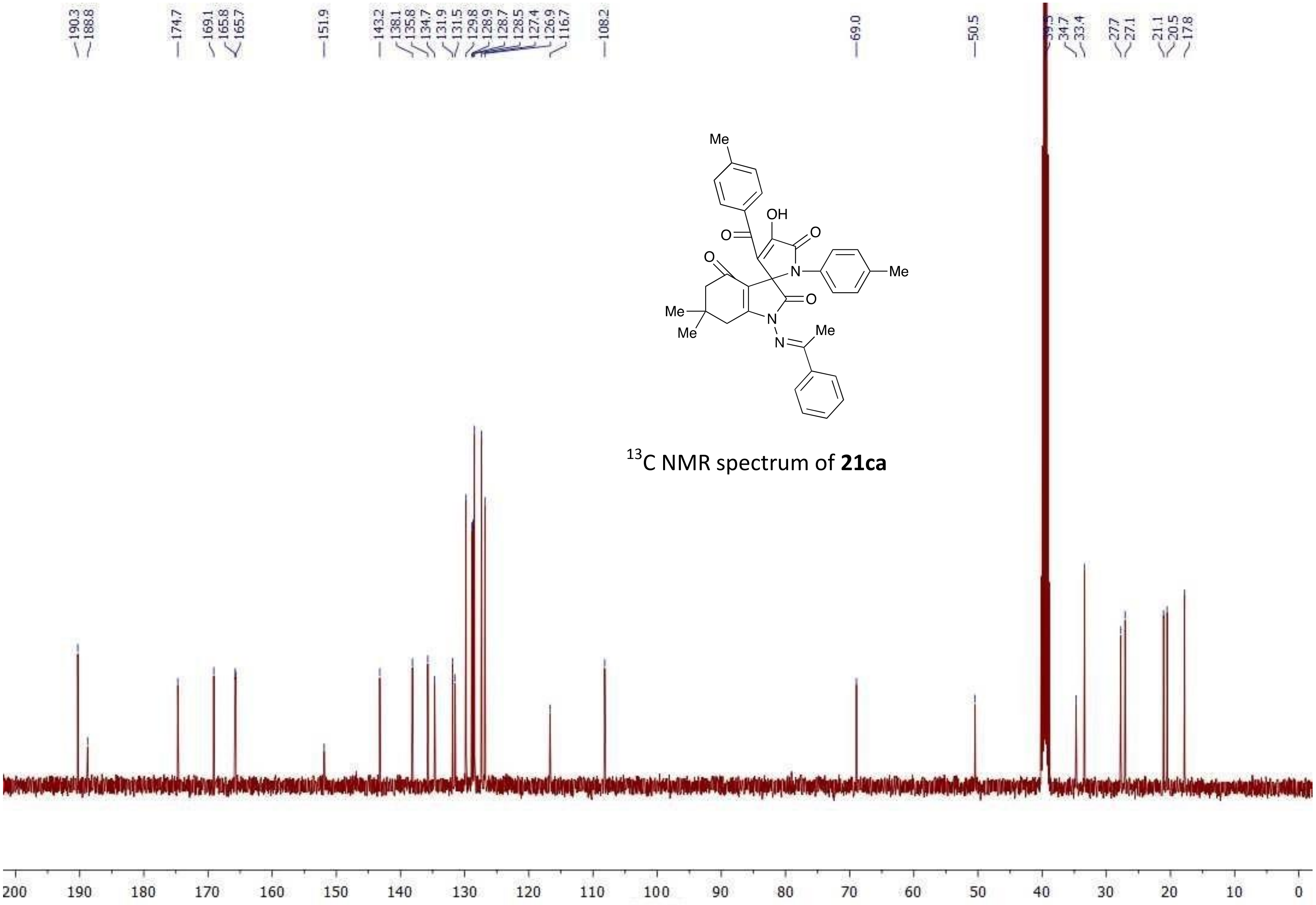

^{13}C NMR spectrum of **21ca**

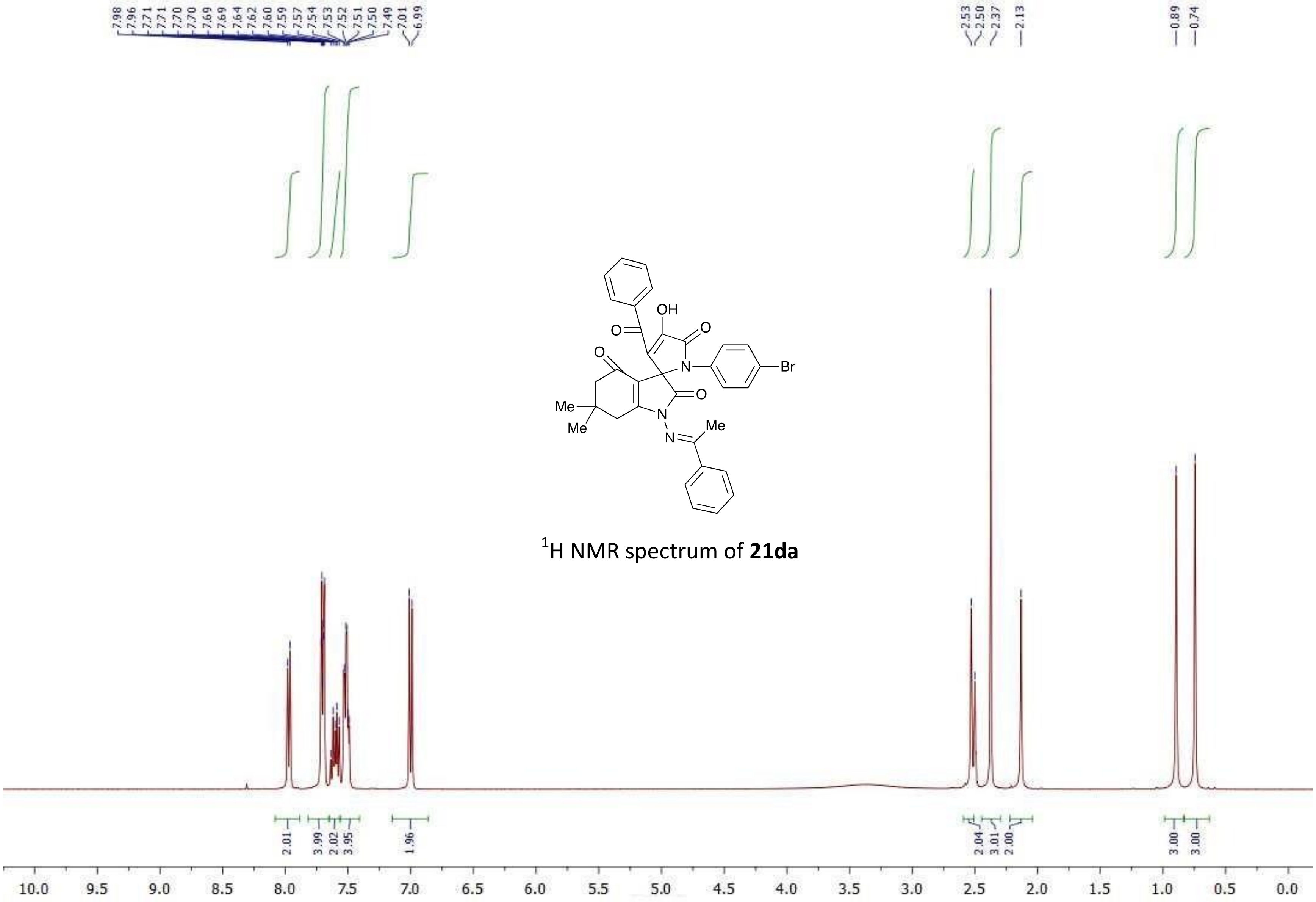

^{1}H NMR spectrum of **21da**

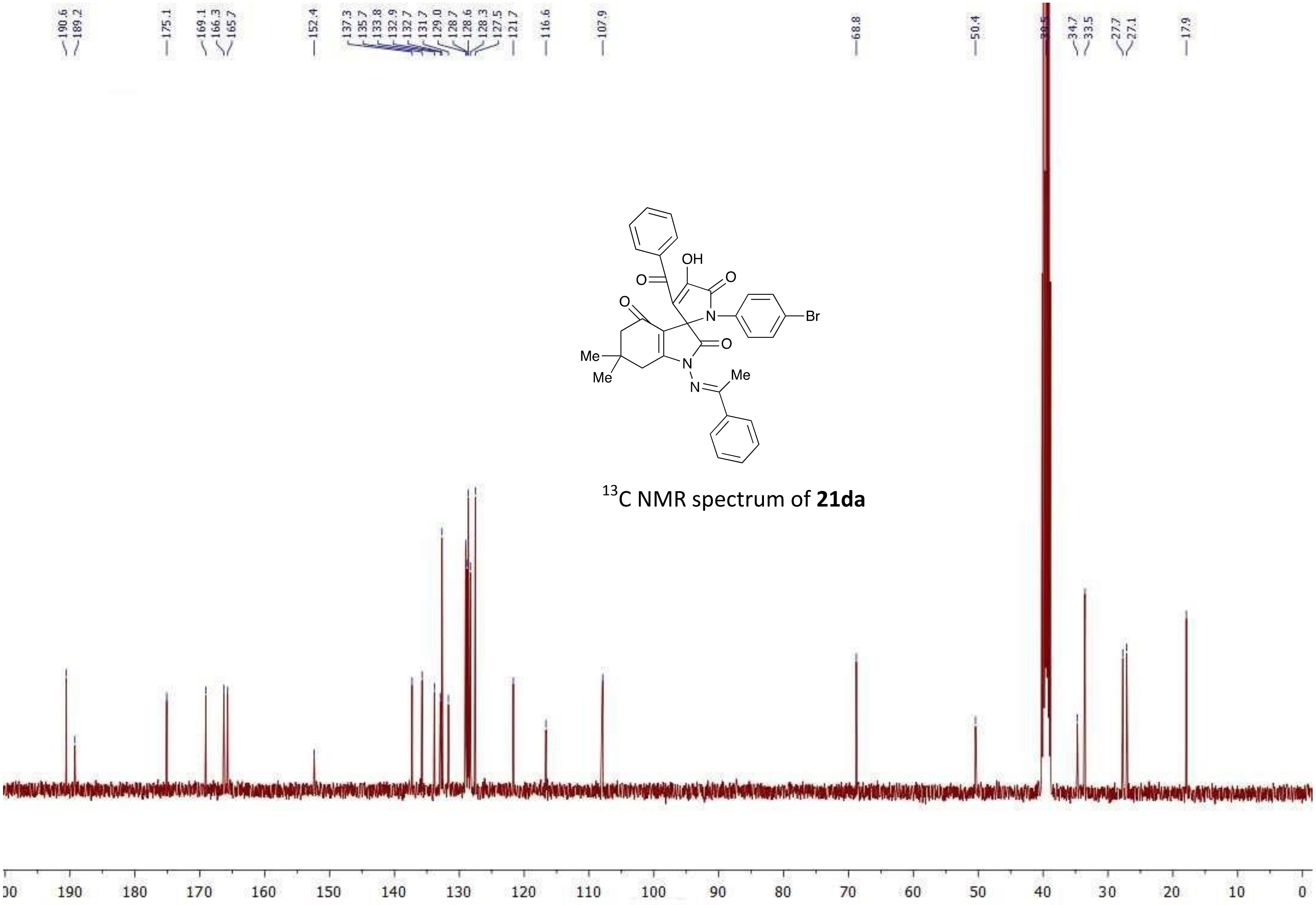

^{13}C NMR spectrum of **21da**

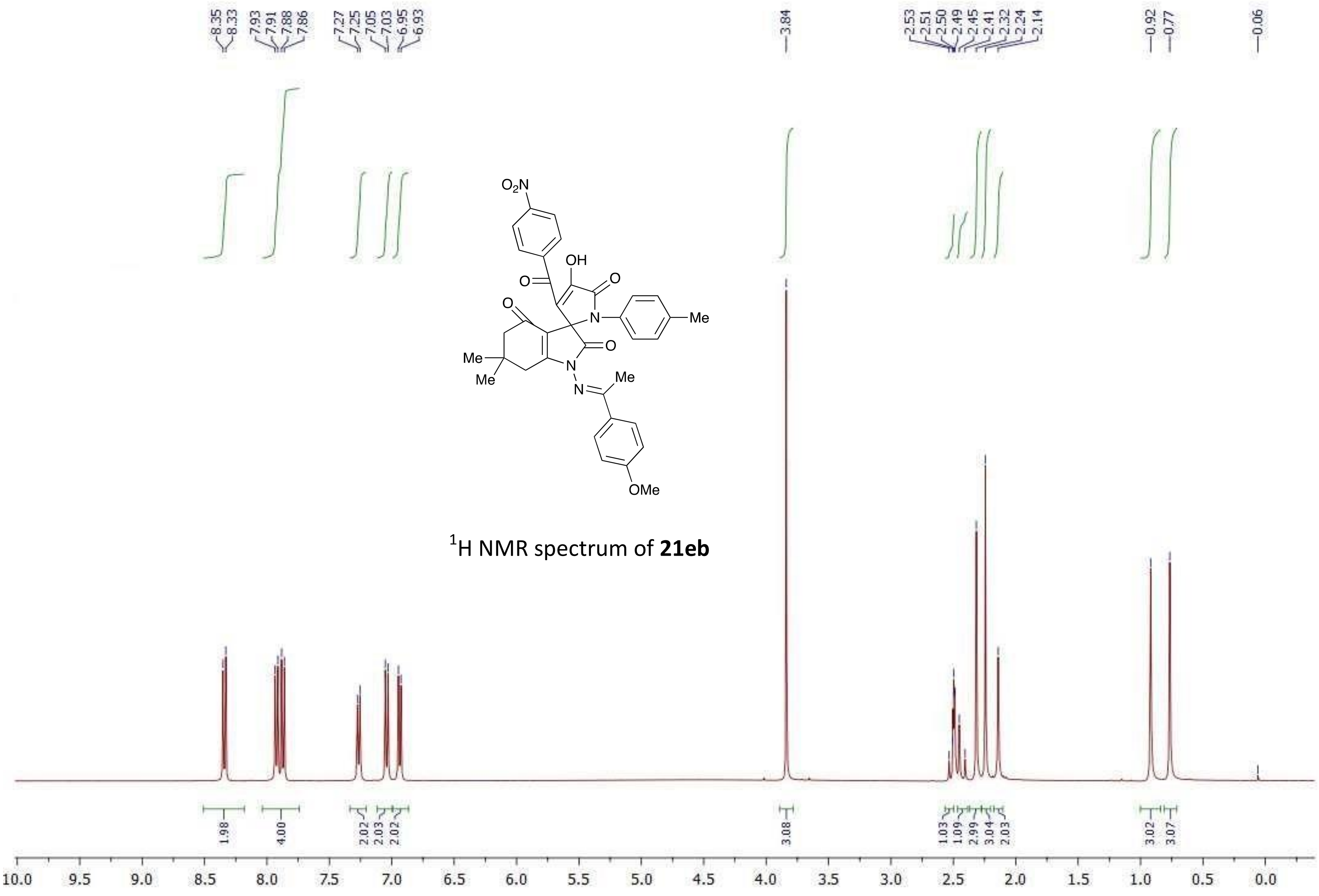

^{1}H NMR spectrum of **21eb**

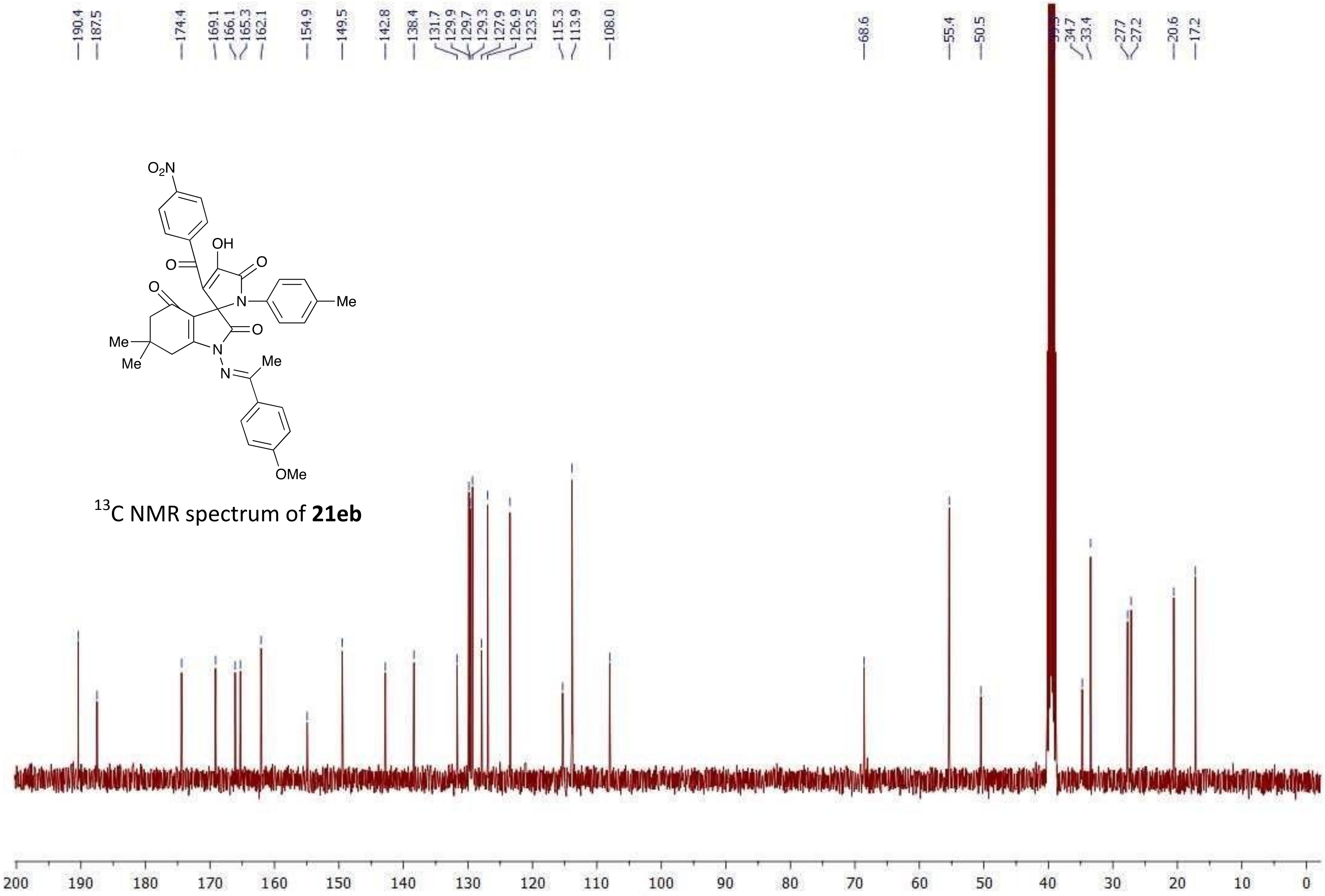

^{13}C NMR spectrum of **21eb**

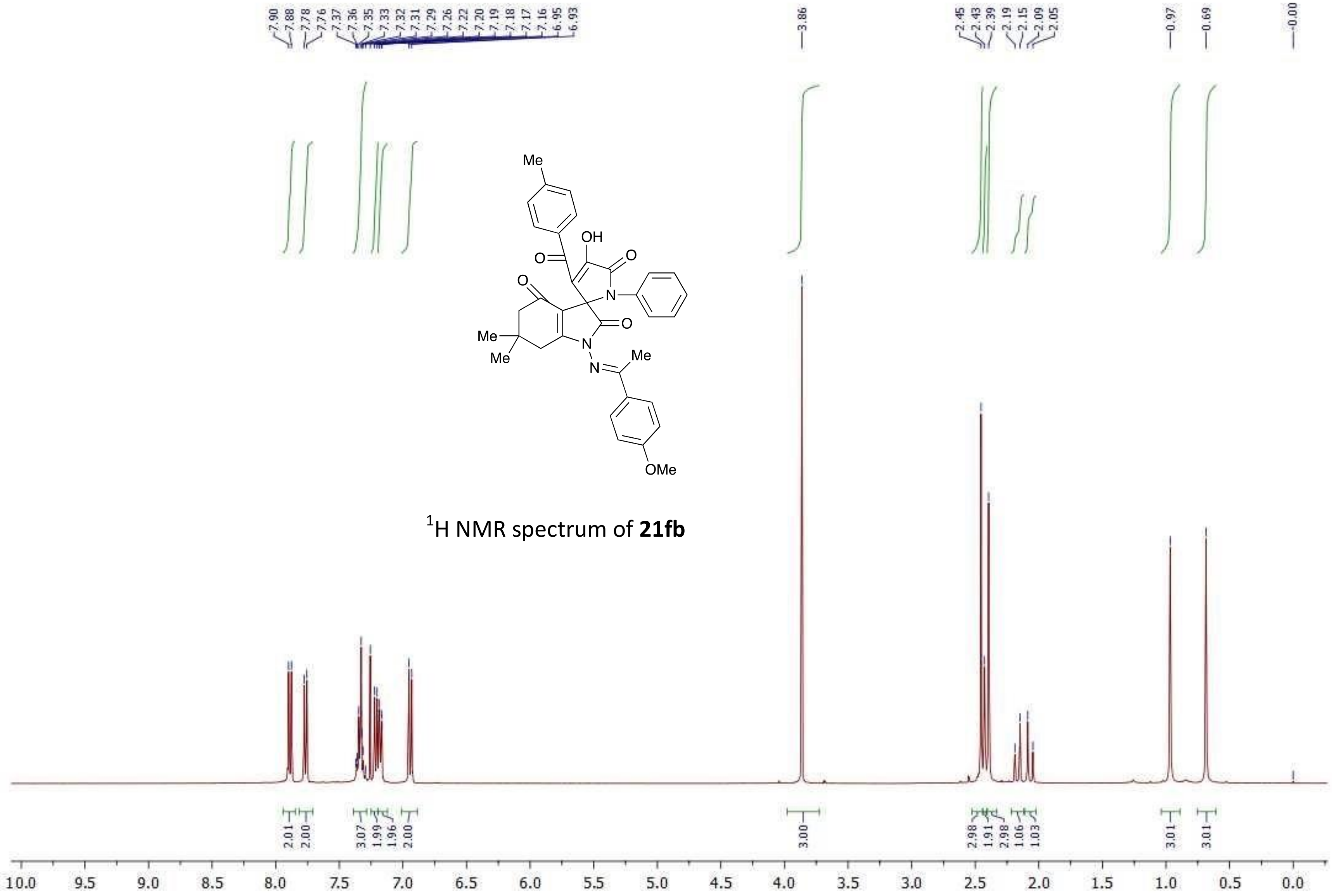

^{1}H NMR spectrum of **21fb**

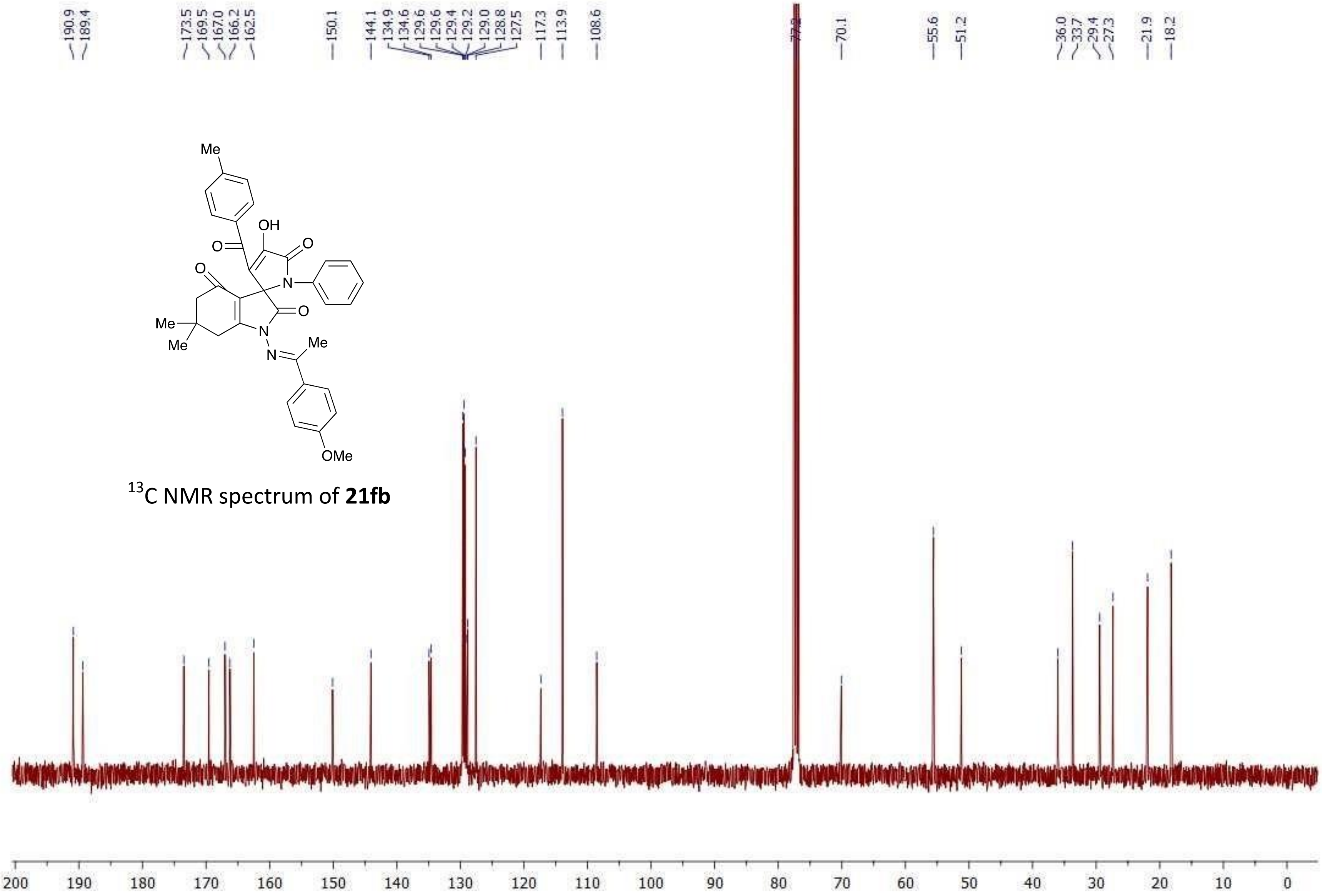

^{13}C NMR spectrum of **21fb**

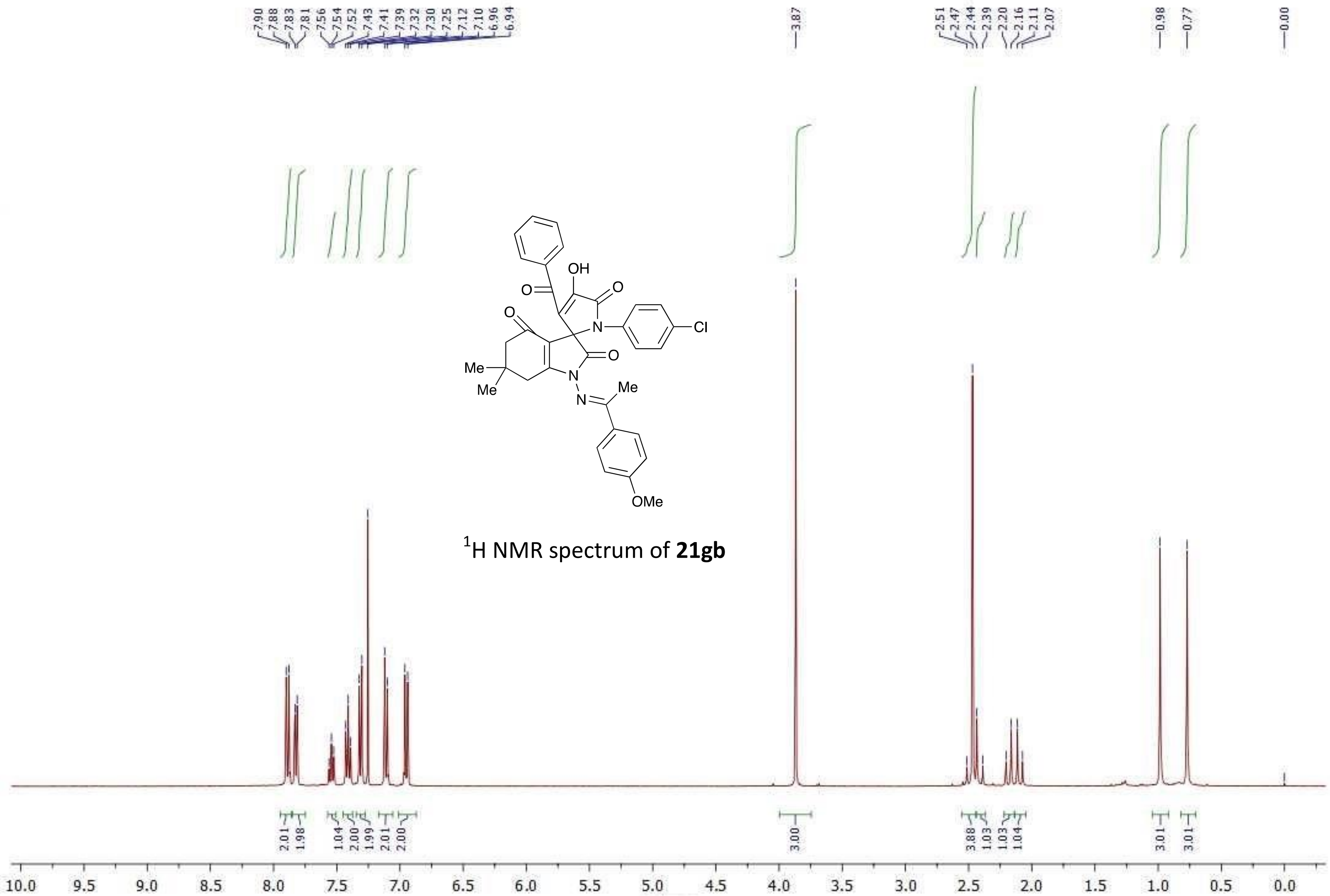

^{1}H NMR spectrum of **21gb**

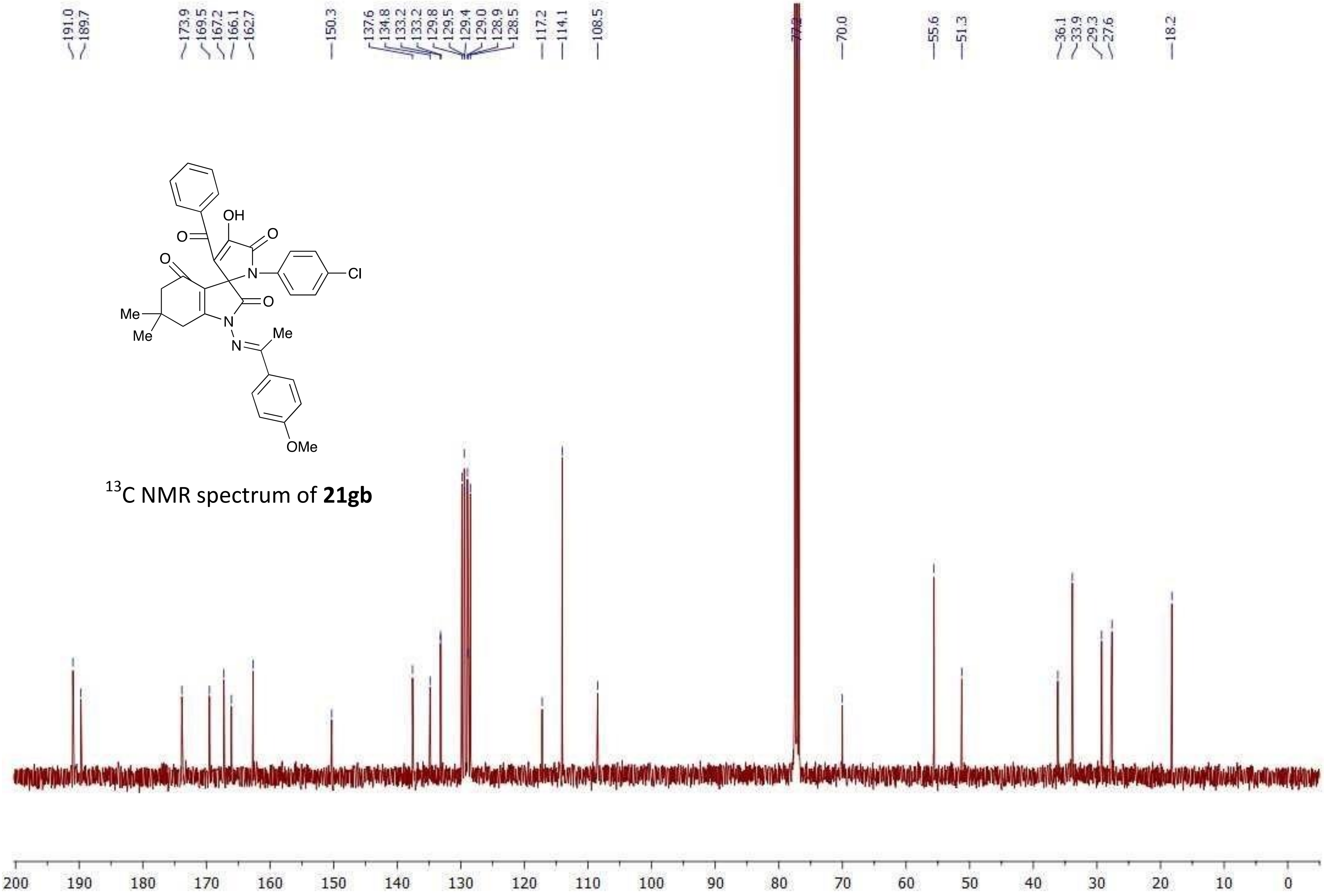

^{13}C NMR spectrum of **21gb**

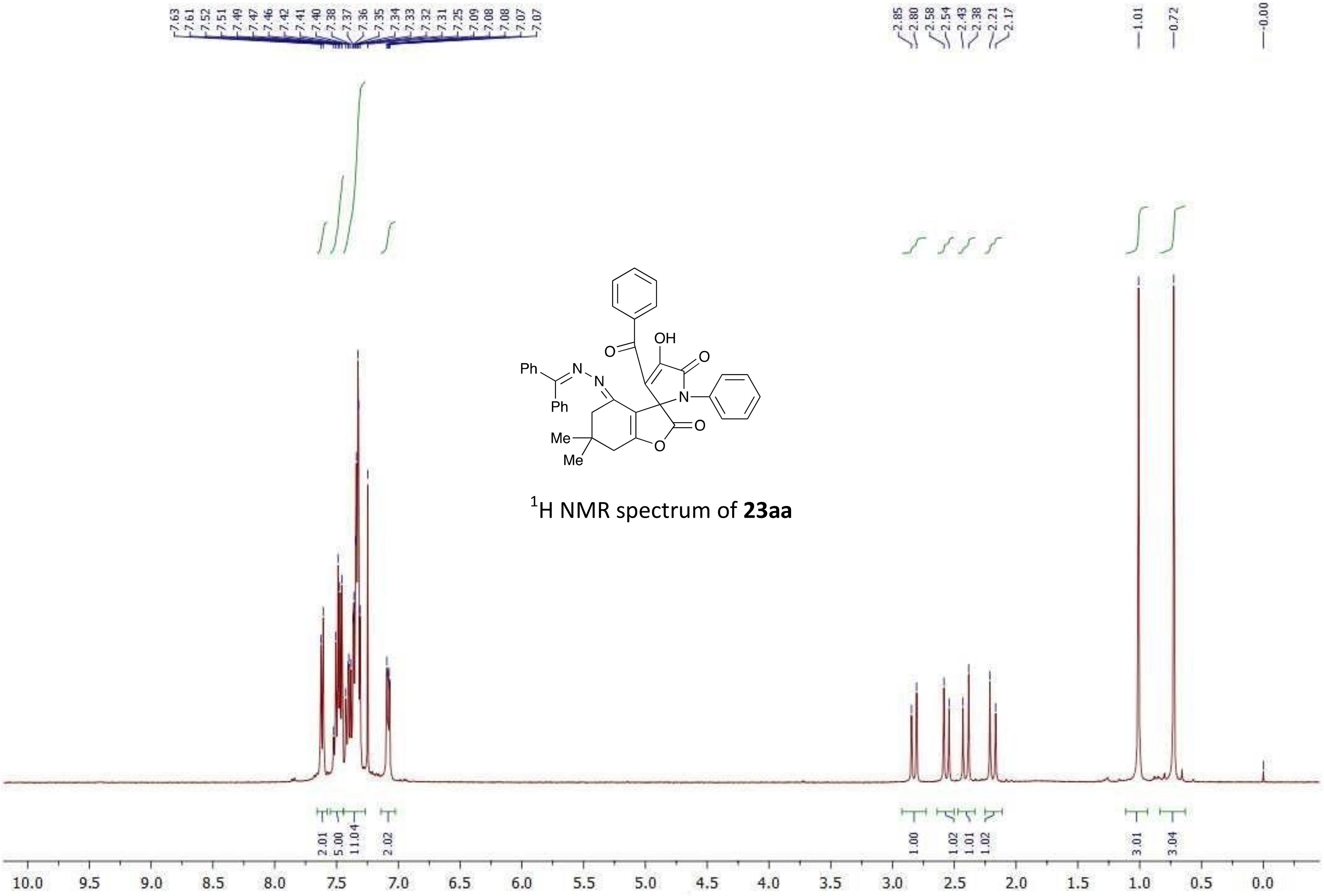

^{1}H NMR spectrum of **23aa**

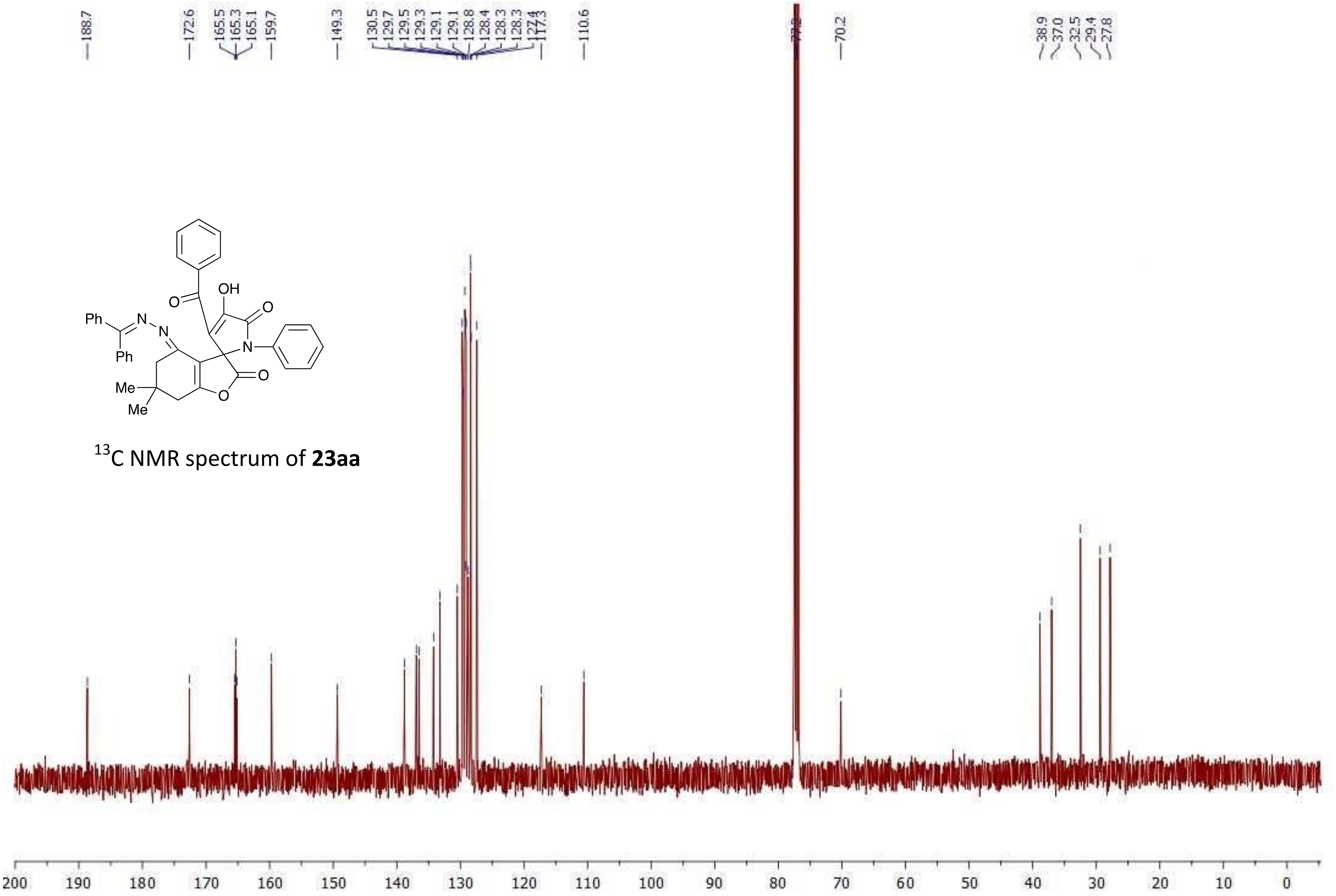

^{13}C NMR spectrum of **23aa**

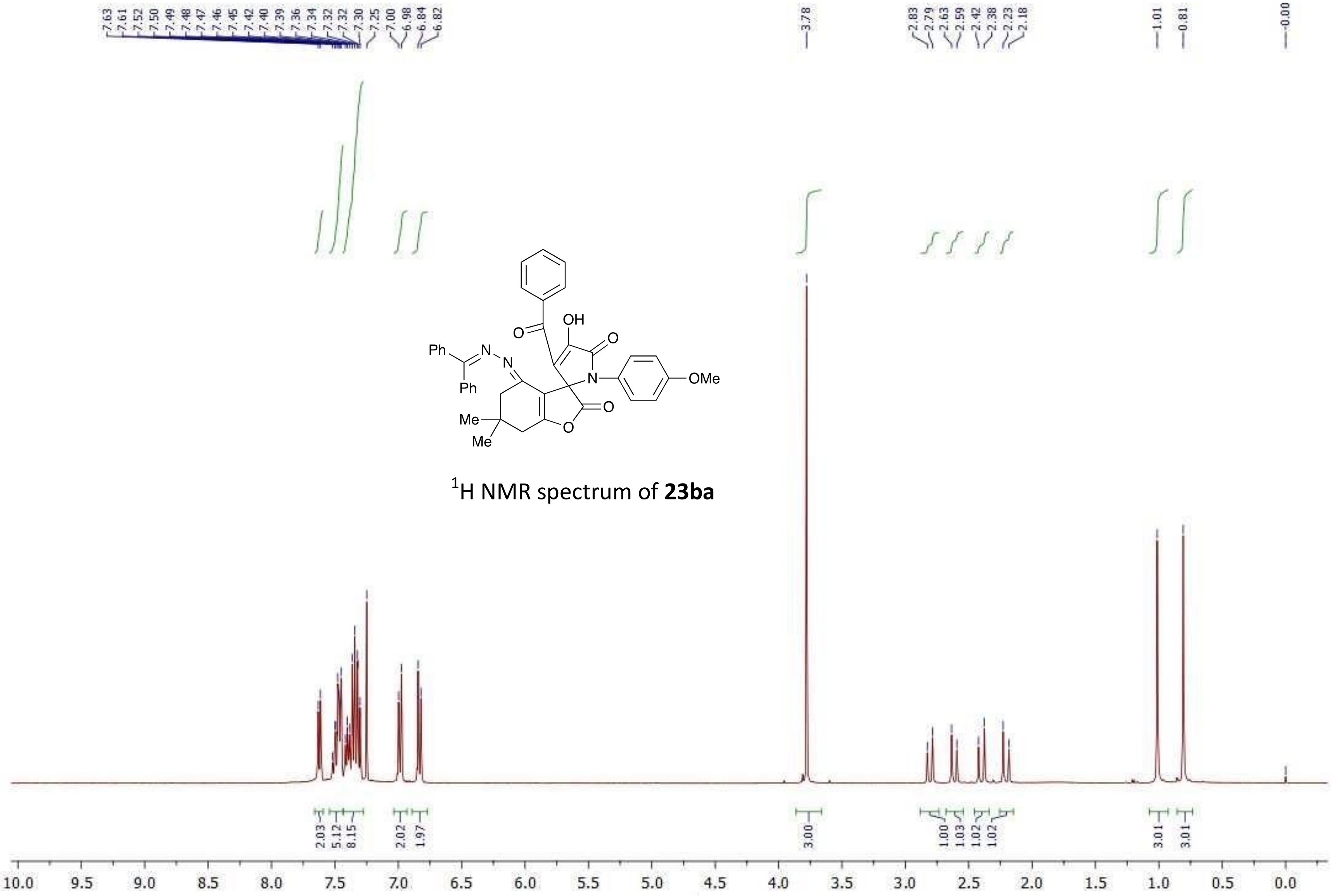

^{1}H NMR spectrum of **23ba**

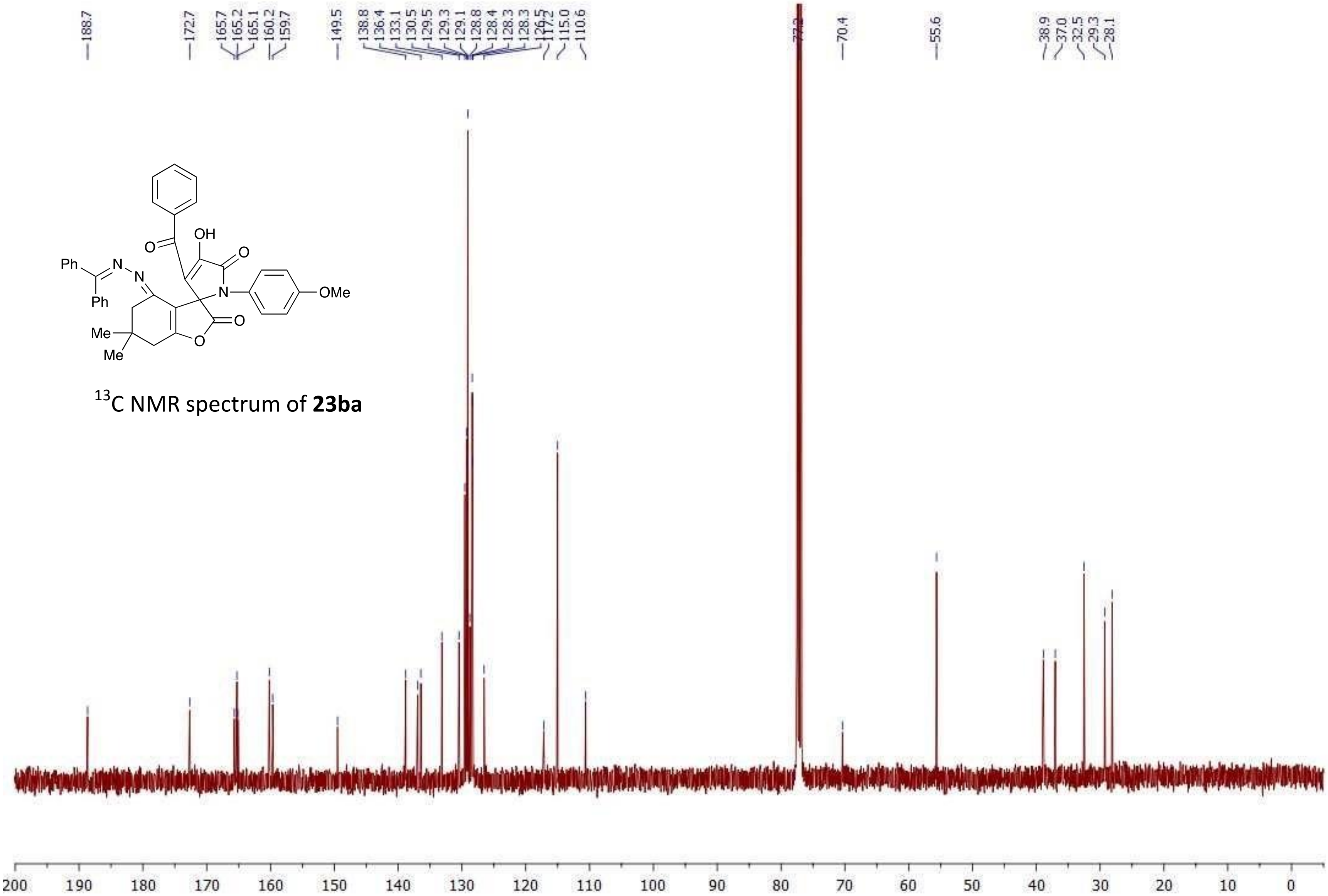

^{13}C NMR spectrum of **23ba**

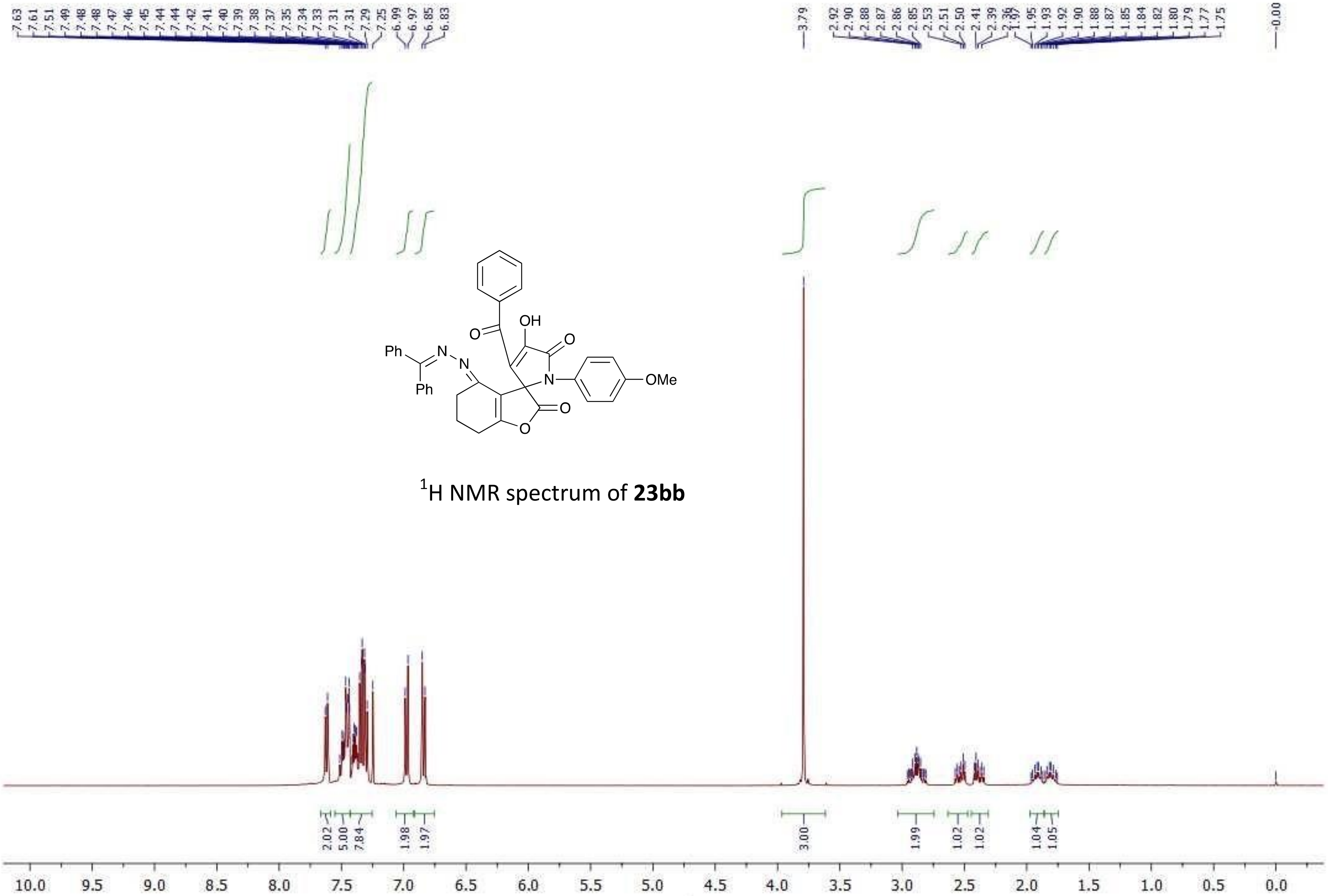

^{1}H NMR spectrum of **23bb**

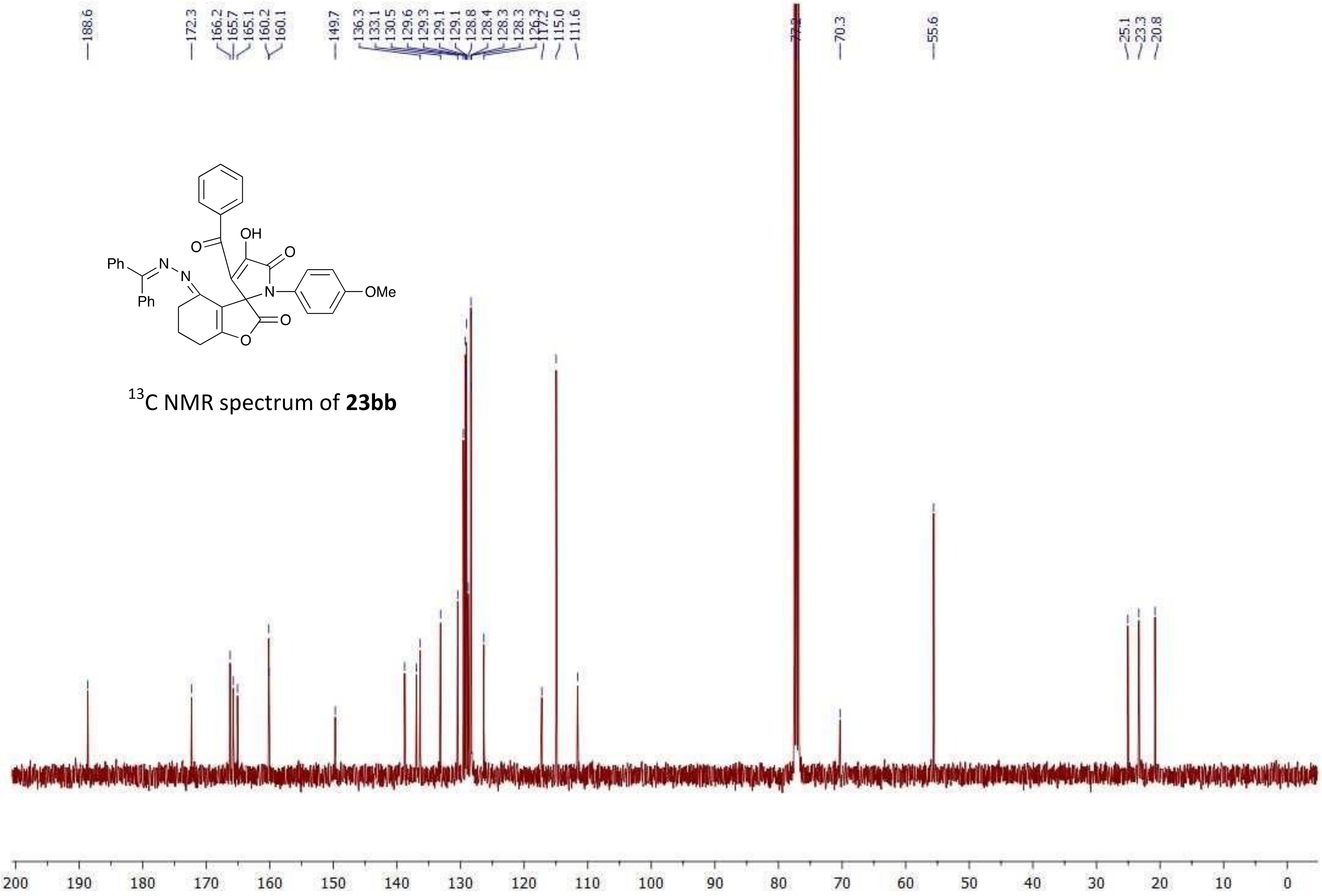

^{13}C NMR spectrum of **23bb**

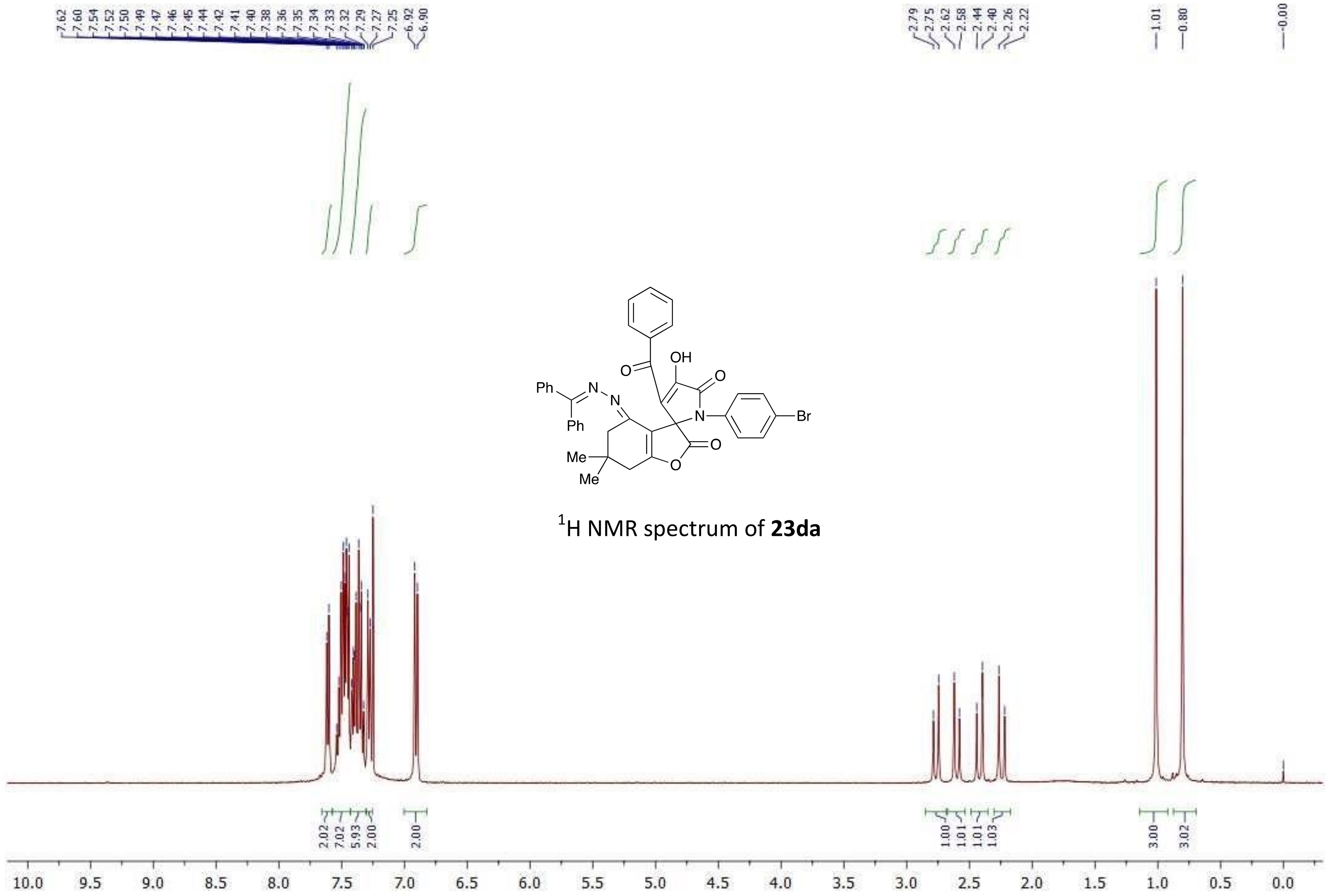

^{1}H NMR spectrum of **23da**

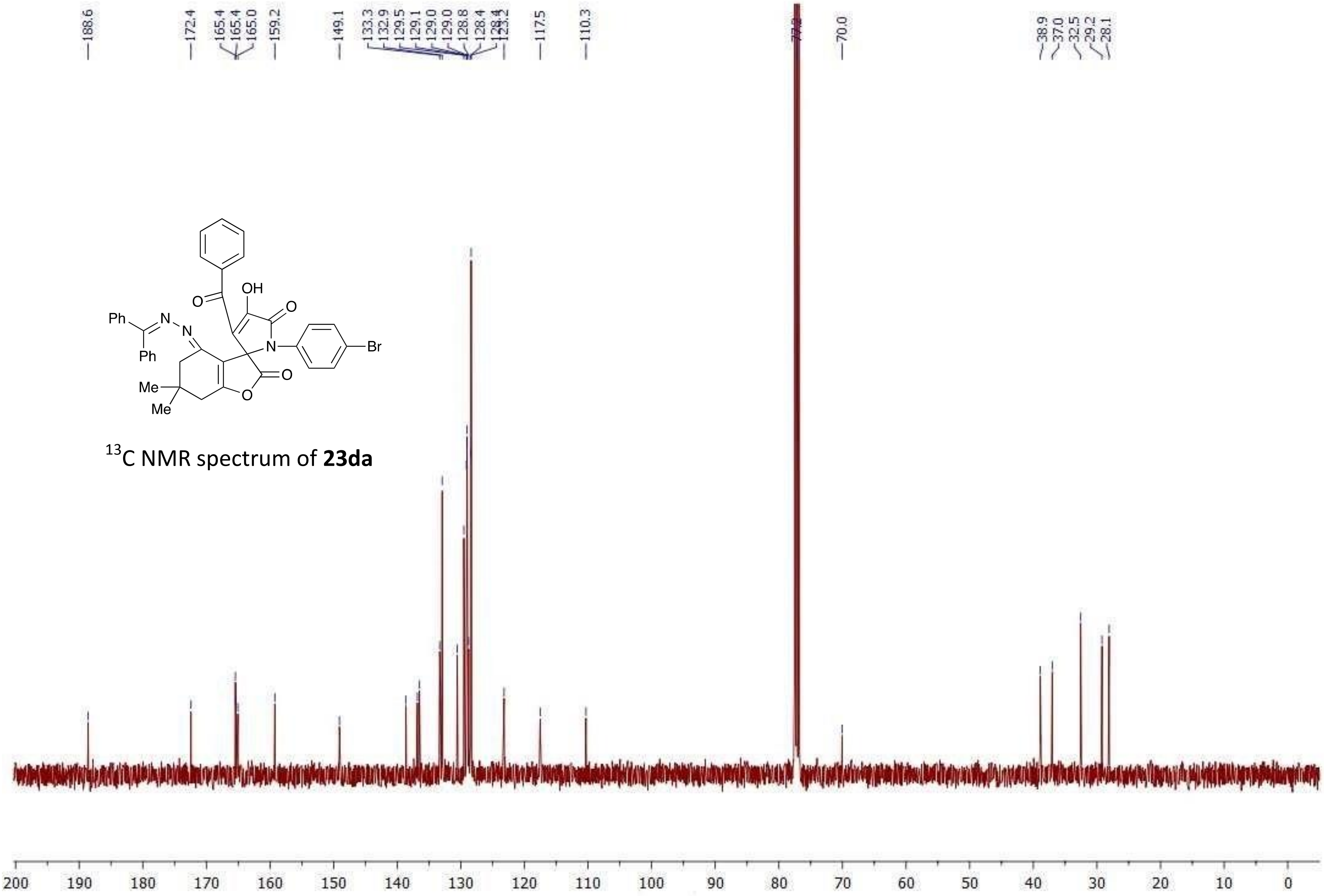

^{13}C NMR spectrum of **23da**

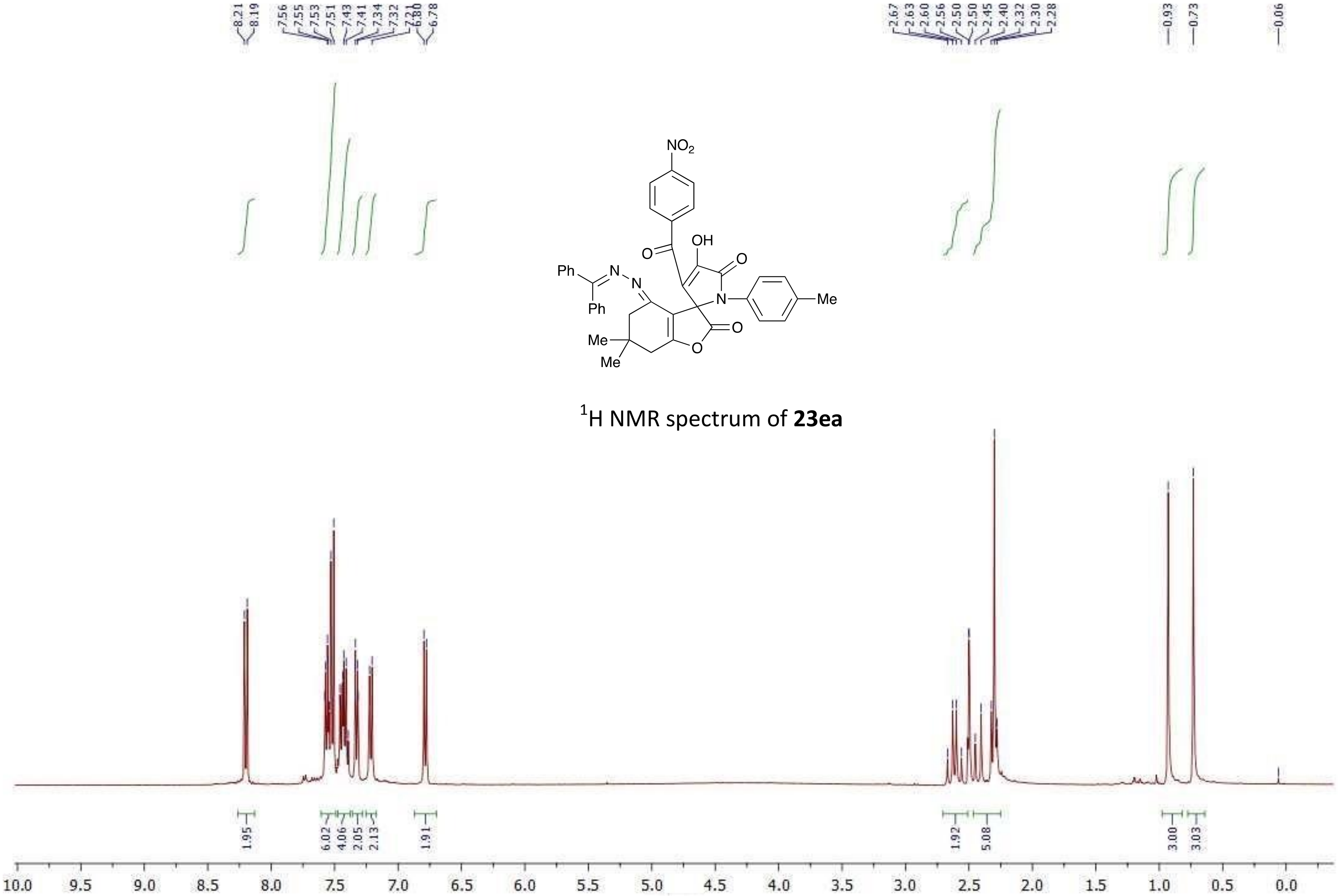

^{1}H NMR spectrum of **23ea**

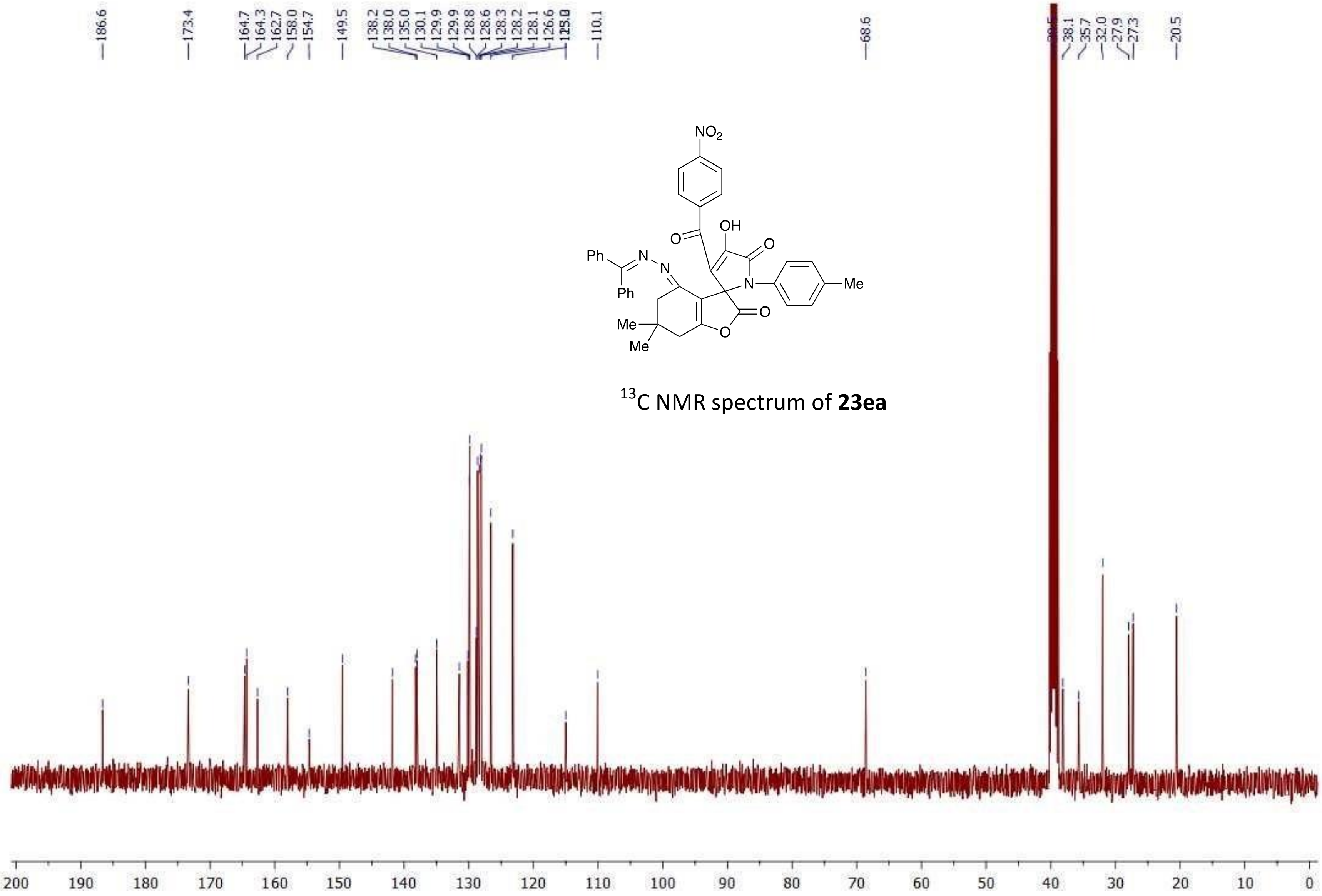

^{13}C NMR spectrum of **23ea**

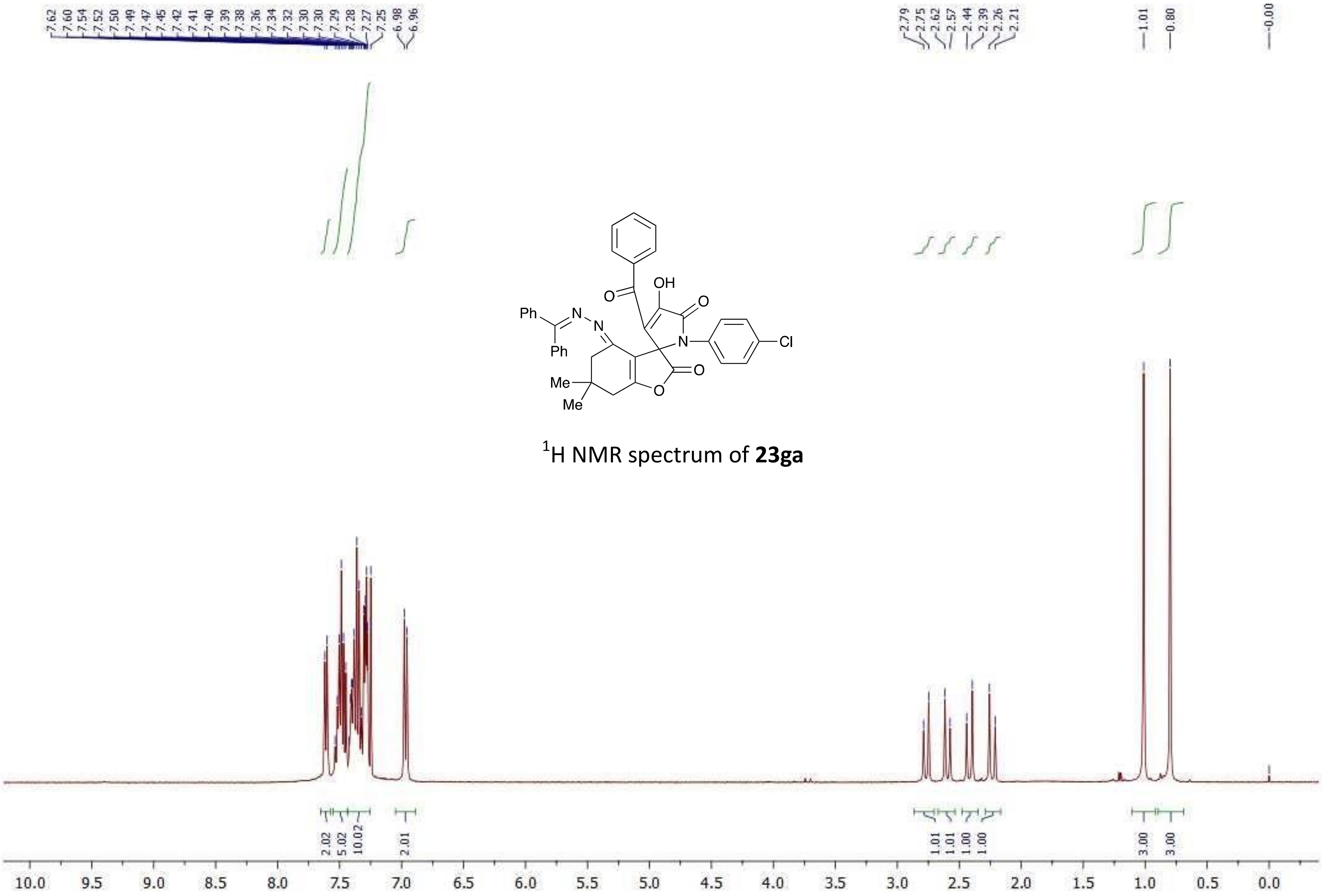

^{1}H NMR spectrum of **23ga**

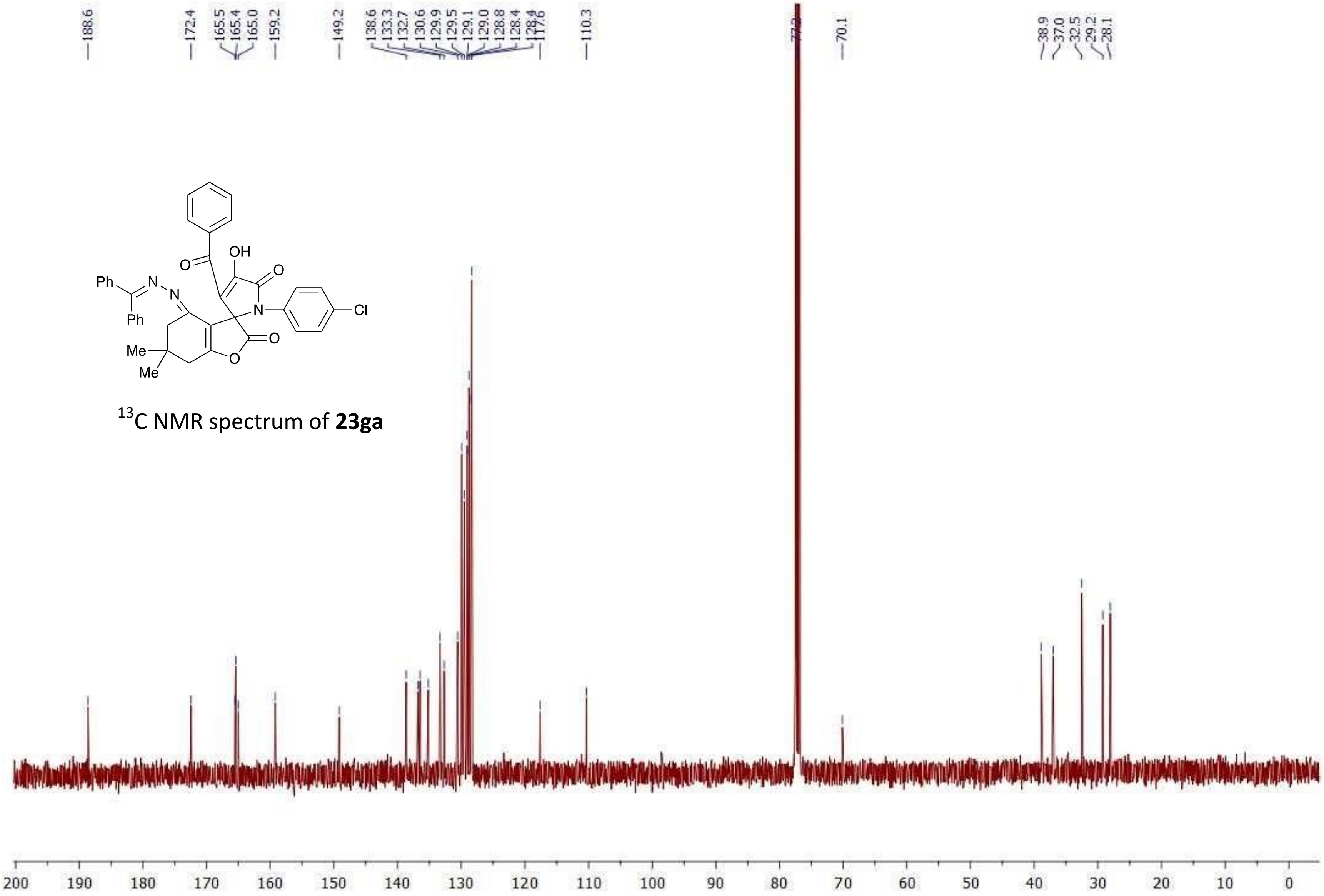

^{13}C NMR spectrum of **23ga**

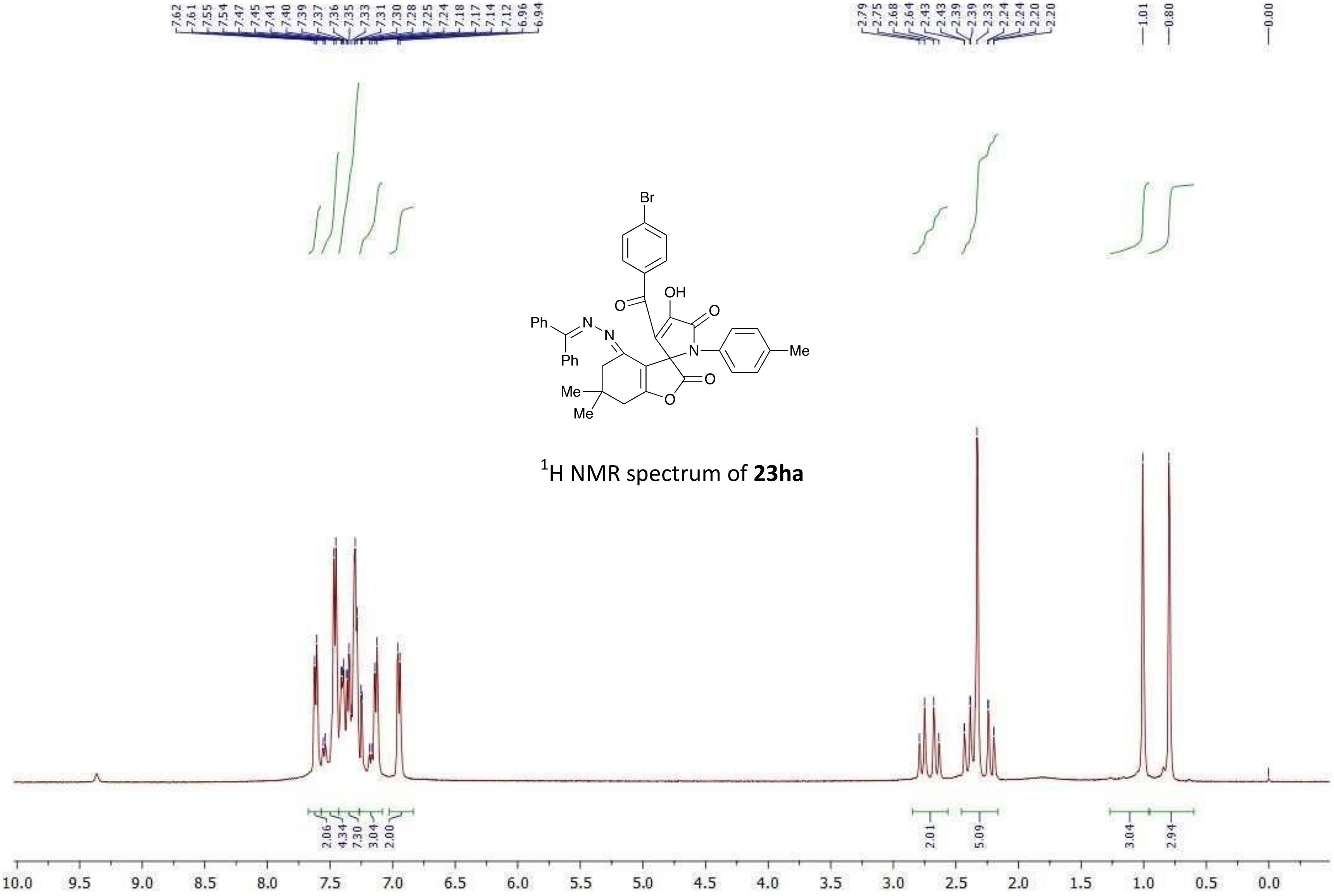

^{1}H NMR spectrum of **23ha**

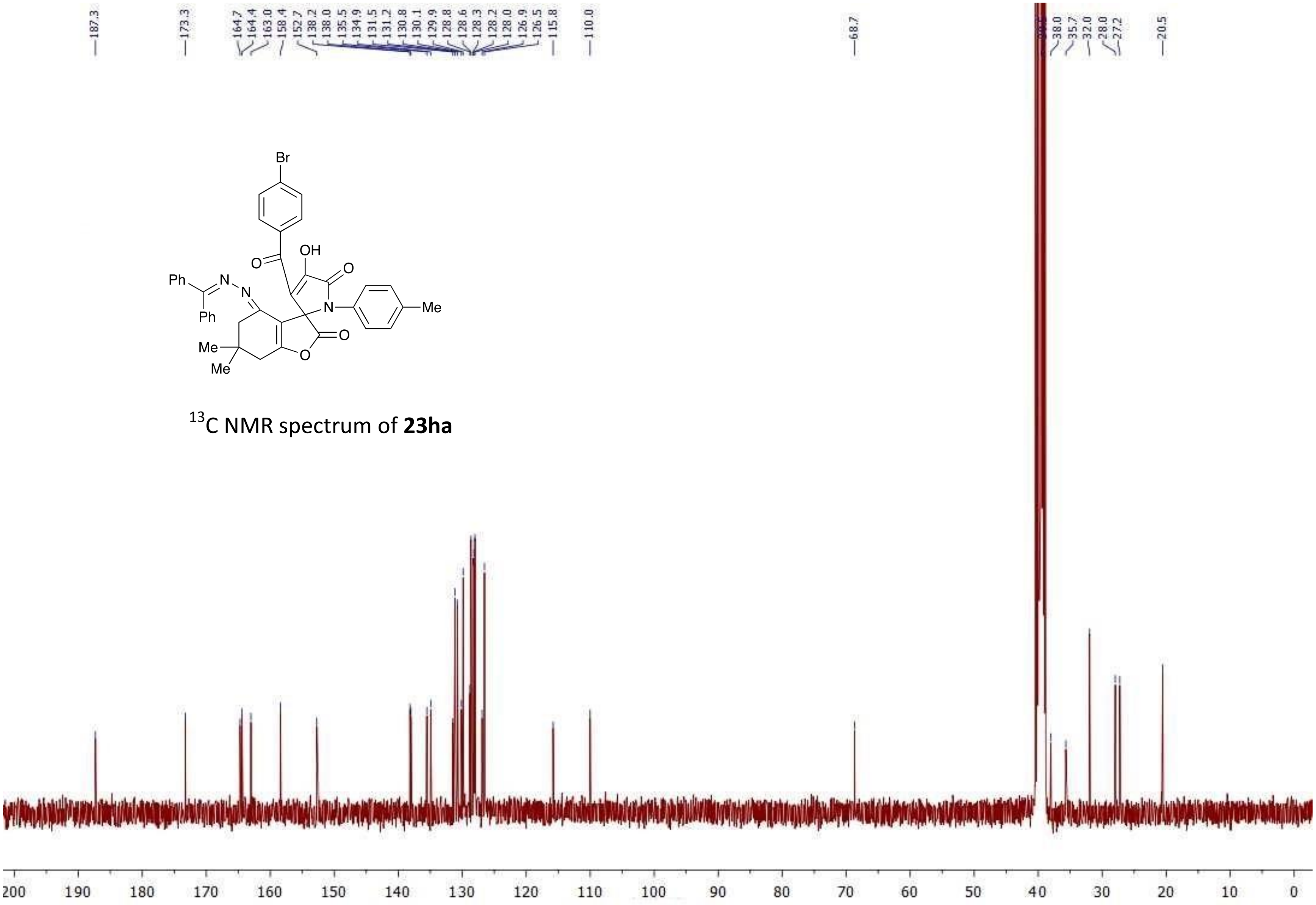

^{13}C NMR spectrum of **23ha**
