# Supplementary material for: Regiodivergent condensation of 5-alkoxycarbonyl-1H-pyrrol-2,3-diones with cyclic ketazinones en route to spirocyclic scaffolds
Source: Beilstein J Org Chem. 2017 Oct 19;13:2179–85. doi: 10.3762/bjoc.13.218 (PMC5669231; doi:10.3762/bjoc.13.218)

checkCIF/PLATON report

Structure factors have been supplied for datablock(s) psu-387da-enhyd

THIS REPORT IS FOR GUIDANCE ONLY. IF USED AS PART OF A REVIEW PROCEDURE FOR PUBLICATION, IT SHOULD NOT REPLACE THE EXPERTISE OF AN EXPERIENCED CRYSTALLOGRAPHIC REFEREE.

No syntax errors found. CIF dictionary Interpreting this report

Datablock: psu-387da-enhyd

```
Bond precision:  C-C = 0.0024 A         Wavelength=0.71073

Cell:           a=11.0448(11)     b=12.2377(13)     c=13.2136(13)
                alpha=99.391(9)   beta=104.791(9)   gamma=103.693(9)
Temperature:    295 K

                Calculated                 Reported
Volume          1629.5(3)                  1629.5(3)
Space group     P -1                       P -1
Hall group      -P 1                       ?
Moiety formula  C39 H31 N3 O5              C39 H31 N3 O5
Sum formula     C39 H31 N3 O5              C39 H31 N3 O5
Mr              621.67                     621.67
Dx,g cm-3       1.267                      1.267
Z               2                          2
Mu (mm-1)       0.085                      0.085
F000            652.0                      652.0
F000'           652.30
h,k,lmax        15,16,18                   14,15,16
Nref            8963                       7531
Tmin,Tmax       0.965,0.979                0.838,1.000
Tmin'           0.958

Correction method= # Reported T Limits: Tmin=0.838 Tmax=1.000
AbsCorr = MULTI-SCAN

Data completeness= 0.840          Theta(max)= 29.360

R(reflections)= 0.0526( 5408)     wR2(reflections)= 0.1468( 7531)

S = 1.069                  Npar= 430
```

The following ALERTS were generated. Each ALERT has the format
test-name_ALERT_alert-type_alert-level.
Click on the hyperlinks for more details of the test.

Alert level C

```
PLAT906_ALERT_3_C Large K value in the Analysis of Variance ......      7.018 Check
PLAT910_ALERT_3_C Missing # of FCF Reflection(s) Below Theta(Min)          8 Note
PLAT911_ALERT_3_C Missing # FCF Refl Between THmin & STh/L=  0.600         3 Report
```

Alert level G

```
PLAT005_ALERT_5_G No Embedded Refinement Details found  in the CIF     Please Do !
PLAT154_ALERT_1_G The s.u.'s on the Cell Angles are Equal ..(Note)      0.009 Degree
PLAT398_ALERT_2_G Deviating  C-O-C  Angle from 120 Deg for O4           107.0 Degree
PLAT793_ALERT_4_G The Model has Chirality at C1      (Centro SPGR)          R Verify
PLAT899_ALERT_4_G SHELXL97   is Deprecated and Succeeded by SHELXL       2014 Note
PLAT912_ALERT_4_G Missing # of FCF Reflections Above STh/L=  0.600       1197 Note
PLAT952_ALERT_5_G Calculated (ThMax) and CIF-Reported Lmax Differ          2 Units
PLAT958_ALERT_1_G Calculated (ThMax) and Actual (FCF) Lmax Differ          2 Units
PLAT961_ALERT_5_G Dataset Contains no Negative Intensities .......     Please Check
PLAT978_ALERT_2_G Number C-C Bonds with Positive Residual Density.         6 Note
```

```
   0 ALERT level A = Most likely a serious problem - resolve or explain
   0 ALERT level B = A potentially serious problem, consider carefully
   3 ALERT level C = Check. Ensure it is not caused by an omission or oversight
  10 ALERT level G = General information/check it is not something unexpected

   2 ALERT type 1 CIF construction/syntax error, inconsistent or missing data
   2 ALERT type 2 Indicator that the structure model may be wrong or deficient
   3 ALERT type 3 Indicator that the structure quality may be low
   3 ALERT type 4 Improvement, methodology, query or suggestion
   3 ALERT type 5 Informative message, check
```

It is advisable to attempt to resolve as many as possible of the alerts in all categories. Often the minor alerts point to easily fixed oversights, errors and omissions in your CIF or refinement strategy, so attention to these fine details can be worthwhile. In order to resolve some of the more serious problems it may be necessary to carry out additional measurements or structure refinements. However, the purpose of your study may justify the reported deviations and the more serious of these should normally be commented upon in the discussion or experimental section of a paper or in the "special_details" fields of the CIF. checkCIF was carefully designed to identify outliers and unusual parameters, but every test has its limitations and alerts that are not important in a particular case may appear. Conversely, the absence of alerts does not guarantee there are no aspects of the results needing attention. It is up to the individual to critically assess their own results and, if necessary, seek expert advice.

Publication of your CIF in IUCr journals

A basic structural check has been run on your CIF. These basic checks will be run on all CIFs submitted for publication in IUCr journals (*Acta Crystallographica*, *Journal of Applied Crystallography*, *Journal of Synchrotron Radiation*); however, if you intend to submit to *Acta Crystallographica Section C* or *E* or *IUCrData*, you should make sure that full publication checks are run on the final version of your CIF prior to submission.

Publication of your CIF in other journals

Please refer to the *Notes for Authors* of the relevant journal for any special instructions relating to CIF submission.

PLATON version of 27/03/2017; check.def file version of 24/03/2017

Datablock psu-387da-enhyd - ellipsoid plot

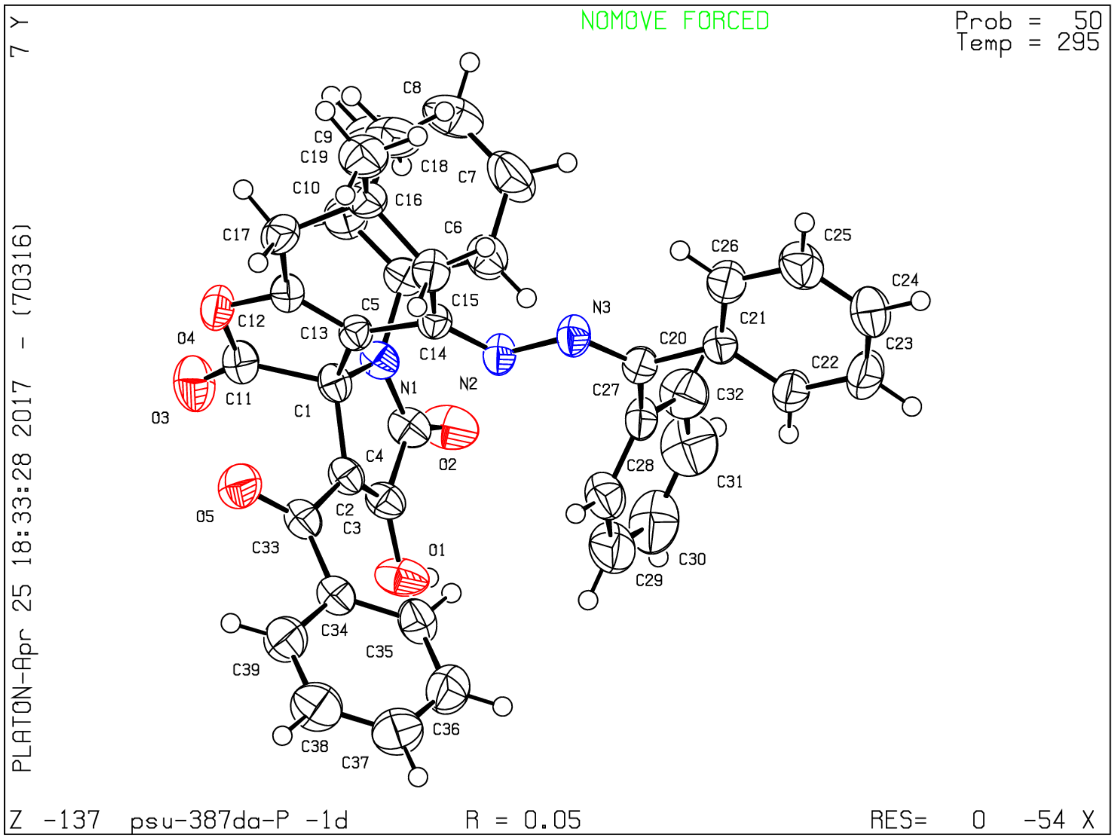

Supplement: File 2 — X-ray CIF files. [file Beilstein_J_Org_Chem-13-2179-s002.zip › PSU-387-ccdc1546064.pdf]
