# Supplementary material for: Regiodivergent condensation of 5-alkoxycarbonyl-1H-pyrrol-2,3-diones with cyclic ketazinones en route to spirocyclic scaffolds
Source: Beilstein J Org Chem. 2017 Oct 19;13:2179–85. doi: 10.3762/bjoc.13.218 (PMC5669231; doi:10.3762/bjoc.13.218)

checkCIF/PLATON report

Structure factors have been supplied for datablock(s) psu-41da-102

THIS REPORT IS FOR GUIDANCE ONLY. IF USED AS PART OF A REVIEW PROCEDURE FOR PUBLICATION, IT SHOULD NOT REPLACE THE EXPERTISE OF AN EXPERIENCED CRYSTALLOGRAPHIC REFEREE.

No syntax errors found. CIF dictionary Interpreting this report

Datablock: psu-41da-102

Bond precision: C-C = 0.0034 A Wavelength=0.71073

Cell:	a=8.7713(12)	b=15.0981(17)	c=15.585(2)
	alpha=109.020(11)	beta=102.851(11)	gamma=106.818(11)
Temperature:	295 K		

	Calculated	Reported
Volume	1749.7(5)	1749.7(4)
Space group	P -1	P -1
Hall group	-P 1	?
Moiety formula	C35 H31 N3 O6, C2 H3 N [+ solvent]	C35 H31 N3 O6, C2 H3 N
Sum formula	C37 H34 N4 O6 [+ solvent]	C37 H34 N4 O6
Mr	630.68	630.68
Dx,g cm-3	1.197	1.197
Z	2	2
Mu (mm-1)	0.082	0.082
F000	664.0	664.0
F000’	664.31	
h,k,lmax	12,21,21	11,19,21
Nref	9948	8318
Tmin,Tmax	0.971,0.984	0.851,1.000
Tmin’	0.968	

Correction method= # Reported T Limits: Tmin=0.851 Tmax=1.000
AbsCorr = MULTI-SCAN

Data completeness= 0.836 Theta(max)= 29.710

R(reflections)= 0.0642(5221) wR2(reflections)= 0.2026(8318)

S = 1.066 Npar= 433

The following ALERTS were generated. Each ALERT has the format
test-name_ALERT_alert-type_alert-level.
Click on the hyperlinks for more details of the test.

Alert level B

```
PLAT990_ALERT_1_B Deprecated RES file style based SQUEEZE job ....          ! Note
```

Alert level C

```
PLAT094_ALERT_2_C Ratio of Maximum / Minimum Residual Density ....       2.24 Report
PLAT244_ALERT_4_C Low    'Solvent' Ueq as Compared to Neighbors of        C1S Check
PLAT906_ALERT_3_C Large K value in the Analysis of Variance ......     12.048 Check
PLAT906_ALERT_3_C Large K value in the Analysis of Variance ......      2.304 Check
PLAT910_ALERT_3_C Missing # of FCF Reflection(s) Below Theta(Min)         10 Note
PLAT918_ALERT_3_C Reflection(s) with I(obs) much Smaller I(calc) .         2 Check
PLAT978_ALERT_2_C Number C-C Bonds with Positive Residual Density.         0 Note
```

Alert level G

```
PLAT002_ALERT_2_G Number of Distance or Angle Restraints on AtSite         2 Note
PLAT005_ALERT_5_G No Embedded Refinement Details found  in the CIF     Please Do !
PLAT072_ALERT_2_G SHELXL First  Parameter in WGHT  Unusually Large      0.10 Report
PLAT154_ALERT_1_G The s.u.'s on the Cell Angles are Equal ..(Note)     0.011 Degree
PLAT605_ALERT_4_G Largest Solvent Accessible VOID in the Structure       135 A**3
PLAT720_ALERT_4_G Number of Unusual/Non-Standard Labels ..........         3 Note
PLAT793_ALERT_4_G The Model has Chirality at C30     (Centro SPGR)         R Verify
PLAT860_ALERT_3_G Number of Least-Squares Restraints .............         1 Note
PLAT869_ALERT_4_G ALERTS Related to the use of SQUEEZE Suppressed          ! Info
PLAT899_ALERT_4_G SHELXL97   is Deprecated and Succeeded by SHELXL      2014 Note
PLAT912_ALERT_4_G Missing # of FCF Reflections Above STh/L=  0.600      1496 Note
PLAT951_ALERT_5_G Calculated (ThMax) and CIF-Reported Kmax Differ          2 Units
PLAT957_ALERT_1_G Calculated (ThMax) and Actual (FCF) Kmax Differ          2 Units
PLAT961_ALERT_5_G Dataset Contains no Negative Intensities .......     Please Check
```

---

0 **ALERT level A** = Most likely a serious problem - resolve or explain
1 **ALERT level B** = A potentially serious problem, consider carefully
7 **ALERT level C** = Check. Ensure it is not caused by an omission or oversight
14 **ALERT level G** = General information/check it is not something unexpected

3 ALERT type 1 CIF construction/syntax error, inconsistent or missing data
4 ALERT type 2 Indicator that the structure model may be wrong or deficient
5 ALERT type 3 Indicator that the structure quality may be low
7 ALERT type 4 Improvement, methodology, query or suggestion
3 ALERT type 5 Informative message, check

It is advisable to attempt to resolve as many as possible of the alerts in all categories. Often the minor alerts point to easily fixed oversights, errors and omissions in your CIF or refinement strategy, so attention to these fine details can be worthwhile. In order to resolve some of the more serious problems it may be necessary to carry out additional measurements or structure refinements. However, the purpose of your study may justify the reported deviations and the more serious of these should normally be commented upon in the discussion or experimental section of a paper or in the "special_details" fields of the CIF. checkCIF was carefully designed to identify outliers and unusual parameters, but every test has its limitations and alerts that are not important in a particular case may appear. Conversely, the absence of alerts does not guarantee there are no aspects of the results needing attention. It is up to the individual to critically assess their own results and, if necessary, seek expert advice.

Publication of your CIF in IUCr journals

A basic structural check has been run on your CIF. These basic checks will be run on all CIFs submitted for publication in IUCr journals (*Acta Crystallographica*, *Journal of Applied Crystallography*, *Journal of Synchrotron Radiation*); however, if you intend to submit to *Acta Crystallographica Section C* or *E* or *IUCrData*, you should make sure that full publication checks are run on the final version of your CIF prior to submission.

Publication of your CIF in other journals

Please refer to the *Notes for Authors* of the relevant journal for any special instructions relating to CIF submission.

PLATON version of 27/03/2017; check.def file version of 24/03/2017

Datablock psu-41da-102 - ellipsoid plot

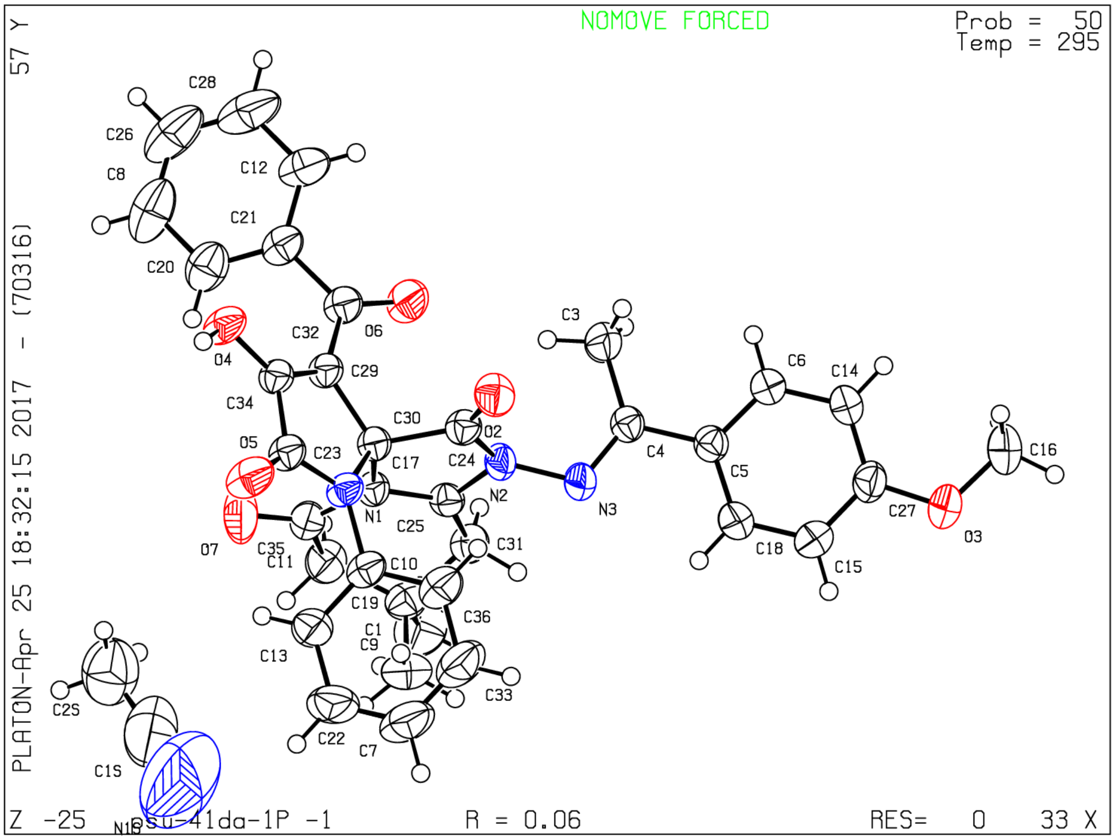

Supplement: File 2 — X-ray CIF files. [file Beilstein_J_Org_Chem-13-2179-s002.zip › PSU-41--ccdc1546063.pdf]
