# Supplementary material for: Regiodivergent condensation of 5-alkoxycarbonyl-1H-pyrrol-2,3-diones with cyclic ketazinones en route to spirocyclic scaffolds
Source: Beilstein J Org Chem. 2017 Oct 19;13:2179–85. doi: 10.3762/bjoc.13.218 (PMC5669231; doi:10.3762/bjoc.13.218)

checkCIF/PLATON report

Structure factors have been supplied for datablock(s) psu-478da-139

THIS REPORT IS FOR GUIDANCE ONLY. IF USED AS PART OF A REVIEW PROCEDURE FOR PUBLICATION, IT SHOULD NOT REPLACE THE EXPERTISE OF AN EXPERIENCED CRYSTALLOGRAPHIC REFEREE.

No syntax errors found. CIF dictionary Interpreting this report

Datablock: psu-478da-139

```
Bond precision:  C-C = 0.0036 A          Wavelength=0.71073

Cell:            a=24.667(5)     b=5.8372(7)         c=27.124(7)
                 alpha=90        beta=116.74(3)      gamma=90
Temperature:     295 K

                Calculated                  Reported
Volume          3487.8(15)                  3487.9(14)
Space group     P 21/c                      P 1 21/c 1
Hall group      -P 2ybc                     -P 2ybc
Moiety formula  C21 H22 N2 O                C21 H22 N2 O
Sum formula     C21 H22 N2 O                C21 H22 N2 O
Mr              318.41                      318.40
Dx,g cm-3       1.213                       1.213
Z               8                           8
Mu (mm-1)       0.075                       0.075
F000            1360.0                      1360.0
F000'           1360.50
h,k,lmax        34,8,37                     32,8,37
Nref            9766                        8608
Tmin,Tmax       0.994,0.996                 0.696,1.000
Tmin'           0.960

Correction method= # Reported T Limits: Tmin=0.696 Tmax=1.000
AbsCorr = MULTI-SCAN

Data completeness= 0.881          Theta(max)= 29.582

R(reflections)= 0.0578( 3920)     wR2(reflections)= 0.1534( 8608)

S = 0.980                 Npar= 445
```

The following ALERTS were generated. Each ALERT has the format
test-name_ALERT_alert-type_alert-level.
Click on the hyperlinks for more details of the test.

Alert level C

```
PLAT026_ALERT_3_C Ratio Observed / Unique Reflections (too) Low ..         46 %
PLAT241_ALERT_2_C High   'MainMol' Ueq as Compared to Neighbors of         C4 Check
PLAT242_ALERT_2_C Low    'MainMol' Ueq as Compared to Neighbors of         C2 Check
PLAT331_ALERT_2_C Small Average Phenyl   C-C Dist. C2     -C7            1.37 Ang.
PLAT906_ALERT_3_C Large K value in the Analysis of Variance ......     21.945 Check
PLAT906_ALERT_3_C Large K value in the Analysis of Variance ......      3.973 Check
PLAT906_ALERT_3_C Large K value in the Analysis of Variance ......      2.007 Check
PLAT910_ALERT_3_C Missing # of FCF Reflection(s) Below Theta(Min)           8 Note
PLAT911_ALERT_3_C Missing # FCF Refl Between THmin & STh/L=  0.600         16 Report
PLAT978_ALERT_2_C Number C-C Bonds with Positive Residual Density.          0 Note
```

Alert level G

```
PLAT912_ALERT_4_G Missing # of FCF Reflections Above STh/L=  0.600       1098 Note
PLAT933_ALERT_2_G Number of OMIT Records in Embedded .res File ...          6 Note
PLAT950_ALERT_5_G Calculated (ThMax) and CIF-Reported Hmax Differ           2 Units
PLAT956_ALERT_1_G Calculated (ThMax) and Actual (FCF) Hmax Differ           2 Units
```

0 **ALERT level A** = Most likely a serious problem - resolve or explain
0 **ALERT level B** = A potentially serious problem, consider carefully
10 **ALERT level C** = Check. Ensure it is not caused by an omission or oversight
4 **ALERT level G** = General information/check it is not something unexpected

1 ALERT type 1 CIF construction/syntax error, inconsistent or missing data
5 ALERT type 2 Indicator that the structure model may be wrong or deficient
6 ALERT type 3 Indicator that the structure quality may be low
1 ALERT type 4 Improvement, methodology, query or suggestion
1 ALERT type 5 Informative message, check

It is advisable to attempt to resolve as many as possible of the alerts in all categories. Often the minor alerts point to easily fixed oversights, errors and omissions in your CIF or refinement strategy, so attention to these fine details can be worthwhile. In order to resolve some of the more serious problems it may be necessary to carry out additional measurements or structure refinements. However, the purpose of your study may justify the reported deviations and the more serious of these should normally be commented upon in the discussion or experimental section of a paper or in the "special_details" fields of the CIF. checkCIF was carefully designed to identify outliers and unusual parameters, but every test has its limitations and alerts that are not important in a particular case may appear. Conversely, the absence of alerts does not guarantee there are no aspects of the results needing attention. It is up to the individual to critically assess their own results and, if necessary, seek expert advice.

Publication of your CIF in IUCr journals

A basic structural check has been run on your CIF. These basic checks will be run on all CIFs submitted for publication in IUCr journals (*Acta Crystallographica*, *Journal of Applied Crystallography*, *Journal of Synchrotron Radiation*); however, if you intend to submit to *Acta Crystallographica Section C* or *E* or *IUCrData*, you should make sure that full publication checks are run on the final version of your CIF prior to submission.

Publication of your CIF in other journals

Please refer to the *Notes for Authors* of the relevant journal for any special instructions relating to CIF submission.

PLATON version of 27/03/2017; check.def file version of 24/03/2017

Datablock psu-478da-139 - ellipsoid plot

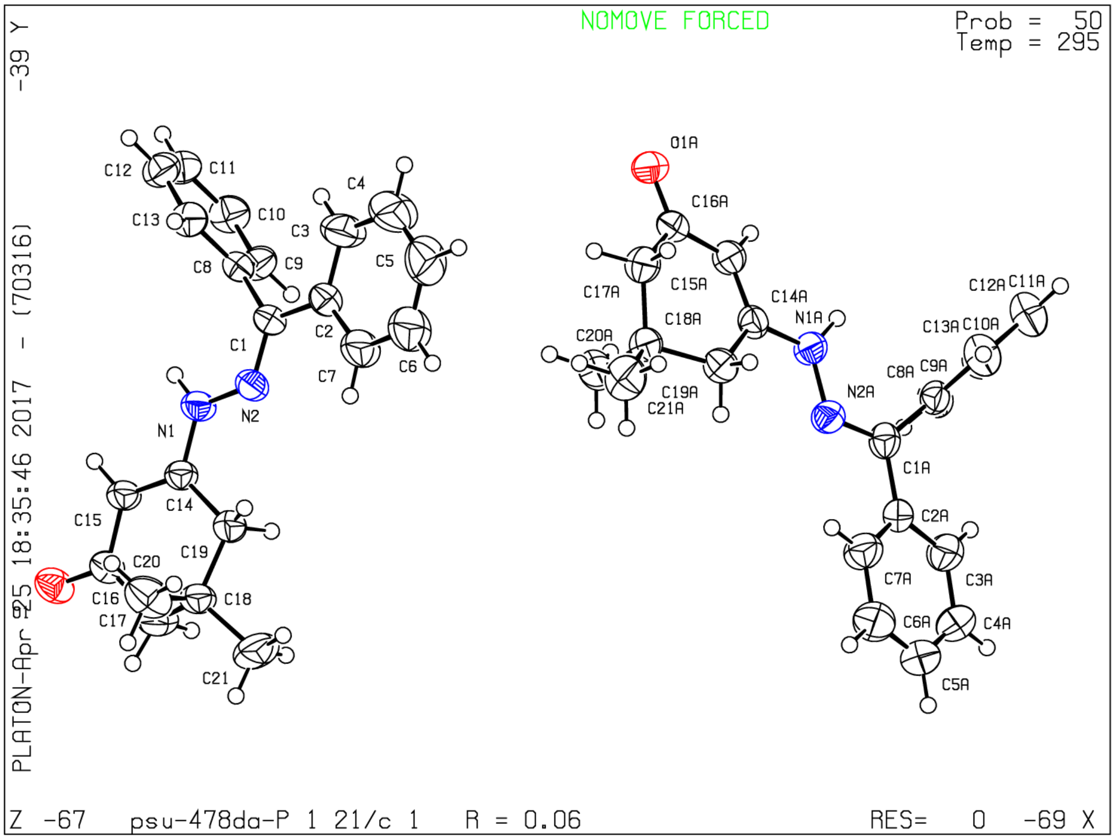

Supplement: File 2 — X-ray CIF files. [file Beilstein_J_Org_Chem-13-2179-s002.zip › PSU-478-ccdc1546065.pdf]
